# Efficacy and Safety of Direct Oral Anticoagulants versus Warfarin in Obese Patients (BMI ≥ 30 kg/m^2^) with Atrial Fibrillation or Venous Thromboembolism: An Updated Systematic Review and Meta-Analysis

**DOI:** 10.3390/jcm13133784

**Published:** 2024-06-27

**Authors:** Paschalis Karakasis, Nikolaos Ktenopoulos, Konstantinos Pamporis, Marios Sagris, Stergios Soulaidopoulos, Maria Gerogianni, Ioannis Leontsinis, George Giannakoulas, Dimitris Tousoulis, Nikolaos Fragakis, Konstantinos Tsioufis

**Affiliations:** 1 Second Department of Cardiology, Hippokration General Hospital, Aristotle University of Thessaloniki, 54124 Thessaloniki, Greece; pakar15@hotmail.com (P.K.); giannisleontsinis@gmail.com (I.L.); fragakis.nikos@googlemail.com (N.F.); 2 School of Medicine, Hippokration General Hospital, National and Kapodistrian University of Athens, 11527 Athens, Greece; nikosktenop@gmail.com (N.K.); konstantinospab@gmail.com (K.P.); soulaidopoulos@gmail.com (S.S.); tousouli@med.uoa.gr (D.T.); ktsioufis@gmail.com (K.T.); 3 Department of Hygiene, Social-Preventive Medicine & Medical Statistics, Medical School, Aristotle University of Thessaloniki, 54124 Thessaloniki, Greece; 4 Endocrine Unit, 2nd Propaedeutic Department of Internal Medicine, School of Medicine, Research Institute and Diabetes Center, Attikon University Hospital, National and Kapodistrian University of Athens, 12641 Athens, Greece; gerogianni.e.maria@gmail.com; 5 First Department of Cardiology, AHEPA University Hospital, Aristotle University of Thessaloniki, 54124 Thessaloniki, Greece; g.giannakoulas@gmail.com

**Keywords:** direct oral anticoagulants, vitamin-k antagonist, warfarin, obesity, anticoagulation, atrial fibrillation, venous thromboembolism

## Abstract

**Background**: Real-world data show limited utilization of direct oral anticoagulants (DOACs) in obese patients (body mass index [BMI] ≥ 30 kg/m^2^) due to concerns regarding their efficacy and safety in this demographic. **Aim**: This review aimed to consolidate current evidence on the efficacy and safety of DOACs versus warfarin in obese patients with non-valvular atrial fibrillation (AF) or venous thromboembolism (VTE). The primary efficacy outcome assessed a composite of all-cause mortality, stroke, systemic embolism (SE), and myocardial infarction (MI). **Methods**: A systematic search was conducted in MEDLINE, SCOPUS, and Cochrane databases from inception to December 28, 2023. Data were synthesized using random-effects meta-analysis. **Results**: A total of 35 studies involving 434,320 participants were analyzed. DOAC use was associated with a significant reduction in the risk of the composite outcome (RR = 0.80, 95% CI [0.65, 0.98], I^2^ = 95%), hemorrhagic stroke (RR = 0.58, 95% CI [0.38, 0.88], I^2^ = 92%), major bleeding (RR = 0.76, 95% CI [0.63, 0.92], I^2^ = 94%), gastrointestinal bleeding (RR = 0.59, 95% CI [0.49, 0.72], I^2^ = 88%), and intracranial bleeding (RR = 0.45, 95% CI [0.34, 0.60], I^2^ = 44%) compared to warfarin. A non-significant benefit of DOACs was observed for all-cause mortality, MI, the composite of stroke or SE, ischemic stroke, SE, VTE, and minor bleeding compared to warfarin. Subgroup analysis indicated no significant effect modification based on the indication for anticoagulation or study design. **Conclusions**: DOACs demonstrated a favorable efficacy and safety profile in obese individuals compared to warfarin.

## 1. Introduction

Obesity, defined as a body mass index (BMI) above or equal to 30 kg/m^2^, is a multifaceted and escalating public health challenge that has reached epidemic proportions globally [1]. The prevalence of obesity is projected to rise significantly from 14% to 24% within the population by the year 2035, affecting nearly 2 billion individuals worldwide [1].

Atrial fibrillation (AF) is a prominent contributor to stroke, heart failure, and cardiovascular disease, with the associated adverse events not only incurring substantial healthcare costs but also imposing a significant public health burden [2]. Recent data from the Global Burden of Disease study highlight the substantial contribution of AF to the total number of age-standardized disability-adjusted life years (DALYs) attributable to cardiovascular disease (CVD) in the year 2022, amounting to 102.9 per 100,000 individuals on a global scale [3].

The correlation between obesity and AF has long been established. The Framingham Heart Study, in particular, elucidated a distinct association between these two conditions, revealing an approximately 4% increase in the risk for AF per incremental unit rise in BMI [4]. Based on a recent Mendelian randomization study, one standard deviation (SD) increment of BMI causally increased the risk for AF by 42.5% [5]. Furthermore, numerous studies have shown that individuals with obesity pose a significantly higher multiplicative risk for VTE compared to those with a BMI within the normal range [6,7].

Over the last decade, direct oral anticoagulants (DOACs) have emerged as the preferred therapeutic choice for preventing and treating venous thromboembolism (VTE) and for stroke prevention in non-valvular AF [8]. Compared to warfarin, DOACs offer several advantages, encompassing a wider therapeutic window, decreased monitoring requirements, standardized dosing, rapid onset of action, and reduced potential for drug–drug and drug–food interactions [9,10]. Nevertheless, there is a scarcity of comprehensive data pertaining to the clinical pharmacokinetics, pharmacodynamics, efficacy, and safety in individuals with obesity, primarily because this particular group of patients is markedly underrepresented in most pivotal DOAC trials.

These gaps in knowledge may potentially impede the widespread adoption of DOACs in this specific population. Therefore, the present study sought to systematically review and quantitatively synthesize the available evidence on the efficacy and safety of DOACs in obese individuals with AF or VTE.

## 2. Methods

The present systematic review (SR) was performed according to the guidelines of the Cochrane Handbook for SRs [11] and on the basis of a preregistered protocol (https://osf.io/nvxy3/, DOI 10.17605/OSF.IO/NVXY3). Reporting was performed based on the Preferred Reporting Items for Systematic reviews and Meta-Analyses (PRISMA) 2020 statement [12]. A checklist of the reported PRISMA 2020 items is presented in Table S1.

### 2.1. Information Sources and Search Strategy

Systematic searches were conducted in MEDLINE (via Pubmed), Scopus, and the Cochrane Library from inception up to 28 December 2023 without language restrictions (full search strategy at Table S2). Reference lists from selected studies and pertinent reviews and abstracts from international conferences during the last five years were also scrutinized.

### 2.2. Eligibility Criteria

#### 2.2.1. Population and Interventions

Adult (≥18 years old) obese patients (BMI ≥ 30 kg/m^2^) with non-valvular AF or VTE requiring oral anticoagulation treatment were eligible. Eligible studies should compare any DOAC to warfarin in terms of effectiveness and/or safety.

#### 2.2.2. Outcomes

The primary efficacy outcomes of this study were (i) the composite endpoint of any stroke or systemic embolism (SE) or myocardial infarction (MI) or all-cause mortality and (ii) all-cause mortality. Secondary efficacy outcomes included the composite endpoint of any stroke or SE, MI, ischemic stroke, hemorrhagic stroke, or SE and VTE. The primary safety outcome was major bleeding while secondary safety outcomes included minor bleeding, gastrointestinal bleeding, and intracranial bleeding. For each outcome, the definitions used in each primary study were adopted.

#### 2.2.3. Study Design

Eligible studies were RCTs of phase II or more and observational studies of any duration including a minimum sample size of ten patients.

#### 2.2.4. Exclusion Criteria

Records were excluded based on the following criteria:

- Case reports/case series, narrative reviews;
- Expert opinions, dissertations, protocols;
- Including animals and/or in vitro studies.

### 2.3. Selection Process

All records retrieved from the search strategy were screened by title and abstract from two investigators independently. Subsequently, the same authors also independently screened the full texts of the remaining studies. Discrepancies in any stage were resolved by discussion or consultation with a third reviewer. The EndNote X7 software was used for reference management.

### 2.4. Data Extraction

A data extraction form was created, piloted, and standardized after discussion and calibration exercises. The following variables were extracted: first author, publication year, title, journal’s title, study type, randomized participants in each group, inclusion/exclusion criteria, demographic population characteristics, comorbidities, concomitant medication use, intervention(s), comparator, efficacy and safety outcomes, number of events, and patients in each group (priority to ITT analysis).

### 2.5. Quality Assessments

Methodological quality of the included studies was evaluated using the modified Newcastle–Ottawa Scale (NOS) [13] for observational studies and the revised Cochrane Risk of Bias 2 (RoB 2) tool [14] for RCTs. The NOS encompasses three major domains (selection, comparability, exposure) and scores range between 0 and 9, with higher scores representing better quality. The evaluation was performed according to the instructions provided by each tool’s authors and using all prespecified domains. Methodological quality was evaluated by two authors independently and any disagreements were resolved through discussion or involvement of a third author.

### 2.6. Data Analysis

All analyses were performed using R Statistical Software (v. 4.2). Frequencies with percentages (%) are presented for categorical variables, means with standard deviations (SD) for Gaussian continuous variables, and medians with interquartile range (IQR) for non-normally distributed continuous variables. The risk ratios (RR) and 95% confidence intervals (CIs) were calculated using the DerSimonian–Laird estimator for random effects models and the Mantel–Haenszel method for fixed effects models. In the case of zero events in a treatment arm, a continuity correction was applied by adding 0.5 in each cell [15]. Heterogeneity was assessed using the I^2^ and formally tested with the Cochran’s Q. I-squared values range from zero to one, with values closer to one indicating larger heterogeneity [16]. Small study effects (including publication bias) for each outcome were visually assessed with funnel plots and formally tested with the Egger’s test. Subgroup analyses were performed for all outcomes based on the indication for anticoagulation (AF, VTE, AF, or VTE), the type of study (observational vs. RCT), the inclusion criterion for minimum BMI (BMI ≥ 30 vs. BMI ≥ 40), follow-up duration (≥12 months vs. <12 months), study quality (high quality vs. non-high quality), and age (≥65 years, <65 years) and differences between subgroups were tested with the Cochran’s Q test. Meta-regression models were applied for the primary outcomes to identify potential effect modifiers. Additionally, a sensitivity analysis was performed for all outcomes through recalculation of the overall random-effects estimate by successively excluding one study at a time to identify potentially influential studies. For all statistical tests, a two-tailed *p*-value less than 0.05 was considered statistically significant.

## 3. Results

### 3.1. Selection Process and Study Characteristics

After removing duplicates, 861 references were initially screened by title and abstract, of which 76 were subsequently evaluated through full-text screening. Of these, 41 were excluded and 35 studies finally met the established eligibility criteria [17,18,19,20,21,22,23,24,25,26,27,28,29,30,31,32,33,34,35,36,37,38,39,40,41,42,43,44,45,46,47,48,49,50,51] with 434,320 participants (207,628 in the DOAC arm and 226,692 in the warfarin arm) being included in the final analysis (Figure S1).

The characteristics of analyzed studies are presented in Table S3. Publication date ranged between 2015 and 2022 and most of the studies were observational cohorts (33/35 [94%]), while the remaining were RCTs (2/35 [6%]). Participants’ age ranged between 53.3 and 74.9 years, while male percentage between 19 and 99%. Most individuals were hypertensive (53–99%), while the prevalence of diabetes ranged from 20 to 65%. The percentage of dyslipidemia ranged between 28 and 85% while the corresponding percentages for cancer and renal disease were 2–29% and 5–55%, accordingly. The participants were followed up over a median period of 5 years. Regarding the methodological quality of included studies, the median (IQR) value of NOS was 8 (7–9) (Table S4). The included RCTs were assessed as having low risk of bias in all predetermined domains of the Cochrane RoB 2 tool.

### 3.2. Efficacy Outcomes

A summary of the analysis for each efficacy outcome is presented in Table 1A.

**Figure 1:**
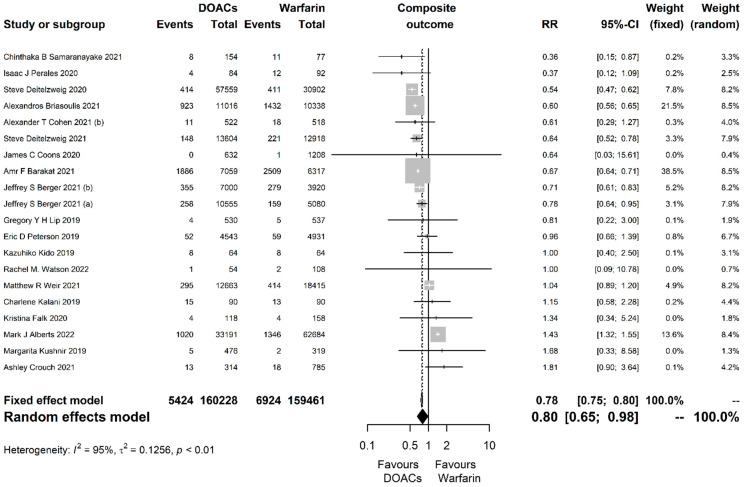
Forest plot of the comparison between direct oral anticoagulants (DOACs) and warfarin for the composite primary efficacy outcome. RR, risk ratio; CI, confidence interval.

For the primary composite outcome, 20 studies including 319,689 participants were analyzed with DOACs being associated with significantly greater risk reduction compared to warfarin (RR = 0.80, 95% CI [0.65, 0.98], P = 0.033; I^2^ = 95%, *p* < 0.001; Figure 1).

**Figure 2:**
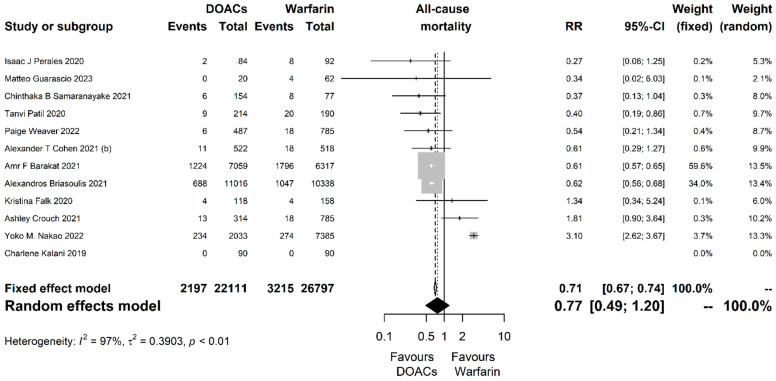
Forest plot of the comparison between direct oral anticoagulants (DOACs) and warfarin for the outcome of all-cause mortality. RR, risk ratio; CI, confidence interval.

Regarding all-cause mortality, 12 studies with 48,908 participants were analyzed and DOACs were associated with a non-significant 23% reduction compared to warfarin (RR = 0.77, 95% CI [0.49, 1.20], P = 0.247; I^2^ = 97%, *p* < 0.001; Figure 2).

In the composite outcome of any type of stroke or SE, 18 studies with 331,210 participants were included and DOACs did not differ significantly compared to warfarin (RR = 0.90, 95% CI [0.71, 1.15], P = 0.409; I^2^ = 94%, *p* < 0.001; Figure S2).

For the outcome of MI, three studies with 34,910 participants were analyzed and DOACs were associated with non-significant 18% reduction (RR = 0.82, 95% CI [0.51, 1.32], P = 0.413; I^2^ = 88%, *p* < 0.001; Figure S3).

Regarding ischemic stroke, 13 studies and 317292 patients were included with DOACs demonstrating similar efficacy compared to warfarin (RR = 0.94, 95% CI [0.72, 1.22], P = 0.649; I^2^ = 93%, *p* < 0.001; Figure S4).

Nevertheless, in the analysis of hemorrhagic stroke involving nine studies and 305,061 participants, DOACs demonstrated greater effectiveness compared to warfarin (RR = 0.58, 95% CI [0.38, 0.88], P = 0.011; I^2^ = 92%, *p* < 0.001; Figure S5).

For the outcome of SE, seven studies with 268,722 participants were included in the analysis. A non-significant 19% reduction was observed for DOACs compared to warfarin (RR = 0.81, 95% CI [0.63, 1.05], P = 0.113; I^2^ = 26%, *p* = 0.68; Figure S6).

For the outcome of VTE, 20 studies with 110,160 participants were analyzed with DOACs presenting a non-significant superiority (RR = 0.80, 95% CI [0.63, 1], P = 0.052; I^2^ = 87%, *p* < 0.001; Figure S7). In general, no evidence of small study effects (including publication bias) was noticed based on the funnel plots and the results of the Egger’s test for any of the efficacy outcomes (Figure S8).

### 3.3. Safety Outcomes

A summary of the analysis for each safety outcome is presented in Table 1B.

**Figure 3:**
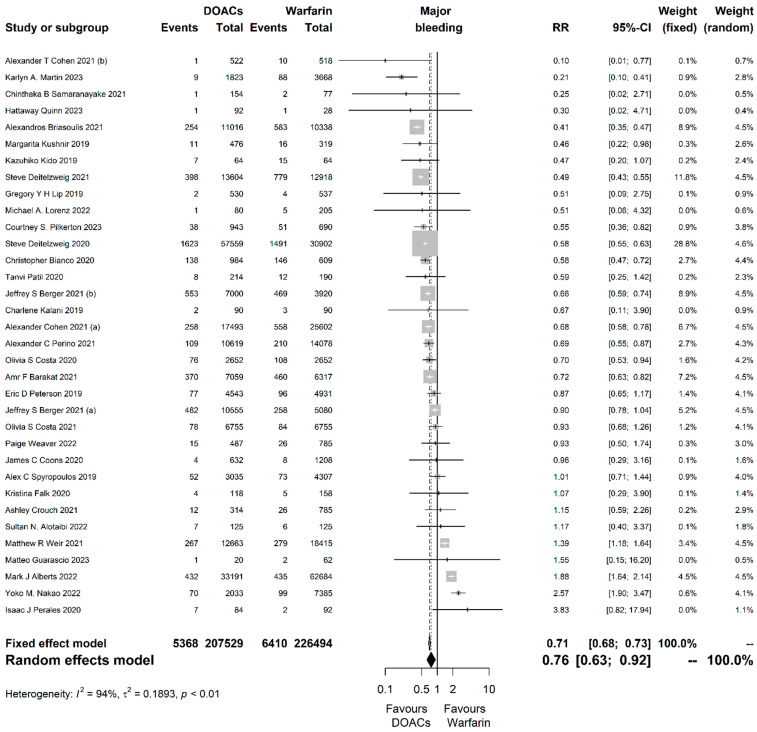
Forest plot of the comparison between direct oral anticoagulants (DOACs) and warfarin for the primary safety outcome of major bleeding. RR, risk ratio; CI, confidence interval.

For the primary safety outcome of major bleeding, 34 studies encompassing 434,023 participants were analyzed and DOACs seemed to be associated with significantly less bleeding events compared to warfarin (RR = 0.76, 95% CI [0.63, 0.92], P = 0.004; I^2^ = 94%, *p* < 0.001; Figure 3).

Regarding minor bleeding, the analysis of 14 studies and 76,479 participants did not demonstrate any significant differences among DOACs and warfarin (RR = 0.87, 95% CI [0.70, 1.07], P = 0.257; I^2^ = 76%, *p* < 0.001; Figure S9).

Regarding gastrointestinal bleeding, the analysis of five studies and 181,272 participants revealed a significant 41% reduced risk with DOACs compared to warfarin (RR = 0.59, 95% CI [0.49, 0.72], P < 0.001; I^2^ = 88%, *p* < 0.001; Figure S10).

In the outcome of intracranial bleeding, six studies with 165,384 participants demonstrated a safer profile of DOACs compared to warfarin (RR = 0.45, 95% CI [0.34, 0.60), P < 0.001; I^2^ = 44%, *p* = 0.11; Figure S11). Of note, the funnel plots and the Egger’s tests for the safety outcomes (Figure S12) did not reveal evidence of small study effects (including publication bias).

### 3.4. Subgroup Analysis, Sensitivity Analysis, and Meta-Regression

The subgroup analysis based on the indication for anticoagulation did not reveal any significant differences among subgroups in any of the efficacy (Figures S13–S20) or safety (Figures S21–S24) outcomes. Accordingly, no significant subgroup differences were noticed in the subgroup analysis according to the type of study design (Figures S25–S30), the minimum BMI used as inclusion criterion (Figures S31–S40), the follow-up duration (Figures S41–S52), study quality (Figures S53–S62), or age (Figures S63–S74). The leave-one-out sensitivity analysis did not provide indications of particularly influential studies for the outcomes considered (Figure S75–S86).

The meta-regression analysis demonstrated a significant effect modification in the composite outcome based on the percentage of patients with cancer (Table S5). Accordingly, significant effect modification was noted based on the percentage of patients with cancer and the percentage of patients with renal disease for the outcome of all-cause mortality. No significant effect modifiers were found for the outcome of major bleeding.

## 4. Discussion

The present study constitutes the most extensive meta-analysis regarding the efficacy and safety of DOACs in individuals with a BMI ≥ 30 kg/m^2^. Our findings support that the use of DOACs in the obese population is associated with a significant reduction by 20% in the combined risk of any stroke, SE, MI, or all-cause mortality compared to warfarin. Notably, when analyzing the different components of the primary outcome separately, no significant differences in terms of efficacy between DOACs and warfarin were observed. Turning to the safety endpoints of interest, DOACs demonstrated a more favorable profile than warfarin, resulting in fewer major bleeding, gastrointestinal, and intracranial bleeding events.

Given the paucity of data regarding the safety and effectiveness of DOACs in individuals with class III obesity (BMI ≥ 40 kg/m^2^), the International Society of Thrombosis and Hemostasis recommends exercising caution when prescribing these anticoagulants to such patients [52]. Conversely, the 2023 update from the American College of Cardiology/American Heart Association joint committee on clinical practice guidelines for the diagnosis and management of AF recommends that, for individuals with atrial fibrillation and class III obesity, choosing DOACs over warfarin is a reasonable approach for reducing the risk of stroke [53]. Contrary to these recommendations, a recent study leveraging real-world data highlighted a notable increase in the prescription of DOAC across the general population over the years, juxtaposed with a distinct declining trend among obese individuals from 2018 onward [54]. However, given the emerging evidence suggesting the efficacy and safety of DOACs in the periprocedural management of this patient population [55], clearer guideline recommendations are required to potentially further facilitate their prescription to obese individuals.

Importantly, we did not observe any significant differences in the prespecified subgroup analyses. Specifically, the efficacy and safety of DOACs were independent of the indication for anticoagulation, type of study design, duration of follow-up, and age. A noteworthy finding is that DOACs were equally effective and safe in both obese (BMI ≥ 30 kg/m^2^) and morbidly obese (BMI ≥ 40 kg/m^2^) patients compared to VKAs. Additionally, meta-regression analyses indicated that studies with a higher percentage of patients with cancer reported a greater risk for the primary composite outcome and all-cause mortality. Despite the promising evidence regarding the use of DOACs in non-obese patients with cancer-related thrombosis [56], our results suggest that, given the lack of pertinent RCTs, DOACs should be avoided in this specific subpopulation when obesity is present. Similarly, DOAC-treated individuals with baseline chronic renal disease exhibited a higher risk of all-cause mortality compared to those receiving VKAs. Once again, although recent research indicates benefits of DOACs in patients with renal disease [57], the evidence remains unclear in cases involving multiple comorbidities such as obesity.

Even as we step into a new era of anticoagulation therapies, marked by the emergence of novel agents such as Abelacimab—a factor XI/XIa inhibitor—with promising efficacy and a favorable safety profile based on preliminary findings from the AZALEA-TIMI 71 trial [58], several gaps regarding the utilization of available DOACs in specific subpopulations, including the obese, remain incompletely elucidated. Indeed, the pivotal DOACs trials have shown a consistent trend in underrepresentation of individuals with obesity among their participants. For instance, in the RE-LY trial, 10% (1787) of the patients had a BMI greater than 36 kg/m^2^ [59], whereas the ROCKET-AF trial enrolled 1898 patients (13.5%) with a BMI of 35 kg/m^2^ or higher [60]. In the ENGAGE-AF TIMI 48 trial, 34.8% (7308) of patients had a BMI between 30 and 40 kg/m^2^, whereas, in the ARISTOTLE trial the corresponding percentage was 33.9% (6153) [61]. The same observation also applies to trials focusing on VTE, where the percentage of participants with a weight exceeding 100 kg falls between 15 and 20% [62,63,64]. Although the data are sparse, our findings align with the conclusions established in the aforementioned landmark studies and in recent meta-analyses involving patients with AF [65] or VTE [66], and provide a more robust examination of the efficacy and safety of DOACs in obese individuals by integrating evidence from real-world studies and expanding the analysis to include additional outcomes of interest. Moreover, consistent with our findings, secondary analyses of primary RCTs revealed non-significant differences between DOACs and warfarin in terms of separate efficacy endpoints within the higher BMI categories [60,67]. Further supporting our results, a recent meta-analysis of four RCTs (58,464 patients) [68] showed that the treatment effect of DOACs compared to warfarin in atrial fibrillation is generally consistent for stroke and systemic embolic events across the spectrum of BMI. However, the reduction in major bleeding is less pronounced in individuals with higher BMI or BW. While NOACs overall reduce mortality and improve net clinical outcomes compared to warfarin, uncertainties remain for these outcomes in patients with very high BMI.

Several studies have focused on DOACs pharmacokinetics and pharmacodynamics in patients with obesity, contributing valuable mechanistic insights. In a subgroup analysis of the RE-LY trial, dose-normalized trough concentrations were 21% lower in the high body weight group (>100 kg) compared to the reference body weight group (50–100 kg) [69]. Similar trends were observed for peak concentrations, illustrating an inverse relationship between weight and trough or peak concentration [69]. In another retrospective study of 38 patients with a body weight exceeding 120 kg taking DOACs for any indication, peak plasma concentrations were measured; among the 10 patients on dabigatran, 20% exhibited peak concentrations below the median trough level (93 ng/mL) and the usual on-treatment range for peak levels (median 184, 10th–90th percentiles 74–383 ng/mL), as determined from pharmacokinetic studies in non-obese patients [70]. Considering rivaroxaban, in a population pharmacokinetic model derived from the combined data from two phase II studies focusing on deep vein thrombosis treatment (EINSTEIN-DVT and ODIXa-DVT trials), peak concentration was not found to be significantly influenced by body weight [71]. In contrast, a comprehensive pooled population pharmacokinetic model incorporating data from 4918 patients enrolled in seven clinical trials demonstrated that, contrary to body weight having a minor influence on rivaroxaban’s pharmacokinetics, renal function emerged as the primary determinant of rivaroxaban exposure across all indications [72].

The degree to which alterations in pharmacokinetics and pharmacodynamics manifest as changes in clinical outcomes remains uncertain. Of note, our findings suggest that DOACs exhibit comparable efficacy with warfarin when considering the individual analyzed efficacy endpoints. The absence of discernible differences in the studied outcomes, despite pharmacokinetic variances in obese patients, could be ascribed to the notion that these theoretical changes in pharmacokinetics might not be substantial enough to lead to meaningful alterations in pharmacodynamics, as supported by previous trials [69]. Moreover, the diverse pharmacokinetic changes induced by obesity might offset each other, leading to trivial changes in drug effectiveness [73].

### 4.1. Strengths and Limitations

While the current study was conducted with rigorous adherence to pertinent methodological and reporting guidelines, along with a preregistered protocol, it is crucial to acknowledge certain limitations. Firstly, there was notable between-study heterogeneity, likely stemming from variations in sample size, DOAC dosing, length of follow-up, and participants’ baseline characteristics. Secondly, most of the studies included in the analysis were retrospective cohort studies, introducing inherent limitations and potential biases. Thirdly, uncertainties persist regarding whether the observed favorable profile of DOACs in patients with obesity should be deemed a class effect, given that performing a subgroup analysis based on the various available DOACs was not feasible due to the lack of relevant data.

### 4.2. Conclusions

This extensive systematic review and meta-analysis demonstrated that the utilization of DOACs is associated with a significantly greater reduction in the combined risk of any stroke, SE, MI, or all-cause mortality in obese patients with AF or VTE compared to warfarin. Additionally, this benefit is accompanied by a notable reduction in major bleeding, gastrointestinal bleeding, and intracranial bleeding events. Future large-scale and rigorously designed trials are essential to explore the role of DOACs across different stages of obesity potentially enabling head-to-head comparisons among the commercially available options.

## Figures and Tables

**Table 1:** Analysis summary for all outcomes.

Outcome	Studies (N)	Participants (N)	RR with 95% CI	*p*-Value	Prediction Interval	I^2^ with 95% CI	Cochran’s Q *p*-Value	Egger’s Test *p*-Value
A. Efficacy outcomes								
Composite	20	319,689	0.8 (0.65, 0.98)	0.033	(0.37, 1.74)	0.95 (0.93, 0.96)	<0.001	0.742
All-cause mortality	12	48,908	0.77 (0.49, 1.2)	0.247	(0.17, 3.46)	0.97 (0.96, 0.98)	<0.001	0.633
Stroke or systemic embolism	18	331,210	0.9 (0.71, 1.15)	0.409	(0.35, 2.35)	0.94 (0.92, 0.95)	<0.001	0.562
Myocardial infarction	3	34,910	0.82 (0.51, 1.32)	0.413	(0, 185.92)	0.88 (0.65, 0.96)	<0.001	0.897
Ischemic stroke	13	317,292	0.94 (0.72, 1.22)	0.649	(0.36, 2.48)	0.93 (0.9, 0.95)	<0.001	0.454
Hemorrhagic stroke	9	305,061	0.58 (0.38, 0.88)	0.011	(0.14, 2.49)	0.92 (0.86, 0.95)	<0.001	0.136
Systemic embolism	7	268,722	0.81 (0.63, 1.05)	0.113	(0.47, 1.42)	0.26 (0, 0.68)	0.23	0.074
Venous thromboembolism	20	110,160	0.8 (0.63, 1)	0.052	(0.33, 1.89)	0.87 (0.81, 0.91)	<0.001	0.502
B. Safety outcomes								
Major bleeding	34	434,023	0.76 (0.63, 0.92)	0.004	(0.31, 1.89)	0.94 (0.92, 0.95)	<0.001	0.524
Minor bleeding	14	76,479	0.88 (0.71, 1.09)	0.257	(0.48, 1.62)	0.77 (0.62, 0.86)	<0.001	0.437
Gastrointestinal bleeding	5	181,272	0.59 (0.49, 0.72)	<0.001	(0.3, 1.16)	0.88 (0.74, 0.94)	<0.001	0.491
Intracranial bleeding	6	165,384	0.45 (0.33, 0.6)	<0.001	(0.19, 1.05)	0.52 (0, 0.82)	0.08	0.202

Abbreviations: RR, risk ratio; CI, confidence interval.

## Data Availability

The data generated in this research will be shared on reasonable request to the corresponding author.

## Supplementary Materials

The following supporting information can be downloaded at: https://www.mdpi.com/article/10.3390/jcm13133784/s1, Table S1. Checklist of reported items of the PRISMA 2020 statement; Table S2. Full search strategy for MEDLINE; Table S3. Characteristics of included studies; Table S4. Assessments based on the Newcastle-Ottawa Scale; Figure S1. Preferred Reporting Items for Systematic reviews and Meta-Analyses (PRISMA) flowchart; Figure S2. Forest plot of the outcome of any stroke or systemic embolism; Figure S3. Forest plot of the outcome of myocardial infarction; Figure S4. Forest plot of the outcome of ischemic stroke; Figure S5. Forest plot of the outcome of hemorrhagic stroke; Figure S6. Forest plot of the outcome of systemic embolism; Figure S7. Forest plot of the outcome of venous thromboembolism; Figure S8. Funnel plots of the efficacy outcomes; Figure S9. Forest plot of the outcome of minor bleeding; Figure S10. Forest plot of the outcome of gastrointestinal bleeding; Figure S11. Forest plot of the outcome of intracranial bleeding.
