# Supplementary material for: Efficacy and Safety of Direct Oral Anticoagulants versus Warfarin in Obese Patients (BMI ≥ 30 kg/m2) with Atrial Fibrillation or Venous Thromboembolism: An Updated Systematic Review and Meta-Analysis
Source: J Clin Med. 2024 Jun 27;13(13):3784. doi: 10.3390/jcm13133784 (PMC11242099; doi:10.3390/jcm13133784)
Supplement: Supplementary file 1 [file jcm-13-03784-s001.zip › jcm-3059027-supplementary.pdf]

Supplemental Material

of

Efficacy and Safety of Direct Oral Anticoagulants versus Warfarin in Obese Patients (BMI≥30kg/m2) with Atrial Fibrillation or Venous Thromboembolism: an updated Systematic Review and Meta-analysis

Table of Contents

Table S1. Checklist of reported items of the PRISMA 2020 statement 6

Table S2. Full search strategy for MEDLINE 8

Table S3. Characteristics of included studies 9

Table S4. Assessments based on the Newcastle-Ottawa Scale 12

Figure S1. Preferred Reporting Items for Systematic reviews and Meta-Analyses (PRISMA) flowchart 15

Figure S2. Forest plot of the outcome of any stroke or systemic embolism 16

Figure S3. Forest plot of the outcome of myocardial infarction 16

Figure S4. Forest plot of the outcome of ischemic stroke 17

Figure S5. Forest plot of the outcome of hemorrhagic stroke 17

Figure S6. Forest plot of the outcome of systemic embolism 18

Figure S7. Forest plot of the outcome of venous thromboembolism 18

Figure S8. Funnel plots of the efficacy outcomes 19

Figure S9. Forest plot of the outcome of minor bleeding 19

Figure S10. Forest plot of the outcome of gastrointestinal bleeding 20

Figure S11. Forest plot of the outcome of intracranial bleeding 20

Figure S12. Funnel plots of the safety outcomes 22

Figure S13. Subgroup analysis based on anticoagulation indication for the composite primary efficacy outcome 22

Figure S14. Subgroup analysis based on anticoagulation indication for the outcome of all-cause mortality 23

Figure S15. Subgroup analysis based on anticoagulation indication for the outcome of any stroke or systemic embolism 23

Figure S16. Subgroup analysis based on anticoagulation indication for the outcome of myocardial infarction 24

Figure S17. Subgroup analysis based on anticoagulation indication for the outcome of ischemic stroke 24

Figure S18. Subgroup analysis based on anticoagulation indication for the outcome of hemorrhagic stroke 25

Figure S19. Subgroup analysis based on anticoagulation indication for the outcome of systemic embolism 25

Figure S20. Subgroup analysis based on anticoagulation indication for the outcome of venous thromboembolism 25

Figure S21. Subgroup analysis based on anticoagulation indication for the outcome of major bleeding 26

Figure S22. Subgroup analysis based on anticoagulation indication for the outcome of minor bleeding 27

Figure S23. Subgroup analysis based on anticoagulation indication for the outcome of gastrointestinal bleeding 27

Figure S24. Subgroup analysis based on anticoagulation indication for the outcome of intracranial bleeding 28

Figure S25. Subgroup analysis based on study type for the composite primary efficacy outcome 28

Figure S26. Subgroup analysis based on study type for the outcome of all-cause mortality 29

Figure S27. Subgroup analysis based on study type for the outcome of any stroke or systemic embolism 29

Figure S28. Subgroup analysis based on study type for the outcome of venous thromboembolism 30

Figure S29. Subgroup analysis based on study type for the outcome of major bleeding .. 31

Figure S30. Subgroup analysis based on study type for the outcome of minor bleeding .. 32

Figure S31. Subgroup analysis based on minimum body mass index (BMI) as inclusion criterion for the composite primary efficacy outcome 32

Figure S32. Subgroup analysis based on minimum body mass index (BMI) as inclusion criterion for the outcome of all-cause mortality 33

Figure S33. Subgroup analysis based on minimum body mass index (BMI) as inclusion criterion for the outcome of any stroke or systemic embolism 33

Figure S34. Subgroup analysis based on minimum body mass index (BMI) as inclusion criterion for the outcome of myocardial infarction 33

Figure S35. Subgroup analysis based on minimum body mass index (BMI) as inclusion criterion for the outcome of ischemic stroke 34

Figure S36. Subgroup analysis based on minimum body mass index (BMI) as inclusion criterion for the outcome of hemorrhagic stroke 34

Figure S37. Subgroup analysis based on minimum body mass index (BMI) as inclusion criterion for the outcome of venous thromboembolism 35

Figure S38. Subgroup analysis based on minimum body mass index (BMI) as inclusion criterion for the outcome of major bleeding 36

Figure S39. Subgroup analysis based on minimum body mass index (BMI) as inclusion criterion for the outcome of minor bleeding 37

Figure S40. Subgroup analysis based on minimum body mass index (BMI) as inclusion criterion for the outcome of gastrointestinal bleeding 37

Figure S41. Subgroup analysis based on follow up duration for the composite primary efficacy outcome 38

Figure S42. Subgroup analysis based on follow up duration for the outcome of all-cause mortality 38

Figure S43. Subgroup analysis based on follow up duration for the outcome of any stroke or systemic embolism 39

Figure S44. Subgroup analysis based on follow up duration for the outcome of myocardial infarction 39

Figure S45. Subgroup analysis based on follow up duration for the outcome of ischemic stroke 40

Figure S46. Subgroup analysis based on follow up duration for the outcome of hemorrhagic stroke 40

Figure S47. Subgroup analysis based on follow up duration for the outcome of systemic embolism 41

Figure S48. Subgroup analysis based on follow up duration for the outcome of venous thromboembolism 41

Figure S49. Subgroup analysis based on follow up duration for the outcome of major bleeding 42

Figure S50. Subgroup analysis based on follow up duration for the outcome of minor bleeding 43

Figure S51. Subgroup analysis based on follow up duration for the outcome of gastrointestinal bleeding 43

Figure S52. Subgroup analysis based on follow up duration for the outcome of intracranial bleeding 44

Figure S53. Subgroup analysis based on study quality for the composite primary efficacy outcome 44

Figure S54. Subgroup analysis based on study quality for the outcome of all-cause mortality 45

Figure S55. Subgroup analysis based on study quality for the outcome of any stroke or systemic embolism 46

Figure S56. Subgroup analysis based on study quality for the outcome of myocardial infarction 46

Figure S57. Subgroup analysis based on study quality for the outcome of ischemic stroke 46

Figure S58. Subgroup analysis based on study quality for the outcome of venous thromboembolism 47

Figure S59. Subgroup analysis based on study quality for the outcome of major bleeding48

Figure S60. Subgroup analysis based on study quality for the outcome of minor bleeding 49

Figure S61. Subgroup analysis based on study quality for the outcome of gastrointestinal bleeding 49

Figure S62. Subgroup analysis based on study quality for the outcome of intracranial bleeding 50

Figure S63. Subgroup analysis based on age for the composite primary efficacy outcome 51

Figure S64. Subgroup analysis based on age for the outcome of all-cause mortality 51

Figure S65. Subgroup analysis based on age for the outcome of any stroke or systemic embolism 52

Figure S66. Subgroup analysis based on age for the outcome of myocardial infarction.... 52

Figure S67. Subgroup analysis based on age for the outcome of ischemic stroke 53

Figure S68. Subgroup analysis based on age for the outcome of hemorrhagic stroke 53

Figure S69. Subgroup analysis based on age for the outcome of systemic embolism 54

Figure S70. Subgroup analysis based on age for the outcome of venous thromboembolism 54

Figure S71. Subgroup analysis based on age for the outcome of major bleeding............. 55

Figure S72. Subgroup analysis based on age for the outcome of minor bleeding 56

Figure S73. Subgroup analysis based on age for the outcome of gastrointestinal bleeding 56

Figure S74. Subgroup analysis based on age for the outcome of intracranial bleeding..... 57

Figure S75. Leave-one-out sensitivity analysis for the composite primary efficacy outcome 57

Figure S76. Leave-one-out sensitivity analysis for the outcome of all-cause mortality 58

Figure S77. Leave-one-out sensitivity analysis for the outcome of any stroke or systemic embolism 58

Figure S78. Leave-one-out sensitivity analysis for the outcome of myocardial infarction 59

Figure S79. Leave-one-out sensitivity analysis for the outcome of ischemic stroke 59

Figure S80. Leave-one-out sensitivity analysis for the outcome of hemorrhagic stroke... 59

Figure S81. Leave-one-out sensitivity analysis for the outcome of systemic embolism.... 60

Figure S82. Leave-one-out sensitivity analysis for the outcome of venous thromboembolism 60

Figure S83. Leave-one-out sensitivity analysis for the outcome of major bleeding 61

Figure S84. Leave-one-out sensitivity analysis for the outcome of minor bleeding 62

Figure S85. Leave-one-out sensitivity analysis for the outcome of gastrointestinal bleeding 62

Figure S86. Leave-one-out sensitivity analysis for the outcome of intracranial bleeding . 62

Table S5. Results of univariate meta-regression for major bleeding and the composite outcome 63

Table S1. Checklist of reported items of the PRISMA 2020 statement

Section and Topic	Item #	Checklist item	Location where item is reported
**TITLE**			
Title	1	Identify the report as a systematic review.	1
**ABSTRACT**			
Abstract	2	See the PRISMA 2020 for Abstracts checklist.	Done
**INTRODUCTION**			
Rationale	3	Describe the rationale for the review in the context of existing knowledge.	4-5
Objectives	4	Provide an explicit statement of the objective(s) or question(s) the review addresses.	5
**METHODS**			
Eligibility criteria	5	Specify the inclusion and exclusion criteria for the review and how studies were grouped for the syntheses.	6-7
Information sources	6	Specify all databases, registers, websites, organisations, reference lists and other sources searched or consulted to identify studies. Specify the date when each source was last searched or consulted.	5
Search strategy	7	Present the full search strategies for all databases, registers and websites, including any filters and limits used.	Table S2
Selection process	8	Specify the methods used to decide whether a study met the inclusion criteria of the review, including how many reviewers screened each record and each report retrieved, whether they worked independently, and if applicable, details of automation tools used in the process.	7
Data collection process	9	Specify the methods used to collect data from reports, including how many reviewers collected data from each report, whether they worked independently, any processes for obtaining or confirming data from study investigators, and if applicable, details of automation tools used in the process.	7
Data items	10a	List and define all outcomes for which data were sought. Specify whether all results that were compatible with each outcome domain in each study were sought (e.g. for all measures, time points, analyses), and if not, the methods used to decide which results to collect.	6
	10b	List and define all other variables for which data were sought (e.g. participant and intervention characteristics, funding sources). Describe any assumptions made about any missing or unclear information.	6
Study risk of bias assessment	11	Specify the methods used to assess risk of bias in the included studies, including details of the tool(s) used, how many reviewers assessed each study and whether they worked independently, and if applicable, details of automation tools used in the process.	7-8
Effect measures	12	Specify for each outcome the effect measure(s) (e.g. risk ratio, mean difference) used in the synthesis or presentation of results.	8
Synthesis methods	13a	Describe the processes used to decide which studies were eligible for each synthesis (e.g. tabulating the study intervention characteristics and comparing against the planned groups for each synthesis (item #5)).	8
	13b	Describe any methods required to prepare the data for presentation or synthesis, such as handling of missing summary statistics, or data conversions.	8
	13c	Describe any methods used to tabulate or visually display results of individual studies and syntheses.	8
	13d	Describe any methods used to synthesize results and provide a rationale for the choice(s). If meta-analysis was performed, describe the model(s), method(s) to identify the presence and extent of statistical heterogeneity, and software package(s) used.	8
	13e	Describe any methods used to explore possible causes of heterogeneity among study results (e.g. subgroup analysis, meta-regression).	8
	13f	Describe any sensitivity analyses conducted to assess robustness of the synthesized results.	8
Reporting bias assessment	14	Describe any methods used to assess risk of bias due to missing results in a synthesis (arising from reporting biases).	8

Certainty assessment	15	Describe any methods used to assess certainty (or confidence) in the body of evidence for an outcome.	Not applicable
**Section and Topic**	**Item #**	**Checklist item**	**Location where item is reported**
**RESULTS**			
Study selection	16a	Describe the results of the search and selection process, from the number of records identified in the search to the number of studies included in the review, ideally using a flow diagram.	Figure S1
	16b	Cite studies that might appear to meet the inclusion criteria, but which were excluded, and explain why they were excluded.	Figure S1
Study characteristics	17	Cite each included study and present its characteristics.	9 and Table 1
Risk of bias in studies	18	Present assessments of risk of bias for each included study.	Table S4
Results of individual studies	19	For all outcomes, present, for each study: (a) summary statistics for each group (where appropriate) and (b) an effect estimate and its precision (e.g. confidence/credible interval), ideally using structured tables or plots.	Figures 1-3
Results of syntheses	20a	For each synthesis, briefly summarise the characteristics and risk of bias among contributing studies.	9-11
	20b	Present results of all statistical syntheses conducted. If meta-analysis was done, present for each the summary estimate and its precision (e.g. confidence/credible interval) and measures of statistical heterogeneity. If comparing groups, describe the direction of the effect.	9-11
	20c	Present results of all investigations of possible causes of heterogeneity among study results.	11, Table 2
	20d	Present results of all sensitivity analyses conducted to assess the robustness of the synthesized results.	8-9
Reporting biases	21	Present assessments of risk of bias due to missing results (arising from reporting biases) for each synthesis assessed.	9-11 and Figures S8 and S12
Certainty of evidence	22	Present assessments of certainty (or confidence) in the body of evidence for each outcome assessed.	Not applicable
**DISCUSSION**			
Discussion	23a	Provide a general interpretation of the results in the context of other evidence.	12-13
	23b	Discuss any limitations of the evidence included in the review.	13
	23c	Discuss any limitations of the review processes used.	13
	23d	Discuss implications of the results for practice, policy, and future research.	12-13
**OTHER INFORMATION**			
Registration and protocol	24a	Provide registration information for the review, including register name and registration number, or state that the review was not registered.	5
	24b	Indicate where the review protocol can be accessed, or state that a protocol was not prepared.	5
	24c	Describe and explain any amendments to information provided at registration or in the protocol.	No amendments were made
Support	25	Describe sources of financial or non-financial support for the review, and the role of the funders or sponsors in the review.	15
Competing interests	26	Declare any competing interests of review authors.	15
Availability of data, code and other materials	27	Report which of the following are publicly available and where they can be found: template data collection forms; data extracted from included studies; data used for all analyses; analytic code; any other materials used in the review.	15

From: Page MJ, McKenzie JE, Bossuyt PM, Boutron I, Hoffmann TC, Mulrow CD, et al. The PRISMA 2020 statement: an updated guideline for reporting systematic reviews. BMJ 2021;372:n71. doi: 10.1136/bmj.n71
For more information, visit: http://www.prisma-statement.org/

Table S2. Full search strategy for MEDLINE database (via Pubmed)

("Overweight"[Mesh] OR "Overweight*"[Text Word] OR "Body Mass Index"[Mesh] OR "Body Mass Index"[Text Word] OR "bmi"[Text Word] OR "Index Body Mass"[Text Word] OR "Quetelet Index"[Text Word] OR "Index Quetelet"[Text Word] OR "Quetelet's Index"[Text Word] OR "Quetelets Index"[Text Word] OR "Obesity"[Mesh] OR "obesit*"[Text Word] OR "obes*"[Text Word] OR "Obesity, Morbid"[Mesh] OR "Morbid obes*"[Text Word] OR "Morbidly obes*"[Text Word] OR "Obesities Morbid"[Text Word] OR "Obesity Severe"[Text Word] OR "Obesities Severe"[Text Word] OR "Severe Obesit*"[Text Word] OR "bmi above thirty"[Text Word] OR "bmi above 30"[Text Word] OR "bmi over thirty"[Text Word] OR "bmi over 30"[Text Word] OR "bmi above or equal thirty"[Text Word] OR "bmi above or equal 30"[Text Word] OR "bmi over or equal thirty"[Text Word] OR "bmi over or equal 30"[Text Word] OR "bmi above or equal to thirty"[Text Word] OR "bmi above or equal to 30"[Text Word] OR "bmi over or equal to thirty"[Text Word] OR "bmi over or equal to 30"[Text Word] OR "bmi greater than or equal thirty"[Text Word] OR "bmi greater than or equal 30"[Text Word] OR "bmi greater than or equal to thirty"[Text Word] OR "bmi greater than or equal to 30"[Text Word] OR "bmi>30"[Text Word] OR "bmi > 30"[Text Word] OR "bmi≥30"[Text Word] OR "bmi ≥ 30"[Text Word] OR "body mass index above thirty"[Text Word] OR "body mass index above 30"[Text Word] OR "body mass index over thirty"[Text Word] OR "body mass index over 30"[Text Word] OR "body mass index>30"[Text Word] OR "body mass index > 30"[Text Word] OR "body mass index≥30"[Text Word] OR "body mass index ≥ 30"[Text Word] OR "bmi greater than thirty"[Text Word] OR "bmi greater than 30"[Text Word] OR "bmi over thirty"[Text Word] OR "bmi over 30"[Text Word])

AND

("Factor Xa Inhibitors"[Mesh] OR "Factor Xa Inhibitor*"[Text Word] OR "Factor Xa Inhibitor*"[Text Word] OR "Inhibitor Factor Xa*"[Text Word] OR "Xa Inhibitor Factor*"[Text Word] OR "Direct Factor Xa Inhibitor*"[Text Word] OR "Direct-Acting Oral Anticoagulant*"[Text Word] OR "Anticoagulants Direct-Acting Oral"[Text Word] OR "Direct Acting Oral Anticoagulant*"[Text Word] OR "Oral Anticoagulants Direct-Acting*"[Text Word] OR "Direct Factor Xa Inhibitor*"[Text Word] OR "Anticoagulant Direct-Acting Oral*"[Text Word] OR "Oral Anticoagulant Direct-Acting*"[Text Word] OR "direct oral anticoagulant*"[Text Word] OR "new oral anticoagulant*"[Text Word] OR "newer oral anticoagulant*"[Text Word] OR "novel oral anticoagulant*"[Text Word] OR "noac*"[Text Word] OR "non vitamin k antagonist*"[Text Word] OR "non vitamin-k antagonist*"[Text Word] OR "non-vitamin k antagonist*"[Text Word] OR "apixaban" [Supplementary Concept] OR "apixaban*"[Text Word] OR "Eliquis"[Text Word] OR "BMS 562247"[Text Word] OR "BMS562247"[Text Word] OR "BMS-562247-01"[Text Word] OR "BMS-562247"[Text Word] OR "Rivaroxaban"[Mesh] OR "rivaroxaban*"[Text Word] OR "Xarelto"[Text Word] OR "BAY 59-7939"[Text Word] OR "BAY 59 7939"[Text Word] OR "BAY 597939"[Text Word] OR "Dabigatran"[Mesh] OR "dabigatran*"[Text Word] OR "BIBR 1048"[Text Word] OR "Pradaxa"[Text Word] OR "Dabigatran Etexilate"[Text Word] OR "Etexilate Dabigatran"[Text Word] OR "Dabigatran Etexilate Mesylate"[Text Word] OR "Etexilate Mesylate Dabigatran"[Text Word] OR "Mesylate Dabigatran Etexilate"[Text Word] OR "edoxaban" [Supplementary Concept] OR "edoxaban"[Text Word] OR "Savaysa"[Text Word] OR "DU-176"[Text Word] OR "DU-176b"[Text Word] OR "betrixaban" [Supplementary Concept] OR "betrixaban"[Text Word] OR "BEVYXXA"[Text Word] OR "PRT054021"[Text Word])

AND

("Warfarin"[Mesh] OR "warfarin*"[Text Word] OR "Apo-Warfarin"[Text Word] OR "Aldocumar"[Text Word] OR "Gen-Warfarin"[Text Word] OR "Warfant"[Text Word] OR "Coumadin"[Text Word] OR "Marevan"[Text Word] OR "Coumadin*"[Text Word] OR "Tedicumar"[Text Word])

Table S3. Characteristics of included studies

Author-Year	Study characteristics						Participants (N)			Age (SD) in years		Males, n/N (%)		Hypertension, n/N (%)		Diabetes, n/N (%)		Dyslipidaemia, n/N (%)		Stroke, n/N (%)		Heart failure, n/N (%)		Cancer, n/N (%)		Renal disease, n/N (%)	
	Follow up (months)	Minimum BMI inclusion criterion	Indication	Study type	Country	Study period	Total	D	W	D	W	D	W	D	W	D	W	D	W	D	W	D	W	D	W	D	W
James C Coons 2020	12	≥30	VTE	Cohort	USA	01/11-10/15	1840	632	1208	55.25 (5.5)	55 (5.8)	416/632 (66%)	774/1208 (64%)	N/R	N/R	N/R	N/R	N/R	N/R	N/R	N/R	N/R	N/R	45/632 (7%)	40/1208 (3%)	36/632 (6%)	56/1208 (5%)
Eric D Peterson 2019	10,3	≥40	AF	Cohort	USA	12/11-09/16	9474	4543	4931	61.8 (10.8)	64.4 (10.8)	2497/4543 (55%)	2605/4931 (53%)	3962/4543 (87%)	4348/4931 (88%)	2168/4543 (48%)	2841/4931 (58%)	2776/4543 (61%)	3107/4931 (63%)	N/R	N/R	1397/4543 (31%)	2218/4931 (45%)	N/R	N/R	543/4543 (12%)	1272/4931 (26%)
Margarita Kushnir 2019	9,5	≥40	AF or VTE	Cohort	USA	03/13-03/17	795	476	319	58.5 (14.1)	62.24 (15)	188/476 (39%)	111/319 (35%)	N/R	N/R	N/R	N/R	N/R	N/R	N/R	N/R	N/R	N/R	N/R	N/R	N/R	N/R
Alex C Spyropoulos 2019	10,3	≥40	VTE	Cohort	USA	2011-2016	7342	3035	4307	53.2 (12.7)	54.6 (13.4)	1232/3035 (41%)	1613/4307 (37%)	1906/3035 (63%)	2803/4307 (65%)	962/3035 (32%)	1523/4307 (35%)	1246/3035 (41%)	1876/4307 (44%)	N/R	N/R	210/3035 (7%)	398/4307 (9%)	235/3035 (8%)	338/4307 (8%)	184/3035 (6%)	448/4307 (10%)
Alexandros Briasoulis 2021	19	≥40	AF	Cohort	USA	01/10-12/18	21354	11016	10338	67.7 (1.85)	66.5 (NA)	10907/11016 (99%)	10234/10338 (99%)	9219/11016 (84%)	8973/10338 (87%)	2748/11016 (25%)	3287/10338 (32%)	N/R	N/R	623/11016 (6%)	750/10338 (7%)	3143/11016 (29%)	3701/10338 (36%)	N/R	N/R	1359/11016 (12%)	2036/10338 (20%)
Charlene Kalani 2019	N/R	≥40	AF or VTE	Cohort	USA	10/12-10/17	180	90	90	61 (NA)	63 (NA)	57/90 (63%)	48/90 (53%)	N/R	N/R	N/R	N/R	N/R	N/R	N/R	N/R	N/R	N/R	N/R	N/R	N/R	N/R
Kazuhiko Kido 2019	44	≥40	AF	Cohort	USA	01/12-12/16	128	64	64	64.28 (10.16)	65.88 (12.18)	39/64 (61%)	35/64 (55%)	N/R	N/R	N/R	N/R	N/R	N/R	12/64 (19%)	10/64 (16%)	N/R	N/R	N/R	N/R	N/R	N/R
Gregory Y H Lip 2019	2	≥30	AF	RCT	ENSURE-AF	N/R	1067	530	537	62.9 (9.3)	63.2 (10.1)	N/R	N/R	448/530 (85%)	464/537 (86%)	137/530 (26%)	139/537 (26%)	N/R	N/R	26/530 (5%)	27/537 (5%)	256/530 (48%)	245/537 (46%)	N/R	N/R	N/R	N/R
Steve Deitelzweig 2020	6,9	≥30	AF	Cohort	USA	01/13-09/15	88461	57559	30902	70.5 (10.1)	72.8 (8.8)	30707/57559 (53%)	24273/30902 (79%)	53853/57559 (94%)	29379/30902 (95%)	30332/57559 (53%)	18984/30902 (61%)	46818/57559 (81%)	25528/30902 (83%)	5967/57559 (10%)	4669/30902 (15%)	20801/57559 (36%)	14722/30902 (48%)	N/R	N/R	15654/57559 (27%)	12934/30902 (42%)
Kristina Falk 2020	N/R	≥40	AF or VTE	Cohort	USA	08/14-08/17	276	118	158	55 (12.1)	52 (13.9)	61/118 (52%)	71/158 (45%)	N/R	N/R	N/R	N/R	N/R	N/R	N/R	N/R	N/R	N/R	7/118 (6%)	7/158 (4%)	N/R	N/R
Isaac J Perales 2020	12	≥40	VTE	Cohort	USA	11/13-09/17	176	84	92	56 (14)	55 (15)	16/84 (19%)	51/92 (55%)	N/R	N/R	41/84 (49%)	48/92 (52%)	N/R	N/R	4/84 (5%)	5/92 (5%)	21/84 (25%)	23/92 (25%)	3/84 (4%)	7/92 (8%)	0/84 (0%)	10/92 (11%)

Alexander Cohen 2021 (a)	6	≥30	VTE	Cohort	USA/uk	03/14-03/19	43095	17493	25602	64.5 (14.2)	64.3 (14.3)	7167/17493 (41%)	10504/25602 (41%)	14042/17493 (80%)	20529/25602 (80%)	N/R	N/R	10048/17493 (57%)	14683/25602 (57%)	N/R	N/R	N/R	N/R	N/R	N/R	3309/17493 (19%)	4810/25602 (19%)
Amr F Barakat 2021	45,6	≥30	AF	Cohort	USA	01/1-05/18	17300	7059	6317	69.48 (10.34)	71.6 (10.4)	5024/7059 (71%)	4544/6317 (72%)	7254/7059 (103%)	6001/6317 (95%)	5285/7059 (75%)	3123/6317 (49%)	3306/7059 (47%)	4339/6317 (69%)	1187/7059 (17%)	889/6317 (14%)	2049/7059 (29%)	2453/6317 (39%)	777/7059 (11%)	568/6317 (9%)	768/7059 (11%)	569/6317 (9%)
Matthew R Weir 2021	27	≥30	AF	Cohort	USA	12/10-03/20	31078	12663	18415	68.9 (9.5)	70.8 (8.5)	7603/12663 (60%)	10655/18415 (58%)	12130/12663 (96%)	17704/18415 (96%)	N/R	N/R	10866/12663 (86%)	15681/18415 (85%)	1960/12663 (15%)	3967/18415 (22%)	4687/12663 (37%)	9420/18415 (51%)	N/R	N/R	3484/12663 (28%)	7617/18415 (41%)
Alexander T Cohen 2021 (b)	6	≥30	VTE	RCT	AMPLIFY trial	N/R	1040	522	518	54 (13.44)	53.5 (13.55)	399/522 (76%)	375/518 (72%)	293/522 (56%)	254/518 (49%)	102/522 (20%)	99/518 (19%)	145/522 (28%)	141/518 (27%)	N/R	N/R	N/R	N/R	10/522 (2%)	11/518 (2%)	N/R	N/R
Olivia S Costa 2021	12	≥30	VTE	Cohort	USA	11/11-09/18	13510	6755	6755	N/R	N/R	3127/6755 (46%)	3060/6755 (45%)	3533/6755 (52%)	3661/6755 (54%)	1445/6755 (21%)	1520/6755 (23%)	N/R	N/R	88/6755 (1%)	88/6755 (1%)	250/6755 (4%)	250/6755 (4%)	N/R	N/R	N/R	N/R
Chinthaka B Samaranayake 2021	6	≥30	VTE	Cohort	Australia	01/15-01/20	231	154	77	60.1 (NA)	59 (NA)	71/154 (46%)	36/77 (47%)	N/R	N/R	N/R	N/R	N/R	N/R	N/R	N/R	N/R	N/R	16/154 (10%)	6/77 (8%)	N/R	N/R
Jeffrey S Berger 2021 (a)	36	≥30	AF	Cohort	USA	01/10-09/19	15635	10555	5080	58.5 (8.6)	60.9 (8.8)	7335/10555 (69%)	3436/5080 (68%)	8942/10555 (85%)	4536/5080 (89%)	3958/10555 (37%)	2604/5080 (51%)	6963/10555 (66%)	3593/5080 (71%)	669/10555 (6%)	905/5080 (18%)	2940/10555 (28%)	2227/5080 (44%)	1237/10555 (12%)	N/R	3763/10555 (36%)	3314/5080 (65%)
Jeffrey S Berger 2021 (b)	36	≥30	AF	Cohort	USA	11/11-09/19	10920	7000	3920	59.5 (8.1)	61.3 (8.6)	4792/7000 (68%)	2696/3920 (69%)	6420/7000 (92%)	3636/3920 (93%)	3319/7000 (47%)	2272/3920 (58%)	5145/7000 (74%)	2951/3920 (75%)	236/7000 (3%)	347/3920 (9%)	2305/7000 (33%)	1917/3920 (49%)	932/7000 (13%)	594/3920 (15%)	3111/7000 (44%)	2913/3920 (74%)
Alexander C Perino 2021	6	≥30	VTE	Cohort	USA	10/13-09/18	24697	10619	14078	62.6 (12.4)	62.5 (12.3)	9852/10619 (93%)	13040/14078 (93%)	6493/10619 (61%)	8661/14078 (62%)	4012/10619 (38%)	5425/14078 (39%)	N/R	N/R	481/10619 (5%)	622/14078 (4%)	830/10619 (8%)	1128/14078 (8%)	N/R	N/R	2816/10619 (27%)	3788/14078 (27%)
Steve Deitelzweig 2021	N/R	≥30	AF	Cohort	USA	01/13-12/17	26522	13604	12918	75.4 (7.6)	74.4 (7.9)	13430/13604 (99%)	12739/12918 (99%)	11812/13604 (87%)	11193/12918 (87%)	6958/13604 (51%)	7248/12918 (56%)	N/R	N/R	1236/13604 (9%)	1240/12918 (10%)	4397/13604 (32%)	4824/12918 (37%)	N/R	N/R	2858/13604 (21%)	1759/12918 (14%)
Ashley Crouch 2021	12	≥40	VTE	Cohort	USA	01/12-12/19	1099	314	785	59.3 (13.9)	57.7 (14)	164/314 (52%)	345/785 (44%)	N/R	N/R	74/314 (24%)	363/785 (46%)	N/R	N/R	21/314 (7%)	32/785 (4%)	N/R	N/R	27/314 (9%)	42/785 (5%)	57/314 (18%)	147/785 (19%)
Mark J Alberts 2022	11,5	≥30	AF	Cohort	USA	12/11-03/20	95875	33191	62684	62.97 (10.3)	67.72 (10.3)	21872/33191 (66%)	39110/62684 (62%)	28370/33191 (85%)	51146/62684 (82%)	17888/33191 (54%)	44218/62684 (71%)	21418/33191 (65%)	37960/62684 (61%)	3224/33191 (10%)	11350/62684 (18%)	7410/33191 (22%)	21613/62684 (34%)	7865/33191 (24%)	19306/62684 (31%)	N/R	12277/62684 (20%)
Olivia S Costa 2020	12	≥30	AF or VTE	Cohort	USA	11/10-9/18	5304	2652	2652	N/R	N/R	1252/2652 (47%)	1223/2652 (46%)	2082/2652 (79%)	2065/2652 (78%)	1064/2652 (40%)	1102/2652 (42%)	N/R	N/R	159/2652 (6%)	190/2652 (7%)	478/2652 (18%)	557/2652 (21%)	N/R	N/R	N/R	N/R
Paige Weaver 2022	12	≥40	VTE	Cohort	USA	1/12-12/19	1272	487	785	56.6 (14.7)	57.7 (14.7)	256/487 (53%)	345/785 (44%)	N/R	N/R	172/487 (35%)	366/785 (47%)	N/R	N/R	N/R	N/R	N/R	N/R	36/487 (7%)	42/785 (5%)	56/487 (11%)	N/R
Rachel M. Watson 2022		≥35	AF or VTE	Cohort	USA	8/15-8/20	162	54	108	58 (11.7)	62 (13)	19/54 (35%)	53/108 (49%)	N/R	N/R	N/R	N/R	N/R	N/R	N/R	N/R	N/R	N/R	N/R	N/R	N/R	N/R
Christopher Bianco 2020		≥30	AF	Cohort	USA	1/12-1/18	1593	984	609	N/R	N/R	N/R	N/R	N/R	N/R	N/R	N/R	N/R	N/R	N/R	N/R	N/R	N/R	N/R	N/R	N/R	N/R

Sultan N. Alotaibi 2022	12	≥40	AF or VTE	Cohort	Saudi Arabia	3/16-3/19	250	125	125	67.68 (11.53)	60.66 (14.54)	21/125 (17%)	26/125 (21%)	N/R	N/R	N/R	N/R	N/R	N/R	19/125 (15%)	17/125 (14%)	N/R	N/R	N/R	N/R	N/R	N/R
Michael A. Lorenz 2022	12	≥50	VTE	Cohort	USA	1/05-1/20	285	80	205	65.3 (7.6)	60.1 (7.6)	78/80 (98%)	N/R	N/R	N/R	N/R	N/R	N/R	N/R	N/R	N/R	N/R	N/R	N/R	N/R	N/R	N/R
Tanvi Patil 2020	19	≥40	AF or VTE	Cohort	USA	1/15-1/18	404	214	190	69.56 (8.02)	69.34 (8.11)	207/214 (97%)	183/190 (96%)	165/214 (77%)	158/190 (83%)	116/214 (54%)	111/190 (58%)	N/R	N/R	64/214 (30%)	69/190 (36%)	54/214 (25%)	51/190 (27%)	11/214 (5%)	10/190 (5%)	N/R	N/R
Yoko M. Nakao 2022	44,4	≥30	AF	Cohort	UK	1/10-11/18	9418	2033	7385	74.83 (9.2)	74.2 (8.7)	1096/2033 (54%)	4039/7385 (55%)	1827/2033 (90%)	6482/7385 (88%)	885/2033 (44%)	2793/7385 (38%)	N/R	N/R	362/2033 (18%)	1084/7385 (15%)	437/2033 (21%)	2011/7385 (27%)	454/2033 (22%)	1445/7385 (20%)	690/2033 (34%)	2627/7385 (36%)
Hattaway Quinn 2023	12	≥40	VTE	Observational	USA	1/2015-1/2022	120	92	28	55.1 (14)	51.9 (14.8)	30/92 (33%)	15/28 (54%)	N/R	N/R	N/R	N/R	N/R	N/R	N/R	N/R	N/R	N/R	N/R	N/R	18/92 (20%)	8/28 (29%)
Karlyn A. Martin 2023	12	≥35	VTE	Observational	USA	1/2013-1/2018	5626	1868	3758	55.6 (15)	58.4 (14.6)	815/1868 (44%)	1586/3758 (42%)	N/R	N/R	N/R	N/R	N/R	N/R	N/R	N/R	N/R	N/R	498/1868 (27%)	1010/3758 (27%)	62/1868 (3%)	416/3758 (11%)
Courtney S. Pilkerton 2023	N/R	≥35	VTE	Observational	USA	1/2013-1/2018	1633	943	690	60.8 (13.4)	N/R	386/943 (41%)	N/R	N/R	N/R	N/R	N/R	N/R	N/R	N/R	N/R	N/R	N/R	N/R	N/R	N/R	N/R
Matteo Guarascio 2023	19,7	≥120 kg	AF or VTE	Observational	Italy	3/2011-06/2021	82	20	62	N/R	N/R	N/R	N/R	N/R	N/R	N/R	N/R	N/R	N/R	N/R	N/R	N/R	N/R	N/R	N/R	N/R	N/R

Abbreviations: AF,atrial fibrillation; VTE, venous thromboembolism; RCT, randomized controlled trial; USA, United States of America; D, direct oral anticoagulants; W, warfarin; SD, standard deviation; BMI, body mass index; N/R, not reported

**Table S4.** Assessments based on the Newcastle-Ottawa Scale (NOS)									
	SELECTION				COMPARABILITY	OUTCOME			
STUDY	Representativeness of the exposed cohort	Selection of the non-exposed cohort	Ascertainment of exposure	Demonstration that outcome of interest was not present at start of study	Comparability of cohorts on the basis of the design or analysis	Assessment of outcome	Was follow-up long enough for outcomes to occur	Adequacy of follow up of cohorts	Total
James C Coons 2020	1	1	1	0	2	1	1	1	**8**
Eric D Peterson 2019	1	1	1	1	2	1	1	1	**9**
Margarita Kushnir 2019	1	1	1	1	1	1	0	1	**7**
Alex C Spyropoulos 2019	1	1	1	1	1	1	1	1	**8**
Alexandros Briasoulis 2021	1	1	1	1	1	1	1	1	**8**
Charlene Kalani 2019	1	1	1	1	1	1	0	0	**6**
Kazuhiko Kido 2019	1	1	1	0	1	1	0	1	**6**
Gregory Y H Lip 2019	1	1	1	0	2	1	1	1	**7**
Steve Deitelzweig 20 20	1	1	1	1	2	1	0	1	**8**
Kristina Falk 2022	1	1	1	1	1	1	0	1	**7**
Isaac J Perales 2020	1	1	1	1	1	1	1	1	**8**
Alexander T Cohen 2021 (a)	1	1	1	1	1	1	0	0	**6**
Alexander T Cohen 2021 (b)	1	1	1	1	1	1	0	0	**6**
Amr F Barakat 2021	1	1	1	1	2	1	1	1	**9**

Table S4. Assessments based on the Newcastle-Ottawa Scale (NOS)

	SELECTION				COMPARABILITY	OUTCOME			
STUDY	Representativeness of the exposed cohort	Selection of the non-exposed cohort	Ascertainment of exposure	Demonstration that outcome of interest was not present at start of study	Comparability of cohorts on the basis of the design or analysis	Assessment of outcome	Was follow-up long enough for outcomes to occur	Adequacy of follow up of cohorts	Total
Matthew R Weir 2021	1	1	1	1	2	1	1	1	**9**
Olivia S Costa 2021	1	1	1	1	1	1	1	1	**8**
Chinthaka B Samaranayake 2021	1	1	1	1	2	1	0	1	**8**
Jeffrey S Berger 2021 (a)	1	1	1	1	2	1	1	1	**9**
Jeffrey S Berger 2021 (b)	1	1	1	1	2	1	1	1	**9**
Alexander C Perino 2021	1	1	1	1	1	1	0	1	**7**
Steve Deitelzweig 2021	1	1	1	1	1	1	1	1	**9**
Ashley Crouch 2021	1	1	1	1	2	1	1	1	**9**
Mark J Alberts 2022	1	1	1	1	2	1	1	1	**9**
Olivia S Costa 2020	1	1	1	1	2	1	1	1	**9**
Paige Weaver 2022	1	1	1	1	1	1	1	1	**8**
Rachel M. Watson 2022	1	1	1	1	0	1	1	1	**7**
Christopher Bianco 2020	1	1	1	0	1	1	0	1	**6**
Sultan N. Alotaibi 2022	1	1	1	1	1	1	0	1	**7**

**Table S4.** Assessments based on the Newcastle-Ottawa Scale (NOS)									
	SELECTION				COMPARABILITY	OUTCOME			
STUDY	Representativeness of the exposed cohort	Selection of the non-exposed cohort	Ascertainment of exposure	Demonstration that outcome of interest was not present at start of study	Comparability of cohorts on the basis of the design or analysis	Assessment of outcome	Was follow-up long enough for outcomes to occur	Adequacy of follow up of cohorts	Total
Michael A. Lorenz 2022	1	1	1	0	1	1	1	1	**7**
Tanvi Patil 2020	0	1	1	1	1	1	0	1	**6**
Yoko M. Nakao 2022	1	1	1	1	2	1	1	1	**9**
Hattaway Quinn 2023	1	1	1	1	0	1	0	0	**5**
Karlyn A. Martin 2023	1	1	1	1	2	1	1	1	**9**
Courtney S. Pilkerton 2023	1	1	1	1	2	0	1	1	**8**
Matteo Guarascio 2023	0	1	1	1	0	1	0	1	**5**

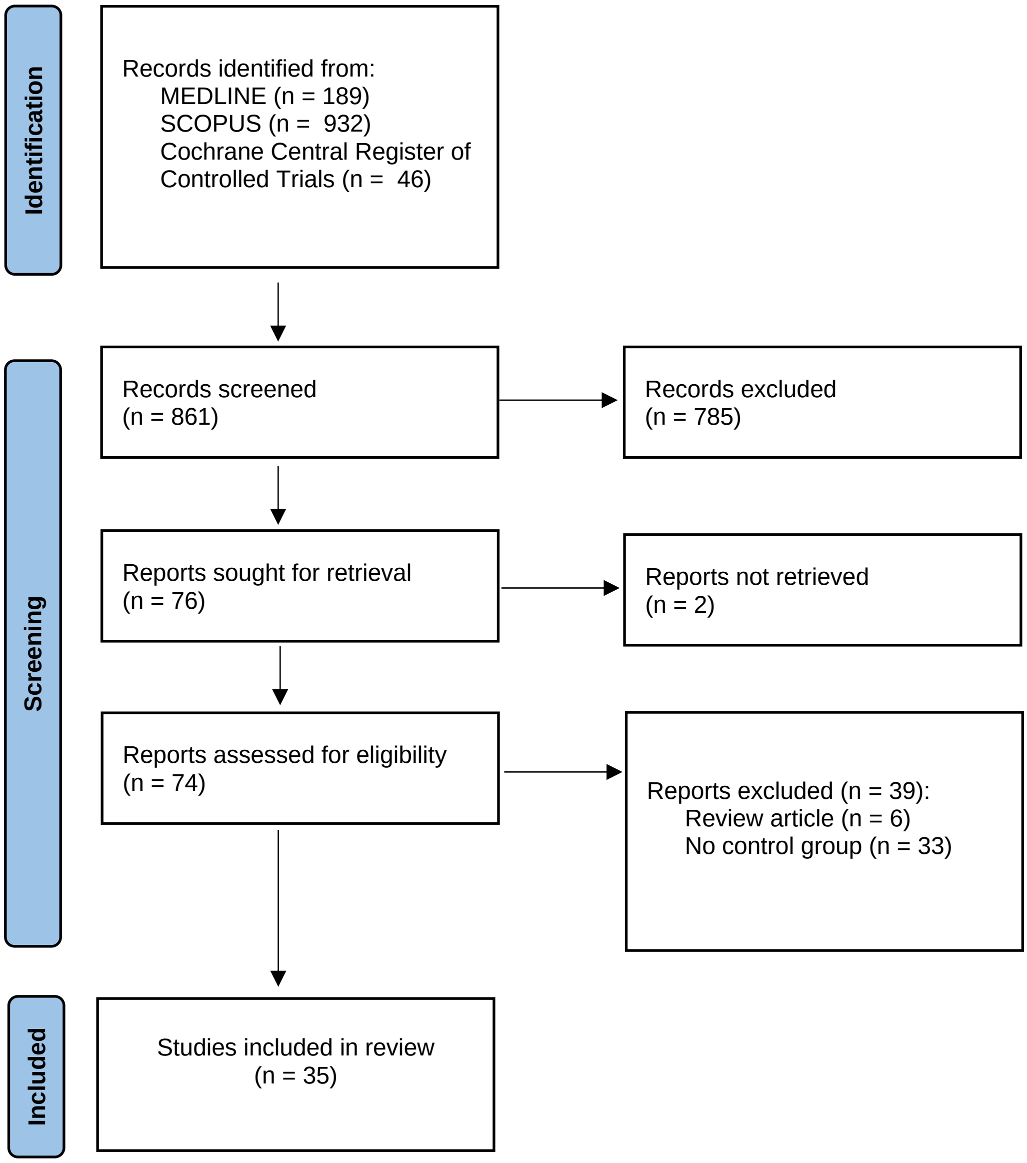

Figure S1. Preferred Reporting Items for Systematic reviews and Meta-Analyses (PRISMA) flowchart of study selection process.

Study or subgroup	DOACs Events	DOACs Total	Warfarin Events	Warfarin Total	RR	95%-CI	Weight (fixed)	Weight (random)
Steve Deitelzweig 2020	414	57559	411	30902	0.54	[0.47; 0.62]	15.9%	8.0%
Alexandros Briasoulis 2021	103	11016	177	10338	0.55	[0.43; 0.70]	5.4%	7.6%
Steve Deitelzweig 2021	148	13604	221	12918	0.64	[0.52; 0.78]	6.7%	7.8%
James C Coons 2020	0	632	1	1208	0.64	[0.03; 15.61]	0.0%	0.5%
Amr F Barakat 2021	287	7059	381	6317	0.67	[0.58; 0.78]	12.0%	8.0%
Jeffrey S Berger 2021 (b)	355	7000	279	3920	0.71	[0.61; 0.83]	10.6%	8.0%
Jeffrey S Berger 2021 (a)	258	10555	159	5080	0.78	[0.64; 0.95]	6.4%	7.8%
Gregory Y H Lip 2019	4	530	5	537	0.81	[0.22; 3.00]	0.1%	2.4%
Michael A. Lorenz 2022	0	80	1	205	0.85	[0.04; 20.67]	0.0%	0.5%
Olivia S Costa 2020	51	2652	59	2652	0.86	[0.60; 1.25]	1.8%	6.9%
Eric D Peterson 2019	52	4543	59	4931	0.96	[0.66; 1.39]	1.7%	6.9%
Kazuhiko Kido 2019	8	64	8	64	1.00	[0.40; 2.50]	0.2%	3.8%
Matthew R Weir 2021	295	12663	414	18415	1.04	[0.89; 1.20]	10.0%	8.0%
Charlene Kalani 2019	11	90	10	90	1.10	[0.49; 2.46]	0.3%	4.3%
Mark J Alberts 2022	1020	33191	1346	62684	1.43	[1.32; 1.55]	27.7%	8.2%
Sultan N. Alotaibi 2022	5	125	3	125	1.67	[0.41; 6.82]	0.1%	2.2%
Margarita Kushnir 2019	5	476	2	319	1.68	[0.33; 8.58]	0.1%	1.8%
Yoko M. Nakao 2022	51	2033	67	7385	2.77	[1.93; 3.97]	0.9%	7.0%
Kristina Falk 2020	0	118	0	158			0.0%	0.0%
Isaac J Perales 2020	0	84	0	92			0.0%	0.0%
Rachel M. Watson 2022	0	54	.	108			0.0%	0.0%
**Fixed effect model**	**3067**	**164128**	**3603**	**168448**	**0.93**	**[0.89; 0.98]**	**100.0%**	**--**
**Random effects model**					**0.90**	**[0.71; 1.15]**	**--**	**100.0%**

Heterogeneity: $I^2 = 94\%$, $\tau^2 = 0.1879$, $p < 0.01$

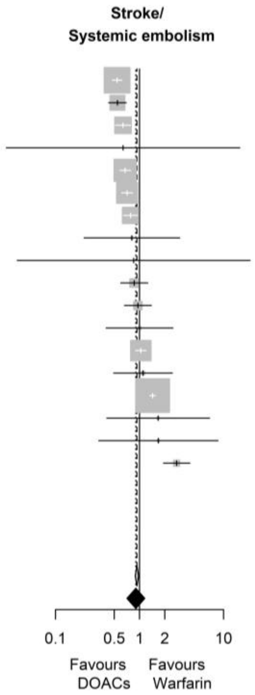

Figure S2. Forest plot of the outcome of any stroke or systemic embolism representing the comparison between direct oral anticoagulants (DOACs) and warfarin. RR, risk ratio; CI, confidence interval.

Study or subgroup	DOACs Events	DOACs Total	Warfarin Events	Warfarin Total	RR	95%-CI	Weight (fixed)	Weight (random)
Alexandros Briasoulis 2021	132	11016	208	10338	0.60	[0.48; 0.74]	37.8%	44.4%
Amr F Barakat 2021	375	7059	332	6317	1.01	[0.88; 1.17]	61.7%	46.8%
Charlene Kalani 2019	4	90	3	90	1.33	[0.31; 5.79]	0.5%	8.8%
**Fixed effect model**	**511**	**18165**	**543**	**16745**	**0.86**	**[0.76; 0.96]**	**100.0%**	**--**
**Random effects model**					**0.82**	**[0.51; 1.32]**	**--**	**100.0%**

Heterogeneity: $I^2 = 88\%$, $\tau^2 = 0.1225$, $p < 0.01$

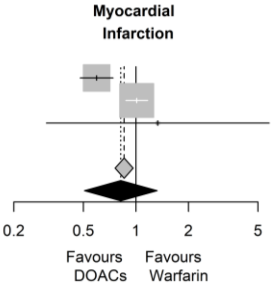

Figure S3. Forest plot of the outcome of myocardial infarction representing the comparison between direct oral anticoagulants (DOACs) and warfarin. RR, risk ratio; CI, confidence interval.

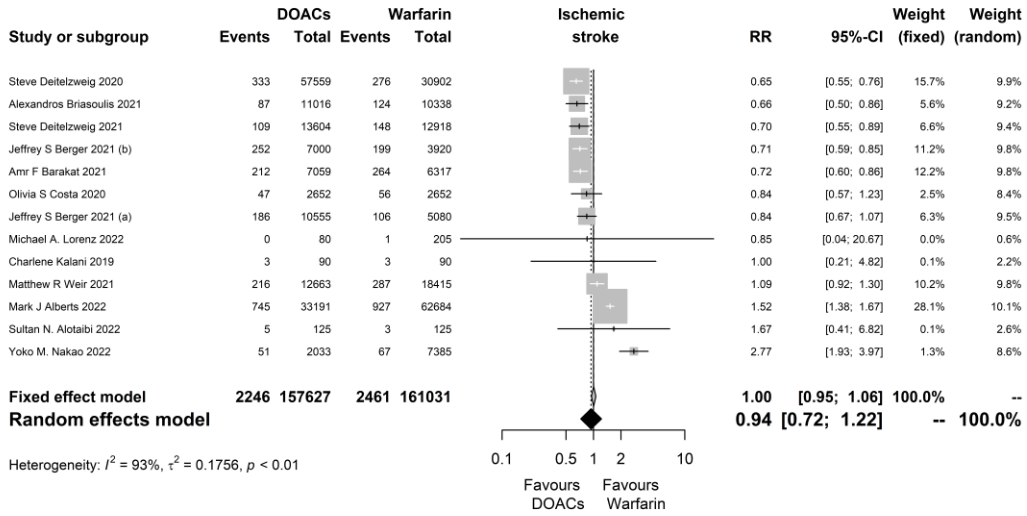

Figure S4. Forest plot of the outcome of ischemic stroke representing the comparison between direct oral anticoagulants (DOACs) and warfarin. RR, risk ratio; CI, confidence interval.

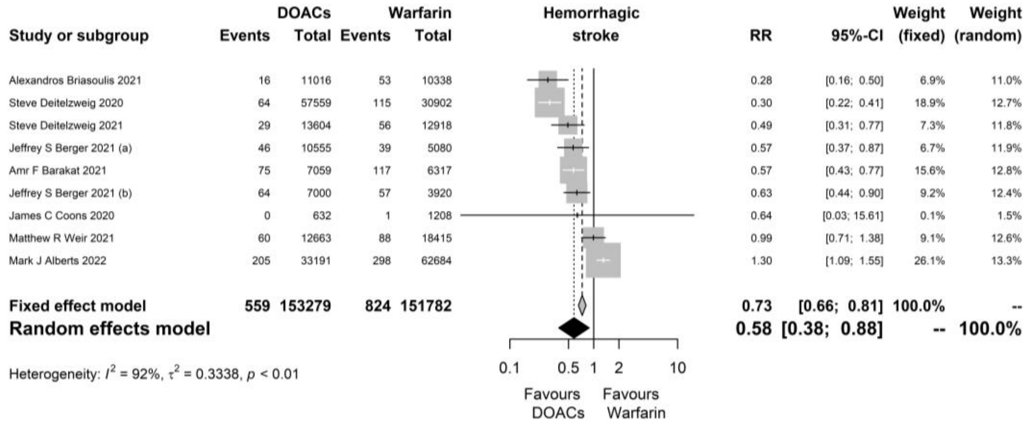

Figure S5. Forest plot of the outcome of hemorrhagic stroke representing the comparison between direct oral anticoagulants (DOACs) and warfarin. RR, risk ratio; CI, confidence interval.

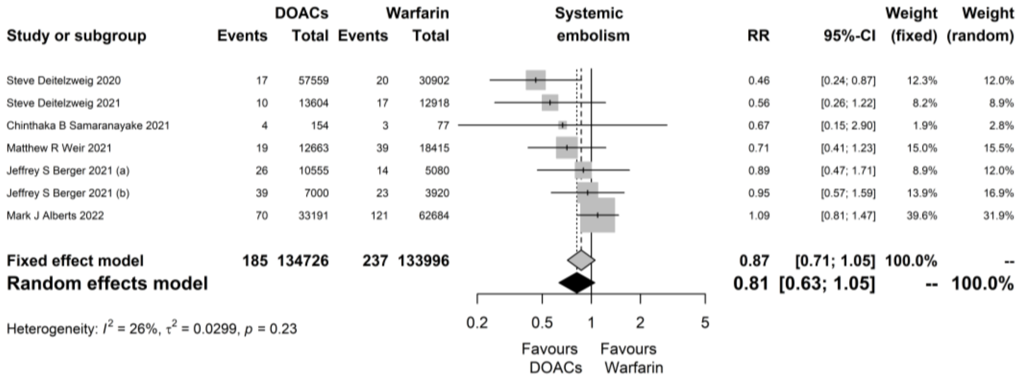

Figure S6. Forest plot of the outcome of systemic embolism representing the comparison between direct oral anticoagulants (DOACs) and warfarin. RR, risk ratio; CI, confidence interval.

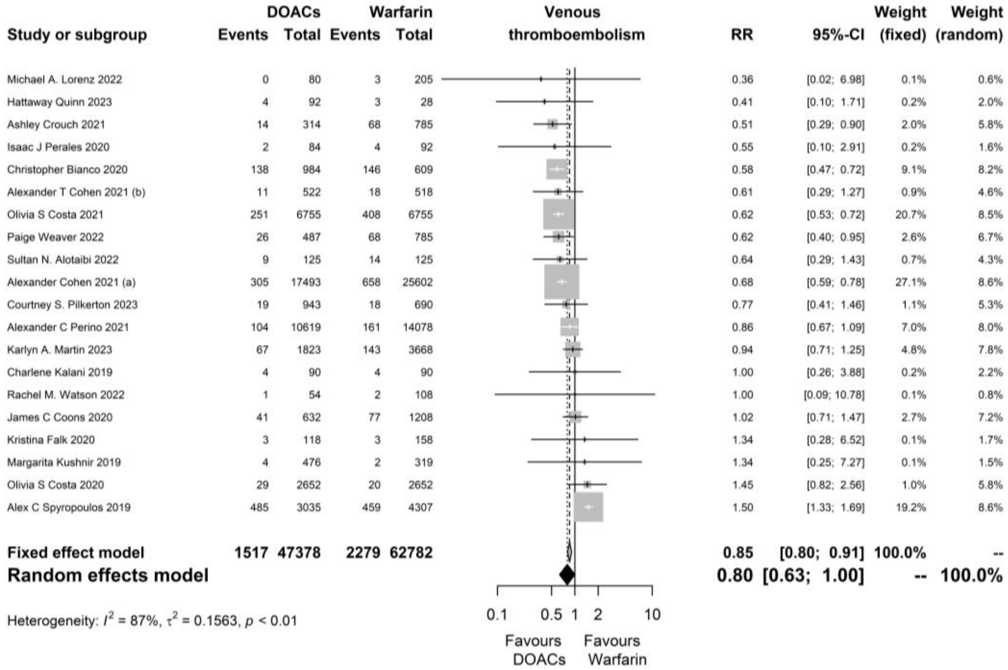

Figure S7. Forest plot of the outcome of venous thromboembolism representing the comparison between direct oral anticoagulants (DOACs) and warfarin. RR, risk ratio; CI, confidence interval.

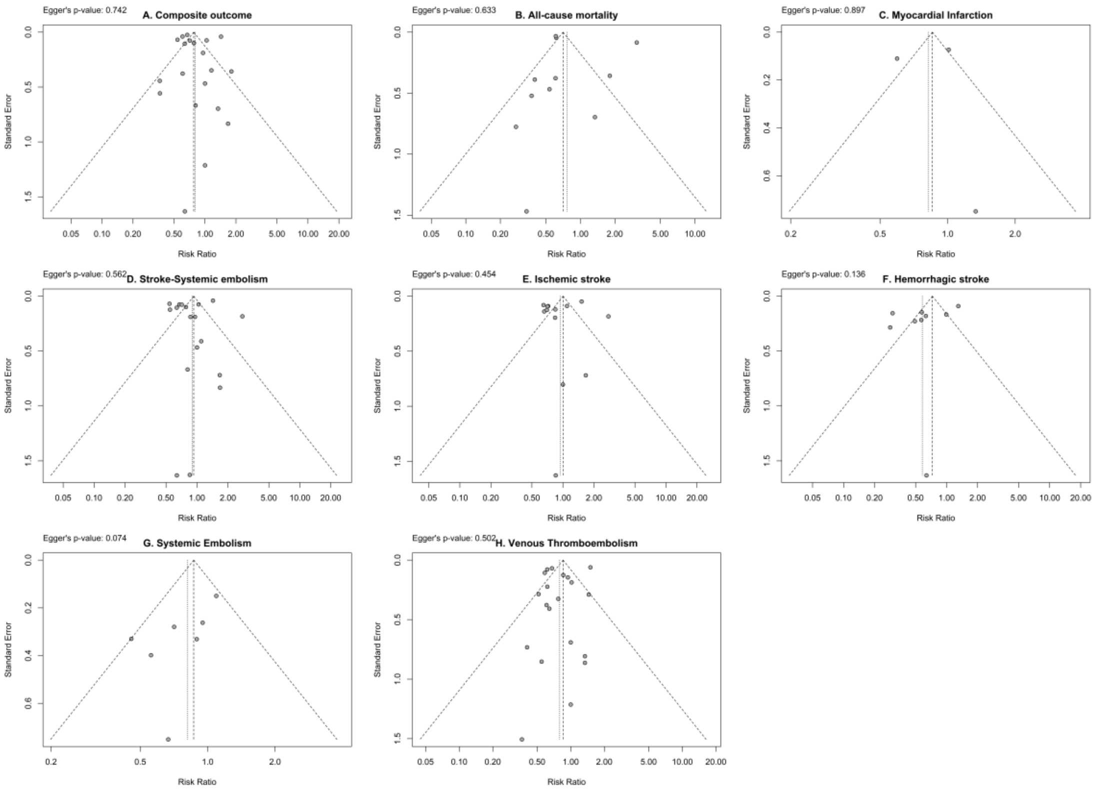

Figure S8. Funnel plots of the efficacy outcomes accompanied by the p-value of the Egger's test.

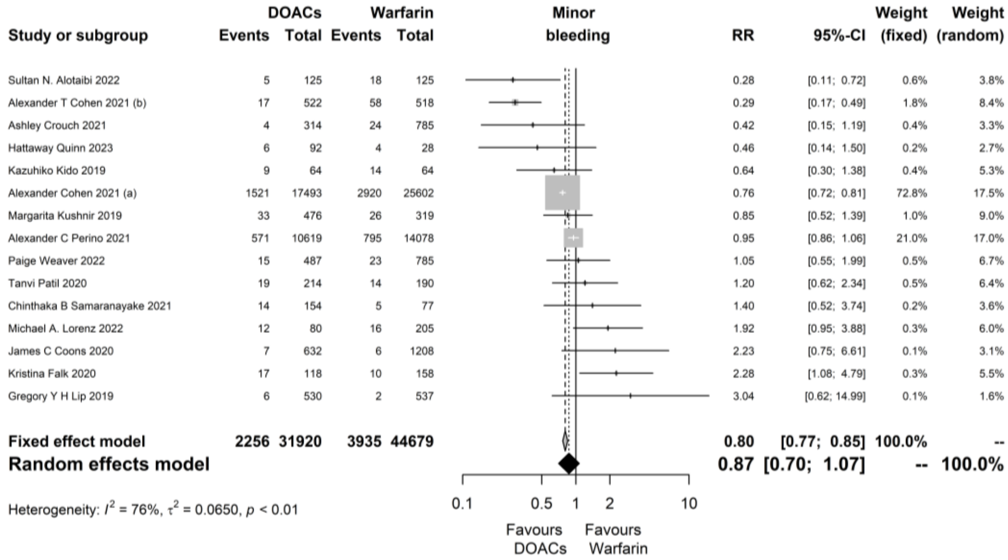

Figure S9. Forest plot of the outcome of minor bleeding representing the comparison between direct oral anticoagulants (DOACs) and warfarin. RR, risk ratio; CI, confidence interval.

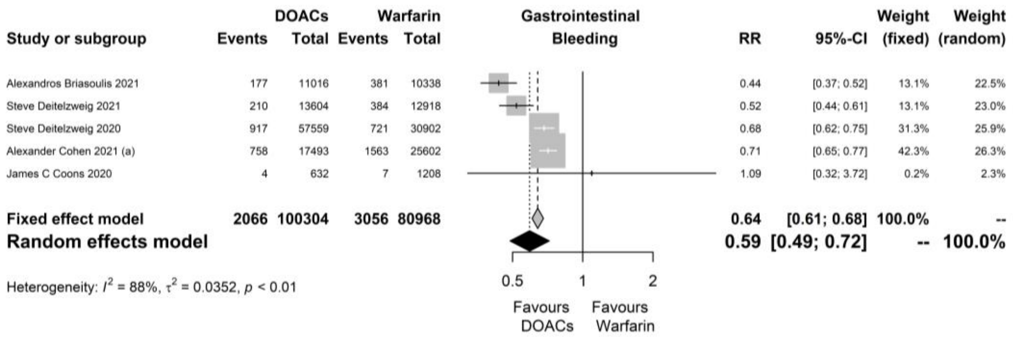

Figure S10. Forest plot of the outcome of gastrointestinal bleeding representing the comparison between direct oral anticoagulants (DOACs) and warfarin. RR, risk ratio; CI, confidence interval.

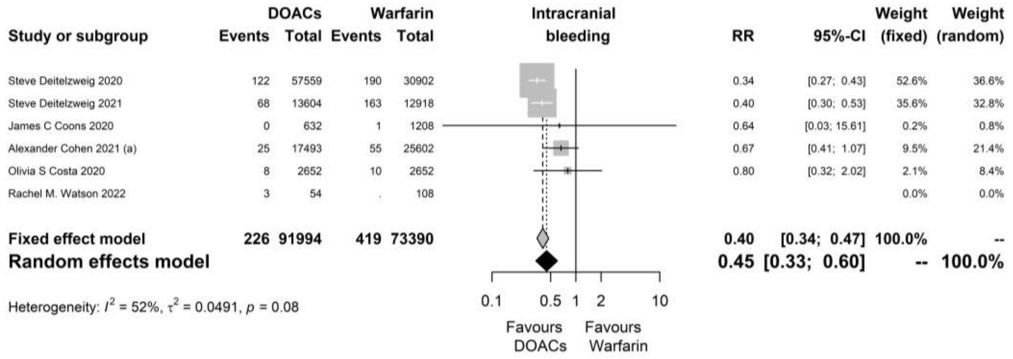

Figure S11. Forest plot of the outcome of intracranial bleeding representing the comparison between direct oral anticoagulants (DOACs) and warfarin. RR, risk ratio; CI, confidence interval.

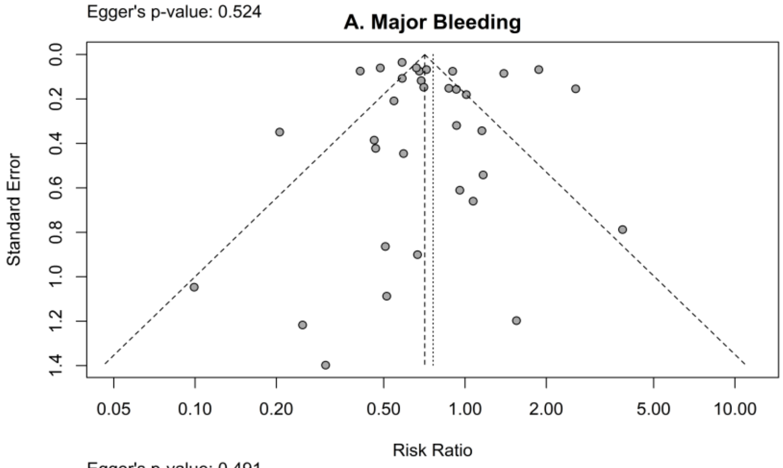
Egger's p-value: 0.524
A. Major Bleeding
Standard Error
0.0
0.2
0.4
0.6
0.8
1.0
1.2
1.4
0.05
0.10
0.20
0.50
1.00
2.00
5.00
10.00
Risk Ratio

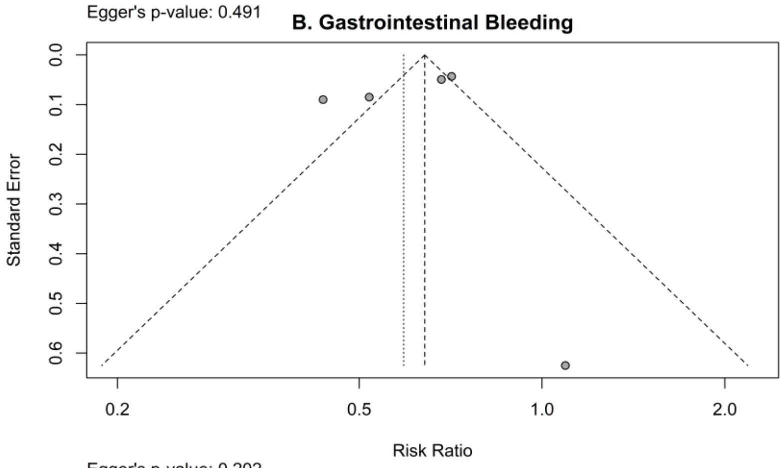
Egger's p-value: 0.491
B. Gastrointestinal Bleeding
Standard Error
0.0
0.1
0.2
0.3
0.4
0.5
0.6
0.2
0.5
1.0
2.0
Risk Ratio

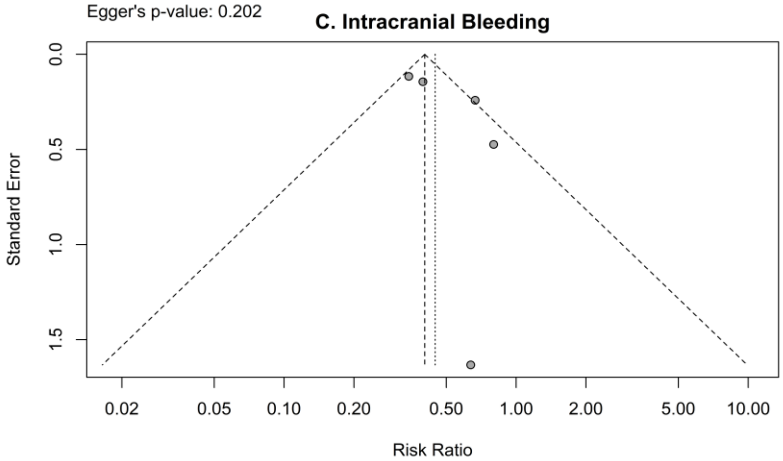
Egger's p-value: 0.202
C. Intracranial Bleeding
Standard Error
0.0
0.5
1.0
1.5
0.02
0.05
0.10
0.20
0.50
1.00
2.00
5.00
10.00
Risk Ratio

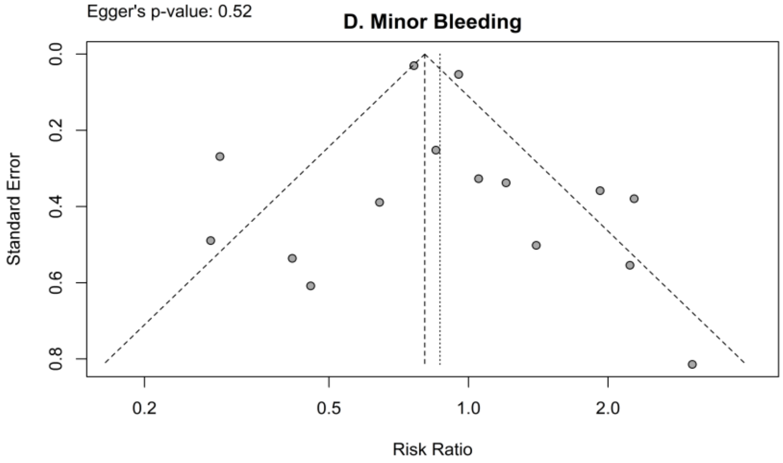
Egger's p-value: 0.52
D. Minor Bleeding
Standard Error
0.0
0.2
0.4
0.6
0.8
0.2
0.5
1.0
2.0
Risk Ratio

Figure S12. Funnel plots of the safety outcomes accompanied by the p-value of the Egger's test.

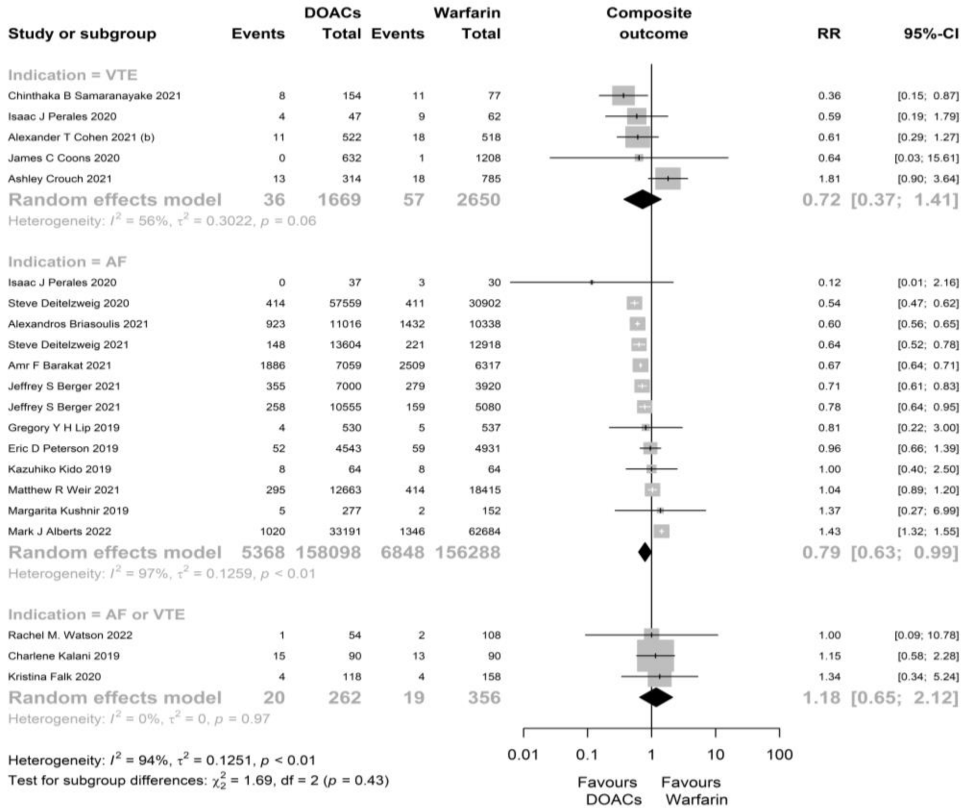

Figure S13. Subgroup analysis based on anticoagulation indication for the composite primary efficacy outcome representing the comparison between direct oral anticoagulants (DOACs) and warfarin. RR, risk ratio; CI, confidence interval.

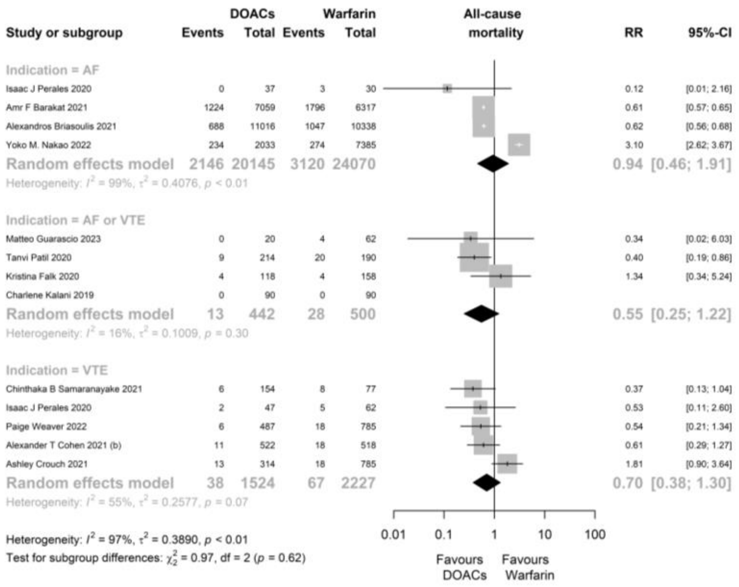

Figure S14. Subgroup analysis based on anticoagulation indication for the outcome of all-cause mortality representing the comparison between direct oral anticoagulants (DOACs) and warfarin. RR, risk ratio; CI, confidence interval.

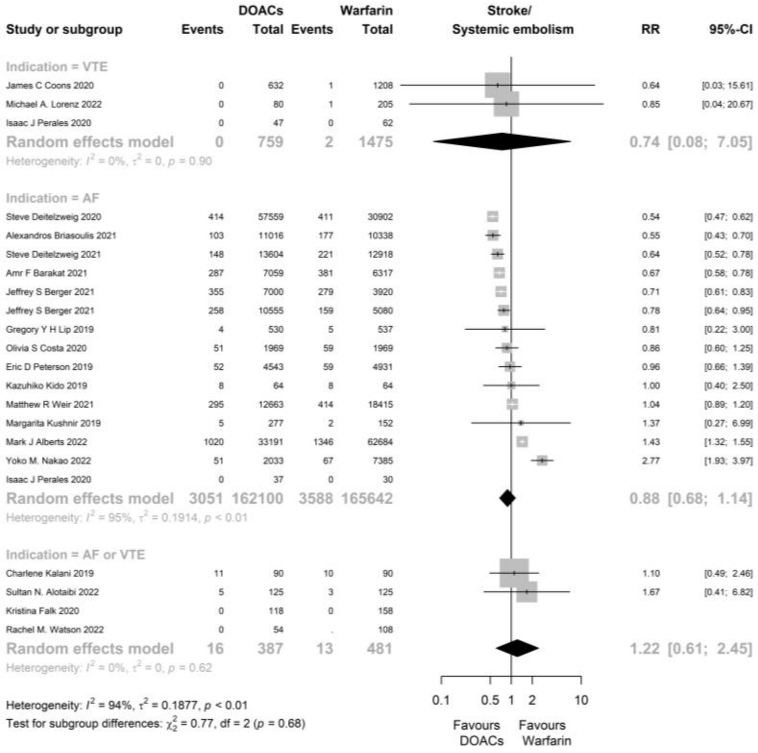

Figure S15. Subgroup analysis based on anticoagulation indication for the outcome of any stroke or systemic embolism representing the comparison between direct oral anticoagulants (DOACs) and warfarin. RR, risk ratio; CI, confidence interval.

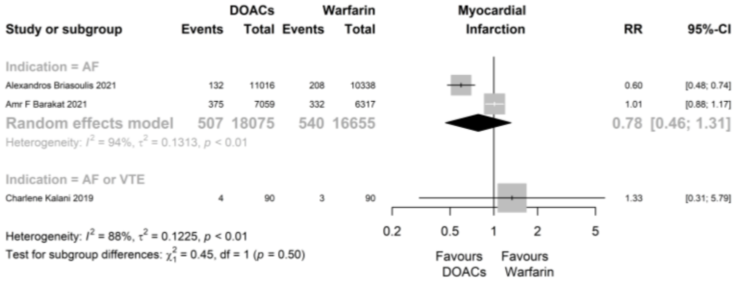

Figure S16. Subgroup analysis based on anticoagulation indication for the outcome of myocardial infarction representing the comparison between direct oral anticoagulants (DOACs) and warfarin. RR, risk ratio; CI, confidence interval.

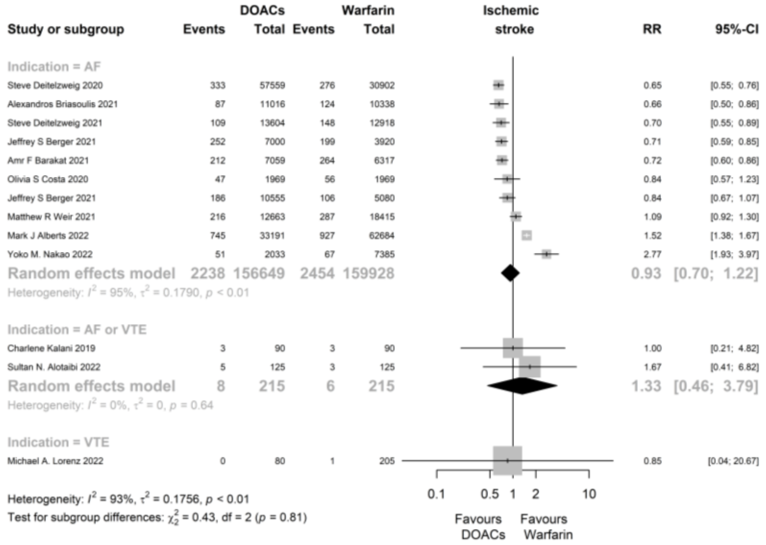

Figure S17. Subgroup analysis based on anticoagulation indication for the outcome of ischemic stroke representing the comparison between direct oral anticoagulants (DOACs) and warfarin. RR, risk ratio; CI, confidence interval.

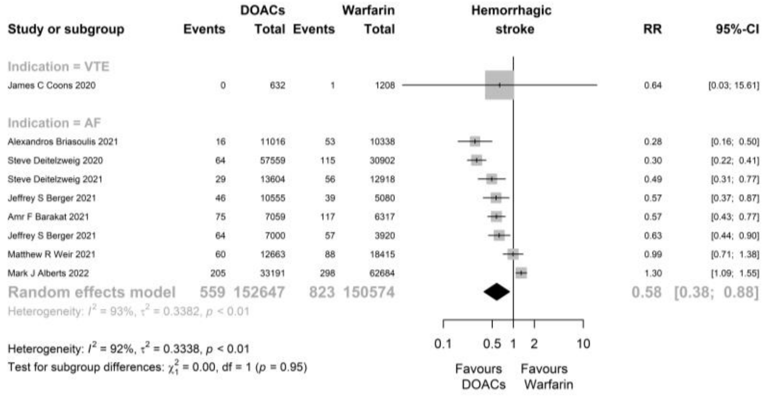

Figure S18. Subgroup analysis based on anticoagulation indication for the outcome of hemorrhagic stroke representing the comparison between direct oral anticoagulants (DOACs) and warfarin. RR, risk ratio; CI, confidence interval.

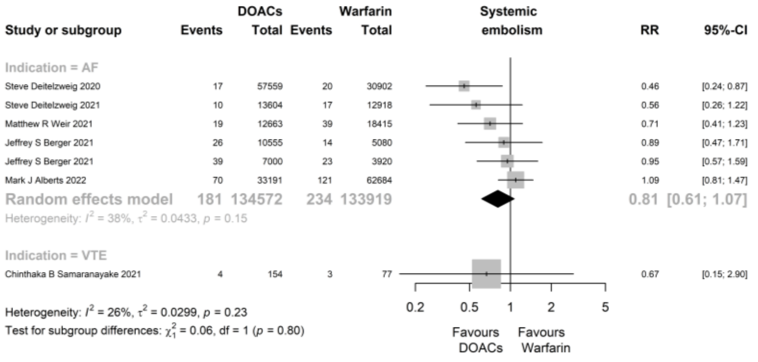

Figure S19. Subgroup analysis based on anticoagulation indication for the outcome of systemic embolism representing the comparison between direct oral anticoagulants (DOACs) and warfarin. RR, risk ratio; CI, confidence interval.

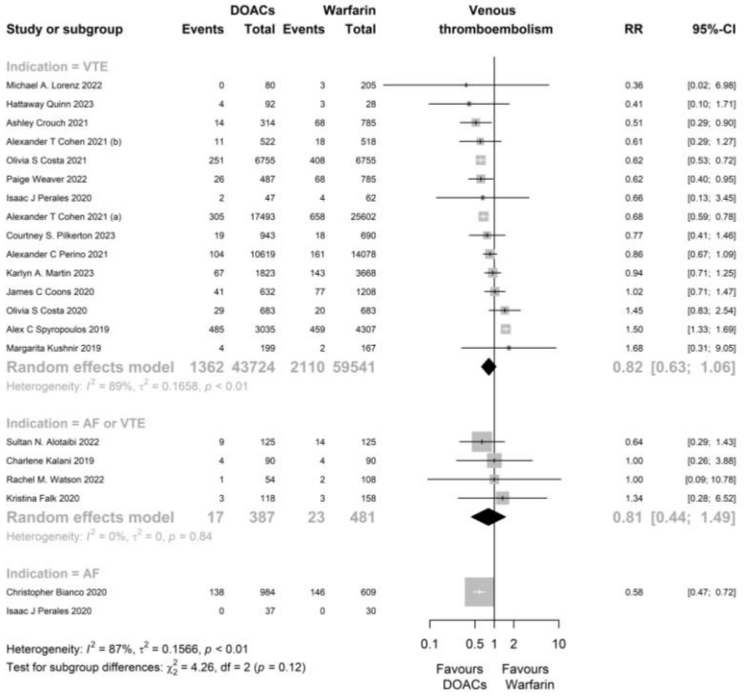

Figure S20. Subgroup analysis based on anticoagulation indication for the outcome of venous thromboembolism representing the comparison between direct oral anticoagulants (DOACs) and warfarin. RR, risk ratio; CI, confidence interval.

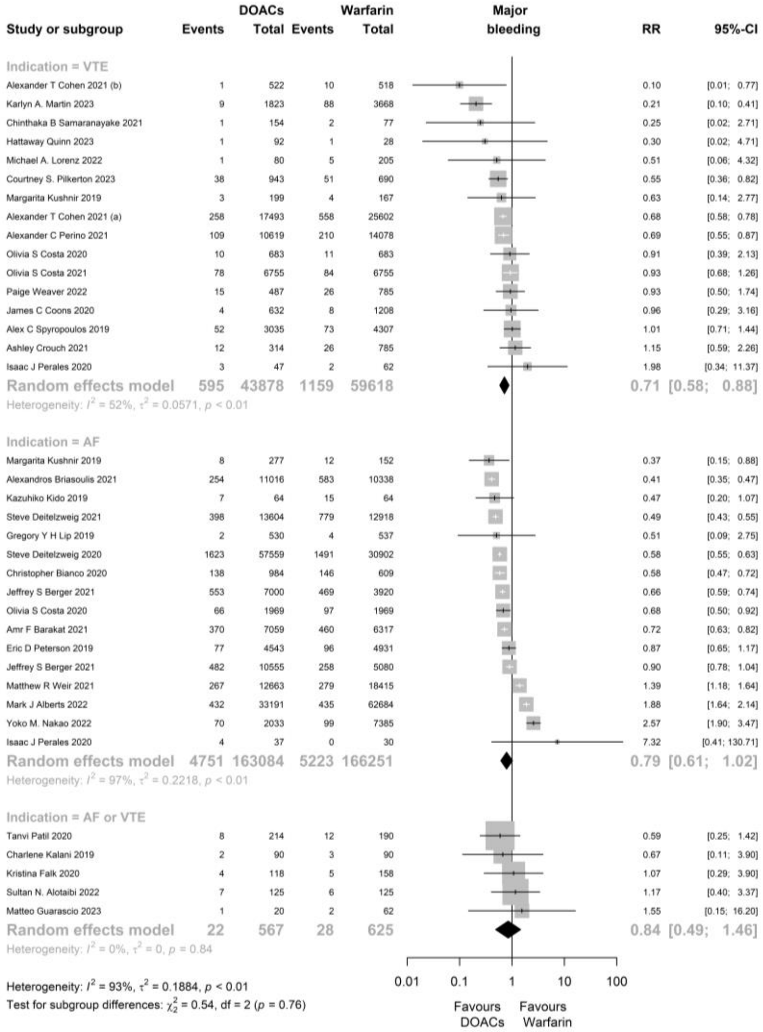

Figure S21. Subgroup analysis based on anticoagulation indication for the outcome of major bleeding representing the comparison between direct oral anticoagulants (DOACs) and warfarin. RR, risk ratio; CI, confidence interval.

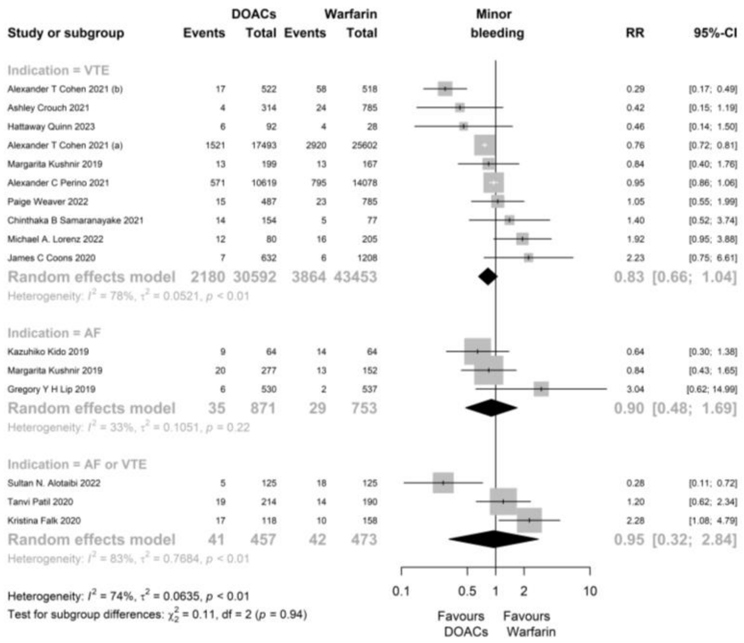

Figure S22. Subgroup analysis based on anticoagulation indication for the outcome of minor bleeding representing the comparison between direct oral anticoagulants (DOACs) and warfarin. RR, risk ratio; CI, confidence interval.

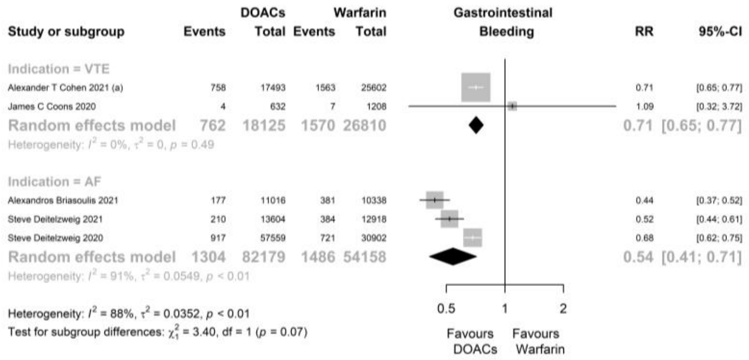

Figure S23. Subgroup analysis based on anticoagulation indication for the outcome of gastrointestinal bleeding representing the comparison between direct oral anticoagulants (DOACs) and warfarin. RR, risk ratio; CI, confidence interval.

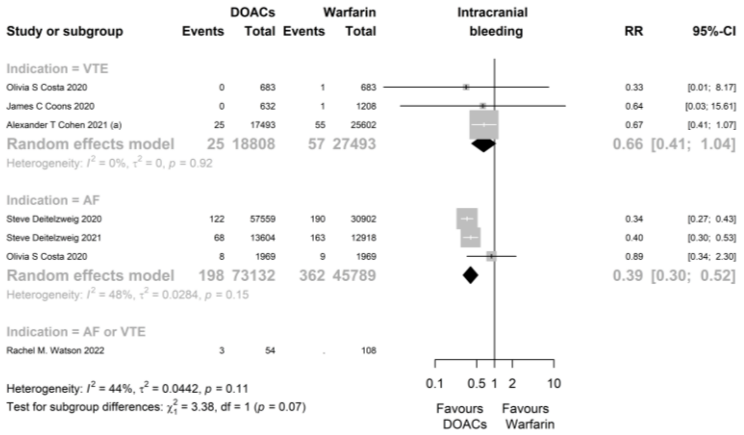

Figure S24. Subgroup analysis based on anticoagulation indication for the outcome of intracranial bleeding representing the comparison between direct oral anticoagulants (DOACs) and warfarin. RR, risk ratio; CI, confidence interval.

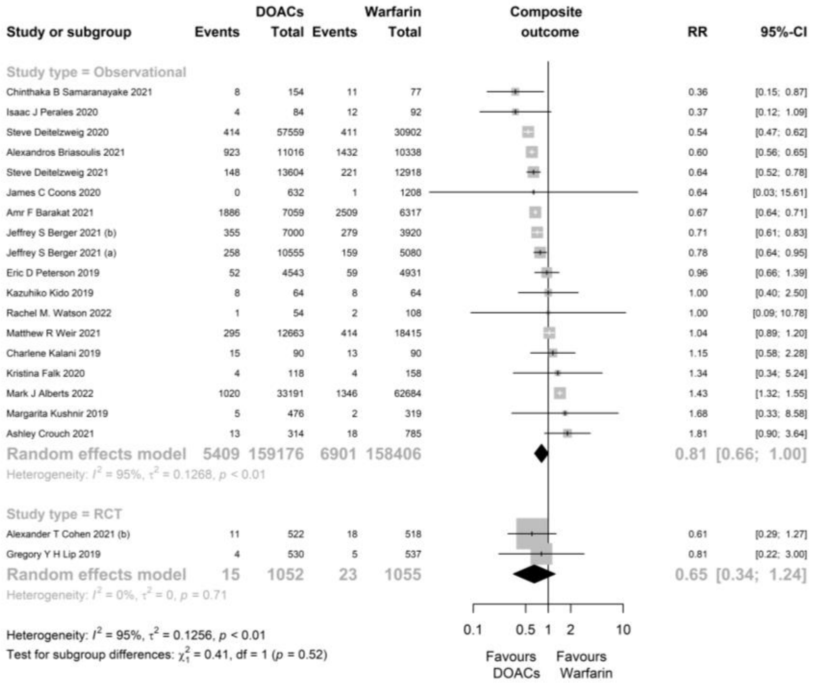

Figure S25. Subgroup analysis based on study type for the composite primary efficacy outcome representing the comparison between direct oral anticoagulants (DOACs) and warfarin. RR, risk ratio; CI, confidence interval.

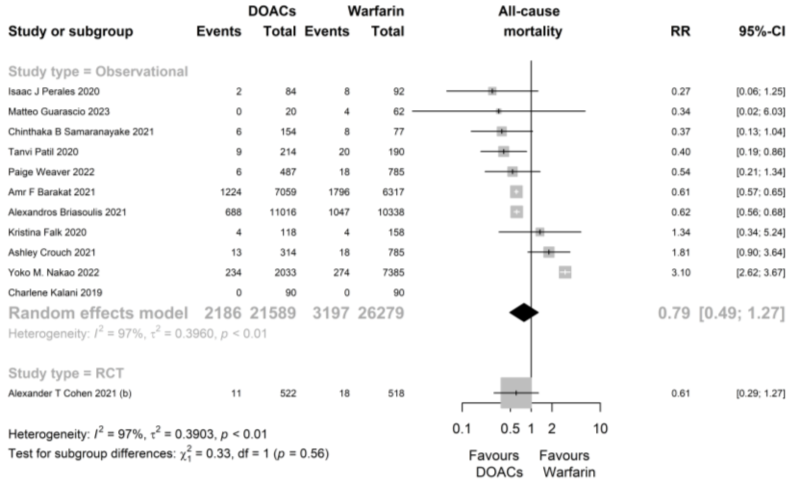

Figure S26. Subgroup analysis based on study type for the outcome of all-cause mortality representing the comparison between direct oral anticoagulants (DOACs) and warfarin. RR, risk ratio; CI, confidence interval.

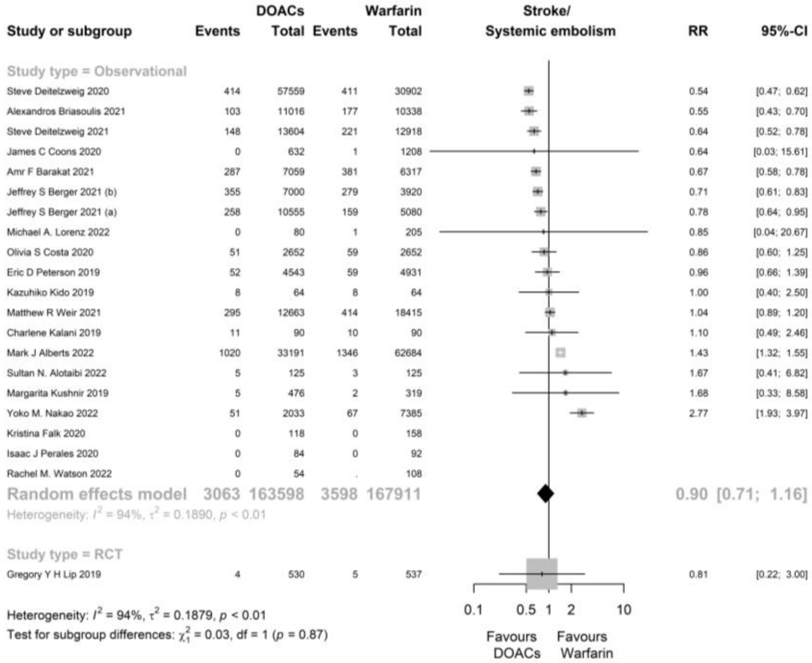

Figure S27. Subgroup analysis based on study type for the outcome of any stroke or systemic embolism representing the comparison between direct oral anticoagulants (DOACs) and warfarin. RR, risk ratio; CI, confidence interval.

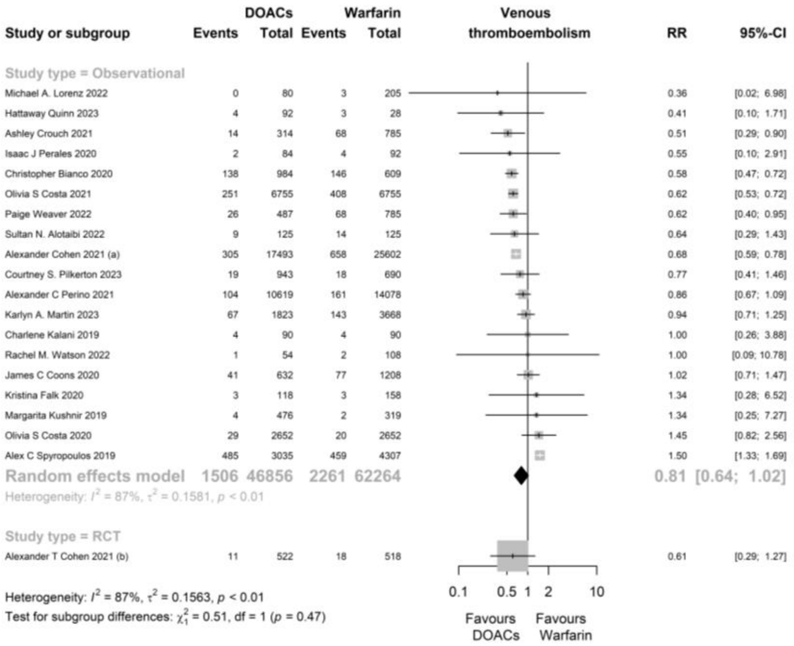

Figure S28. Subgroup analysis based on study type for the outcome of venous thromboembolism representing the comparison between direct oral anticoagulants (DOACs) and warfarin. RR, risk ratio; CI, confidence interval.

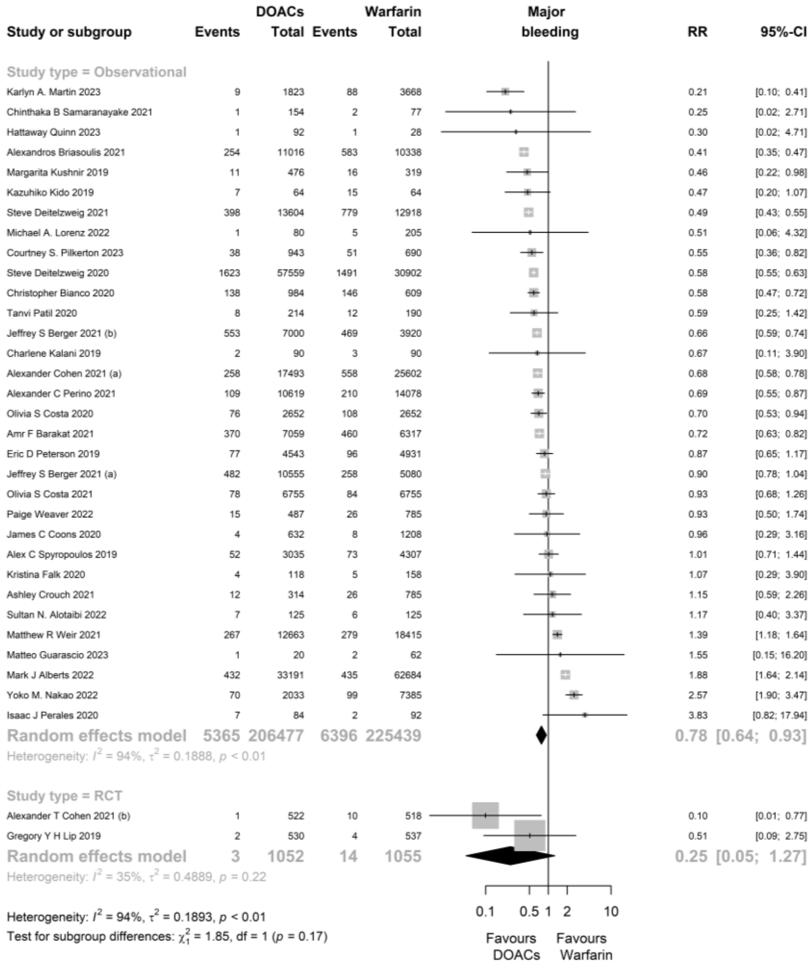

Figure S29. Subgroup analysis based on study type for the outcome of major bleeding representing the comparison between direct oral anticoagulants (DOACs) and warfarin. RR, risk ratio; CI, confidence interval.

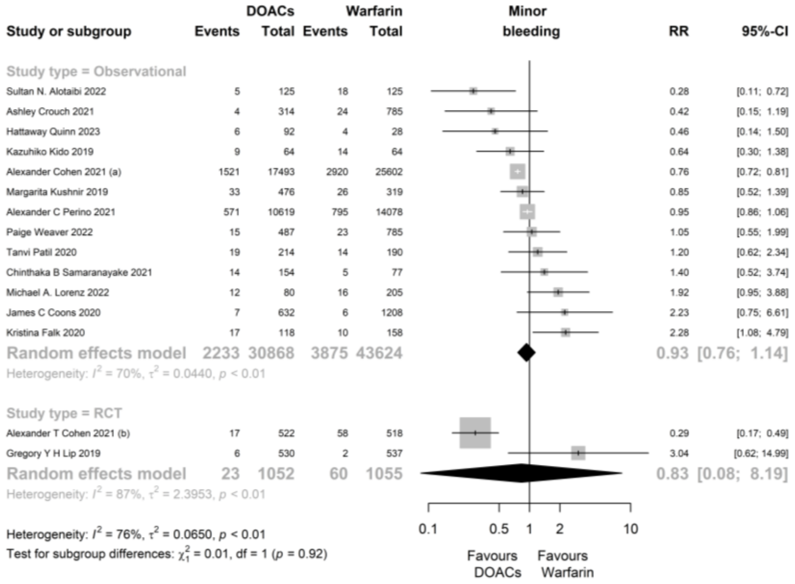

Figure S30. Subgroup analysis based on study type for the outcome of minor bleeding representing the comparison between direct oral anticoagulants (DOACs) and warfarin. RR, risk ratio; CI, confidence interval.

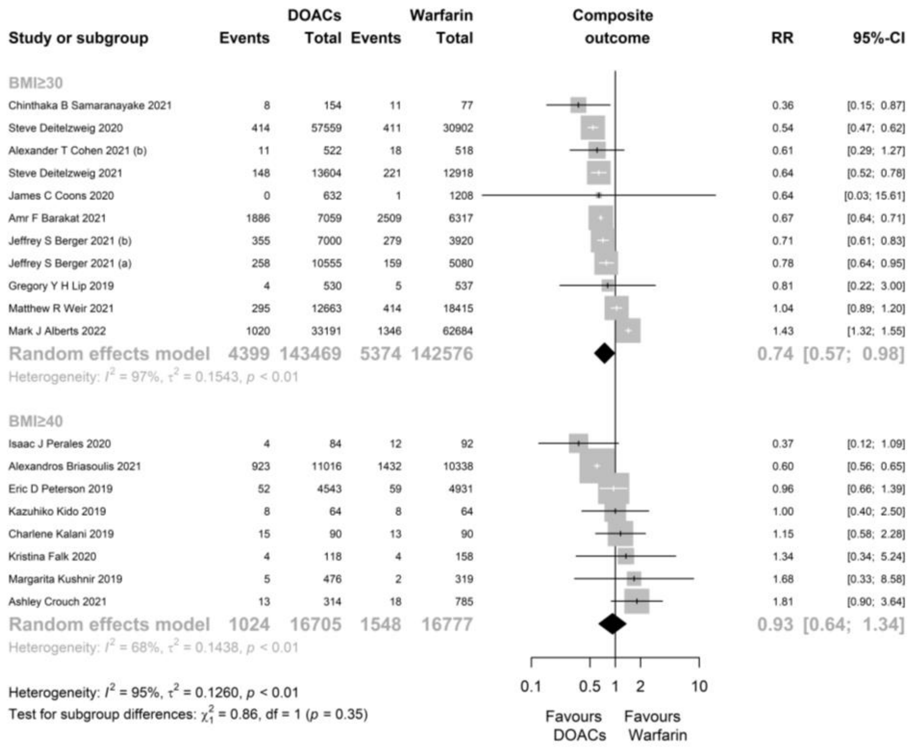

Figure S31. Subgroup analysis based on minimum body mass index (BMI) as inclusion criterion for the composite primary efficacy outcome representing the comparison between direct oral anticoagulants (DOACs) and warfarin. RR, risk ratio; CI, confidence interval.

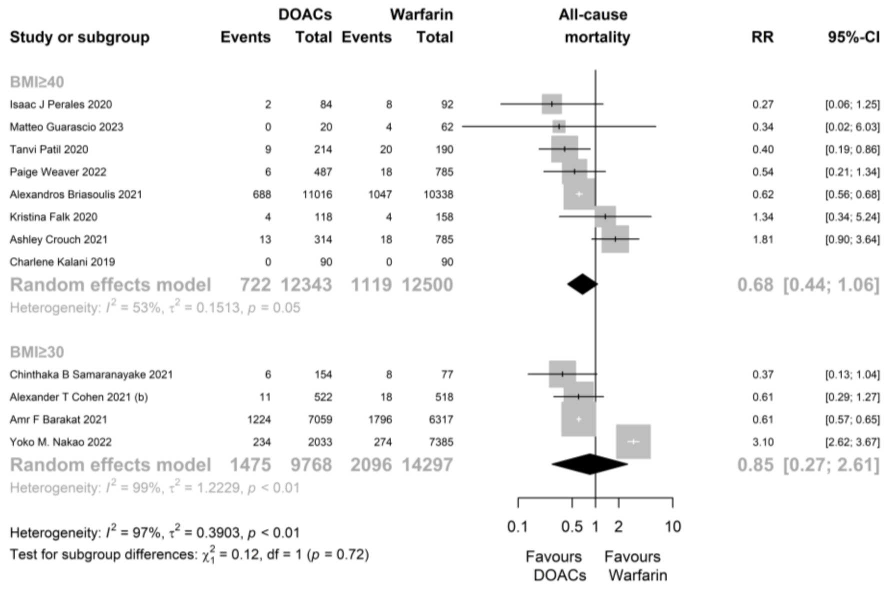

Figure S32. Subgroup analysis based on minimum body mass index (BMI) as inclusion criterion for the outcome of all-cause mortality representing the comparison between direct oral anticoagulants (DOACs) and warfarin. RR, risk ratio; CI, confidence interval.

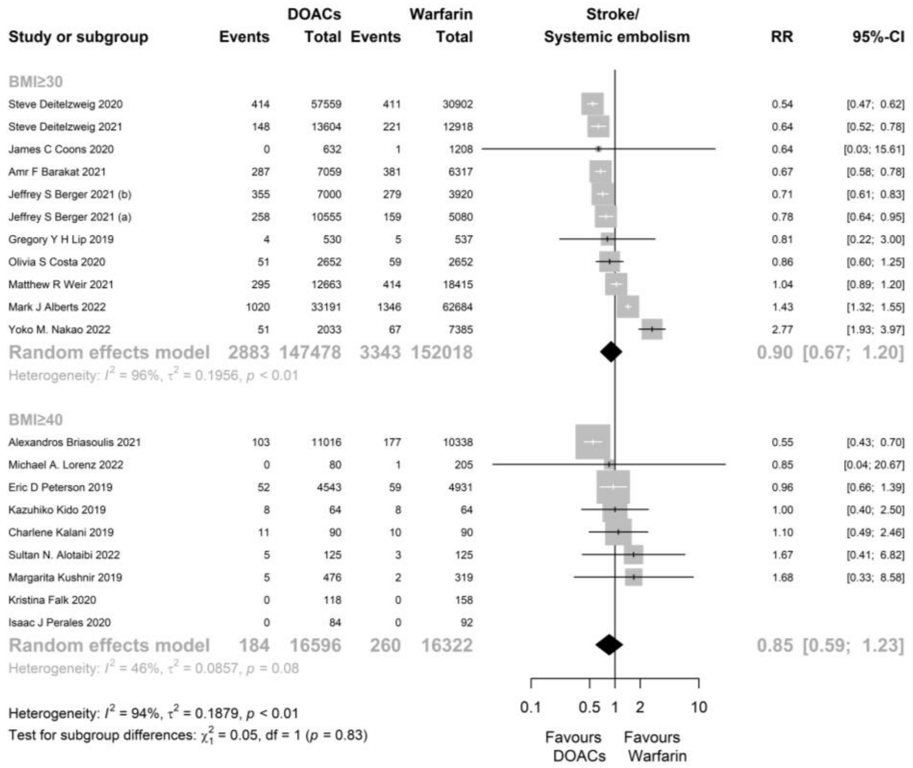

Figure S33. Subgroup analysis based on minimum body mass index (BMI) as inclusion criterion for the outcome of any stroke or systemic embolism representing the comparison between direct oral anticoagulants (DOACs) and warfarin. RR, risk ratio; CI, confidence interval.

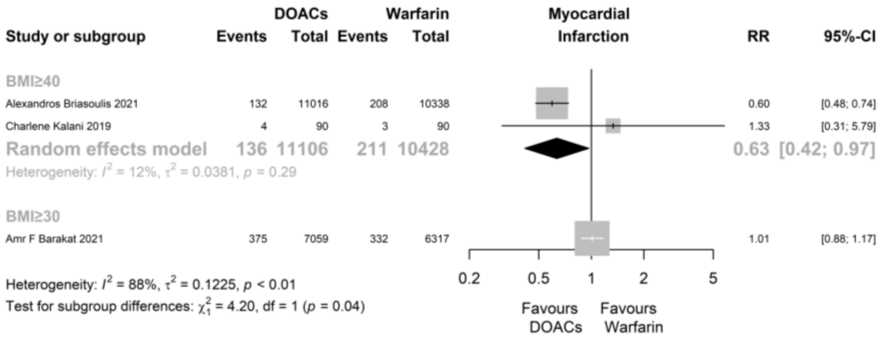

Figure S34. Subgroup analysis based on minimum body mass index (BMI) as inclusion criterion for the outcome of myocardial infarction representing the comparison between direct oral anticoagulants (DOACs) and warfarin. RR, risk ratio; CI, confidence interval.

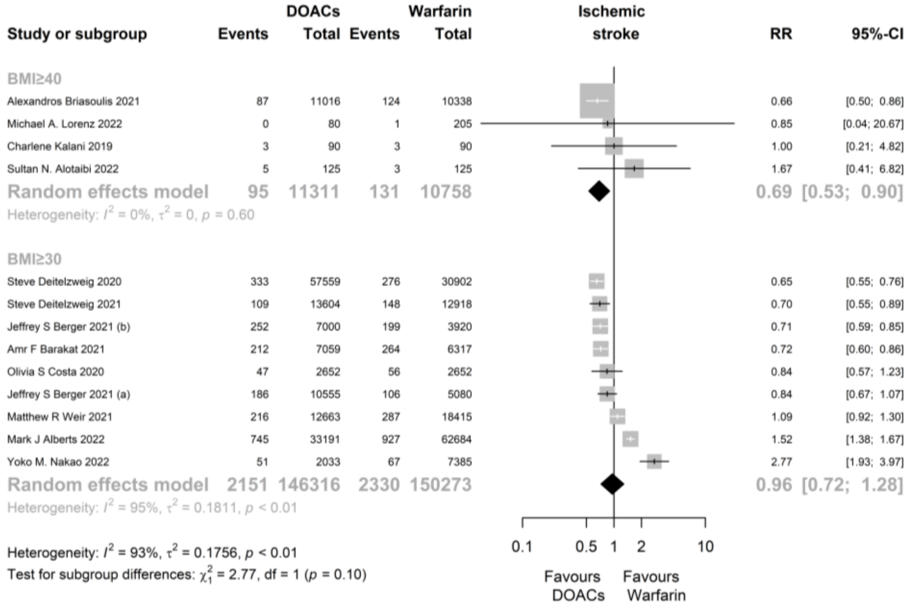

Figure S35. Subgroup analysis based on minimum body mass index (BMI) as inclusion criterion for the outcome of ischemic stroke representing the comparison between direct oral anticoagulants (DOACs) and warfarin. RR, risk ratio; CI, confidence interval.

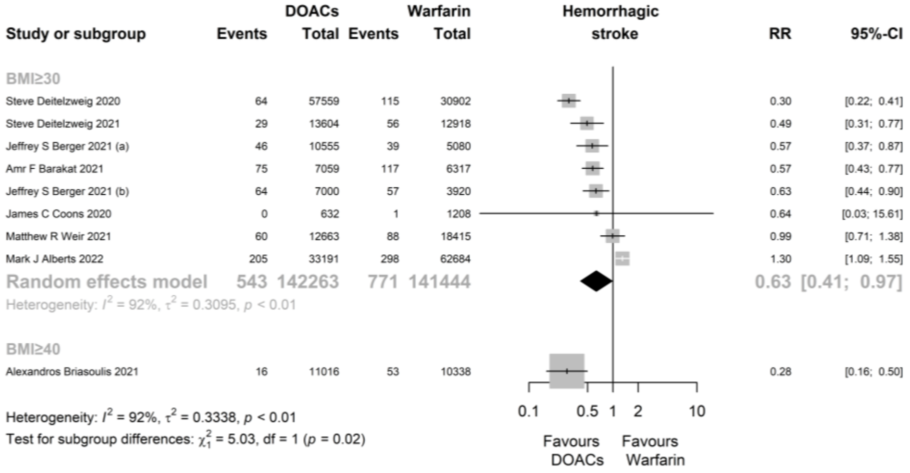

Figure S36. Subgroup analysis based on minimum body mass index (BMI) as inclusion criterion for the outcome of hemorrhagic stroke representing the comparison between direct oral anticoagulants (DOACs) and warfarin. RR, risk ratio; CI, confidence interval.

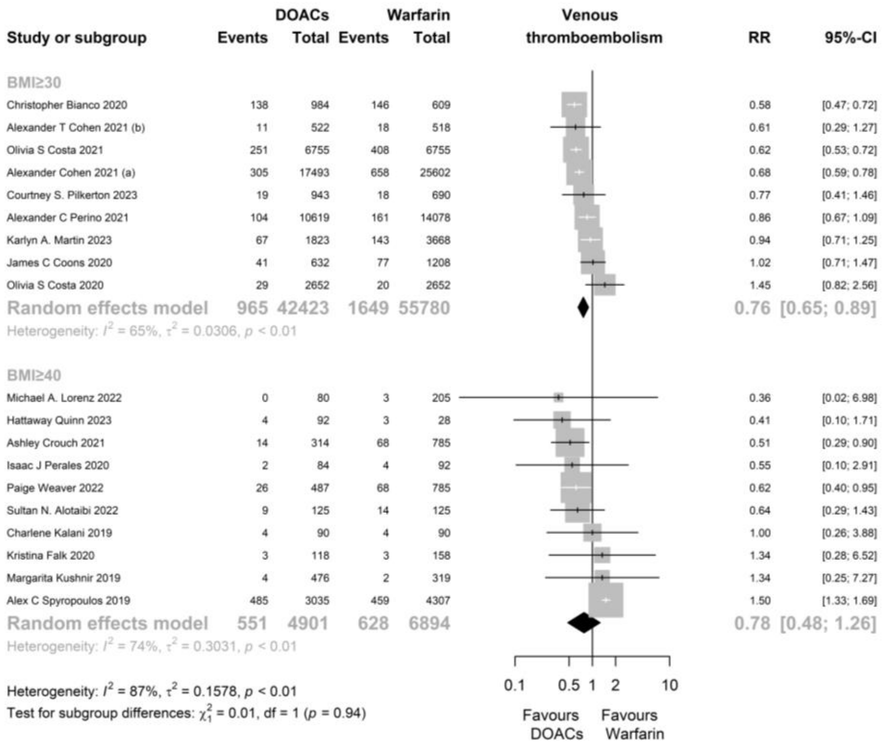

Figure S37. Subgroup analysis based on minimum body mass index (BMI) as inclusion criterion for the outcome of venous thromboembolism representing the comparison between direct oral anticoagulants (DOACs) and warfarin. RR, risk ratio; CI, confidence interval.

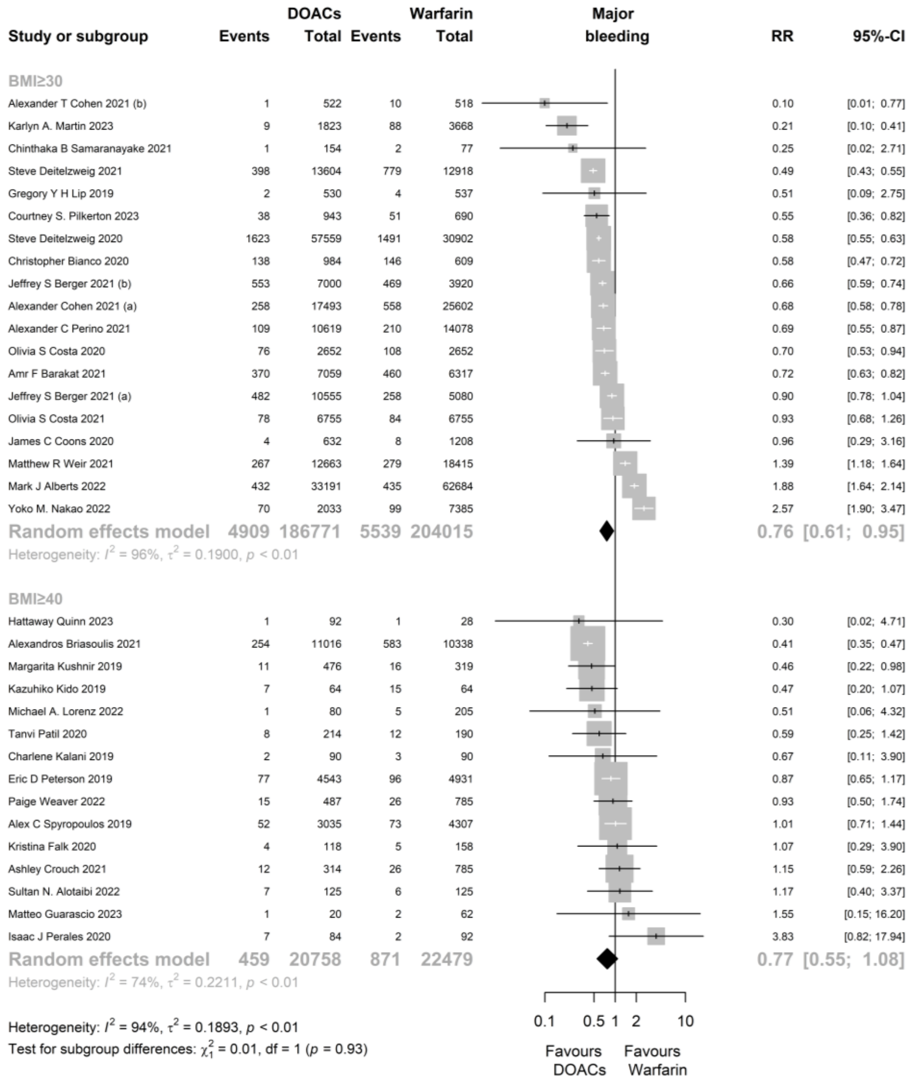

Figure S38. Subgroup analysis based on minimum body mass index (BMI) as inclusion criterion for the outcome of major bleeding representing the comparison between direct oral anticoagulants (DOACs) and warfarin. RR, risk ratio; CI, confidence interval.

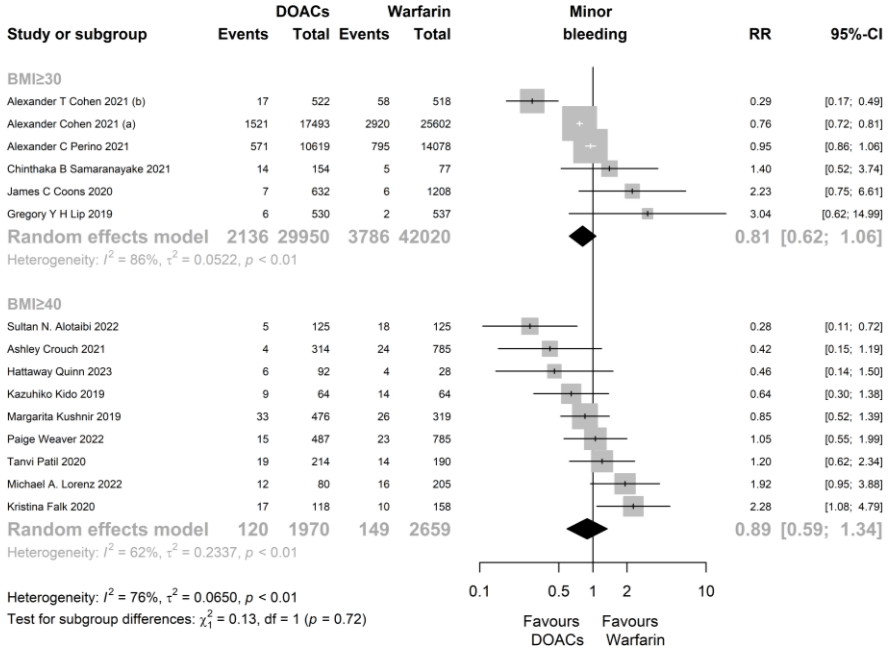

Figure S39. Subgroup analysis based on minimum body mass index (BMI) as inclusion criterion for the outcome of minor bleeding representing the comparison between direct oral anticoagulants (DOACs) and warfarin. RR, risk ratio; CI, confidence interval.

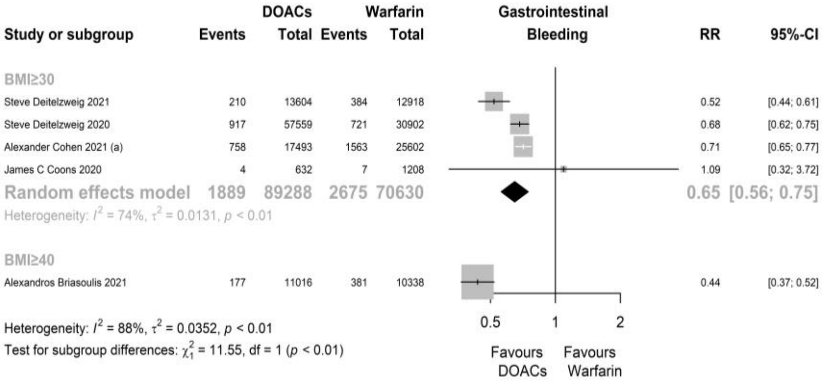

Figure S40. Subgroup analysis based on minimum body mass index (BMI) as inclusion criterion for the outcome of gastrointestinal bleeding representing the comparison between direct oral anticoagulants (DOACs) and warfarin. RR, risk ratio; CI, confidence interval.

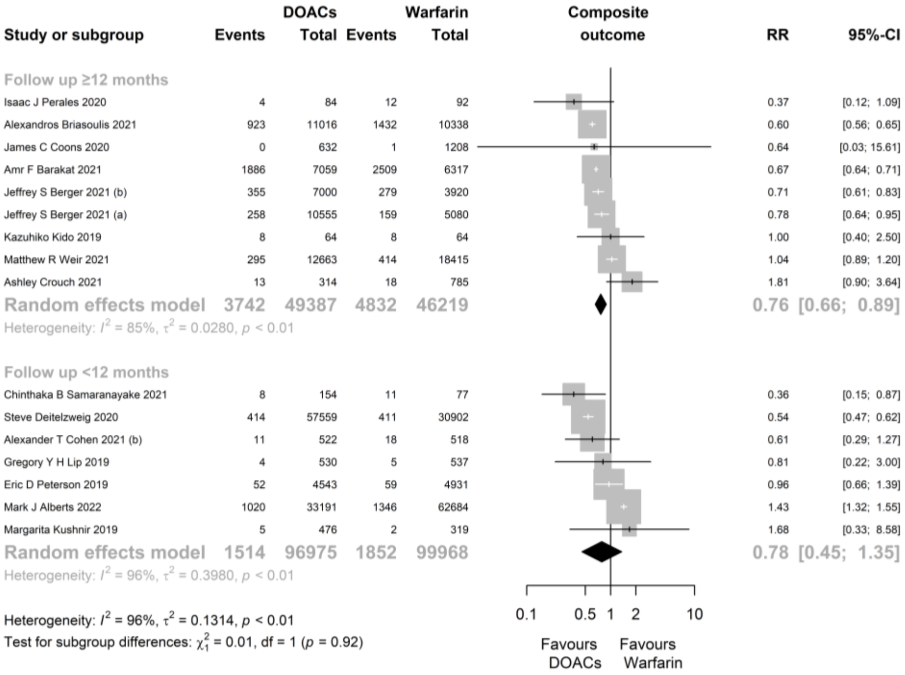

Figure S41. Subgroup analysis based on follow up duration for the composite primary efficacy outcome representing the comparison between direct oral anticoagulants (DOACs) and warfarin. RR, risk ratio; CI, confidence interval.

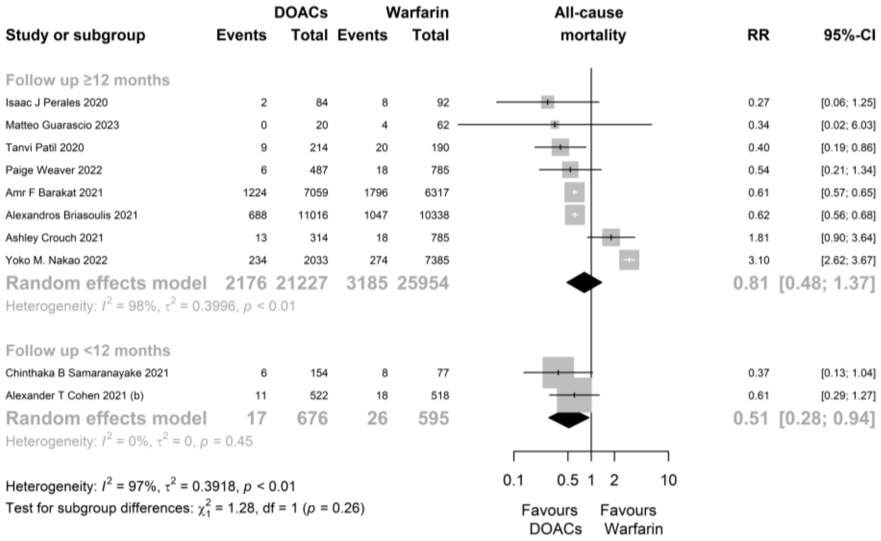

Figure S42. Subgroup analysis based on follow up duration for the outcome of all-cause mortality representing the comparison between direct oral anticoagulants (DOACs) and warfarin. RR, risk ratio; CI, confidence interval.

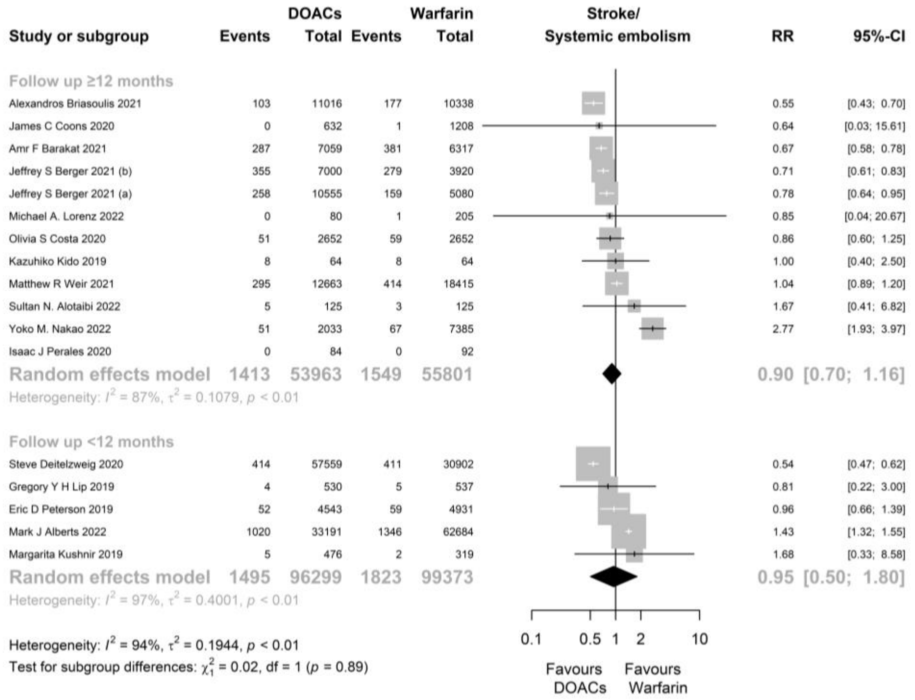

Figure S43. Subgroup analysis based on follow up duration for the outcome of any stroke or systemic embolism representing the comparison between direct oral anticoagulants (DOACs) and warfarin. RR, risk ratio; CI, confidence interval.

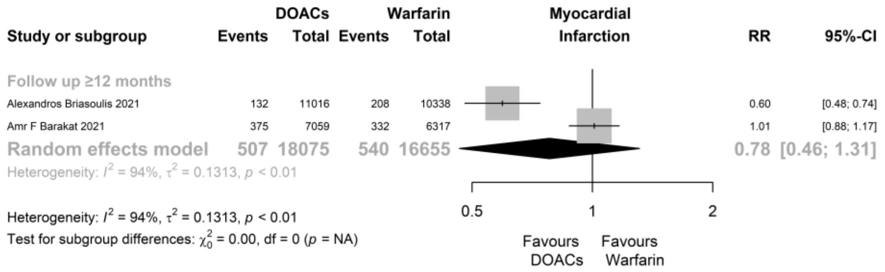

Figure S44. Subgroup analysis based on follow up duration for the outcome of myocardial infarction representing the comparison between direct oral anticoagulants (DOACs) and warfarin. RR, risk ratio; CI, confidence interval.

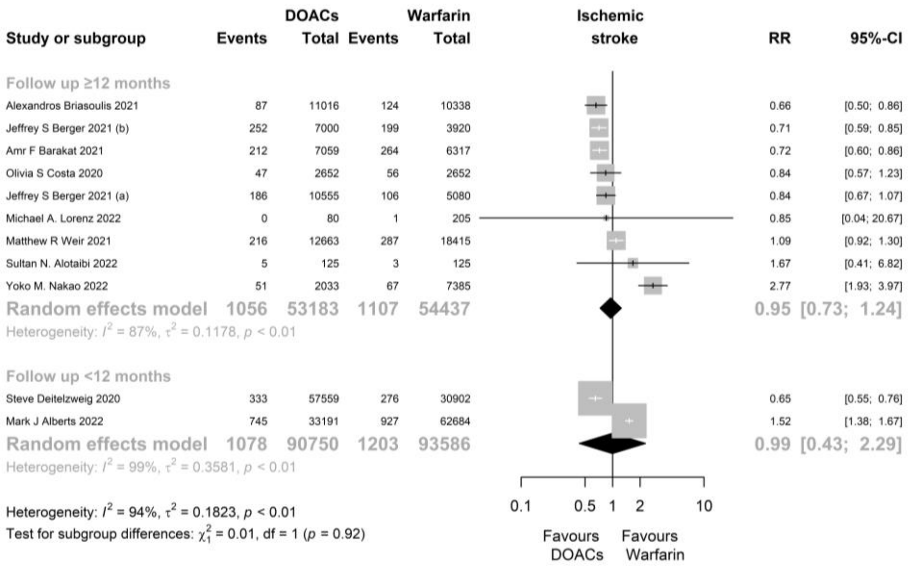

Figure S45. Subgroup analysis based on follow up duration for the outcome of ischemic stroke representing the comparison between direct oral anticoagulants (DOACs) and warfarin. RR, risk ratio; CI, confidence interval.

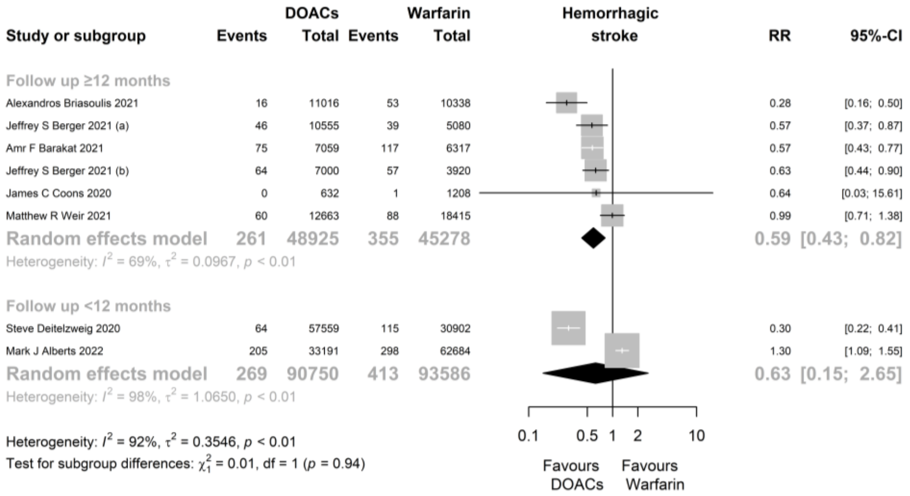

Figure S46. Subgroup analysis based on follow up duration for the outcome of hemorrhagic stroke representing the comparison between direct oral anticoagulants (DOACs) and warfarin. RR, risk ratio; CI, confidence interval.

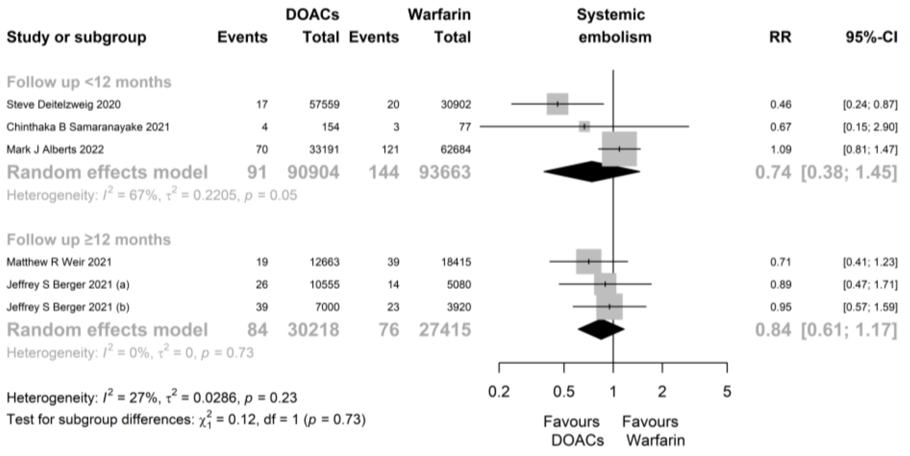

Figure S47. Subgroup analysis based on follow up duration for the outcome of systemic embolism representing the comparison between direct oral anticoagulants (DOACs) and warfarin. RR, risk ratio; CI, confidence interval.

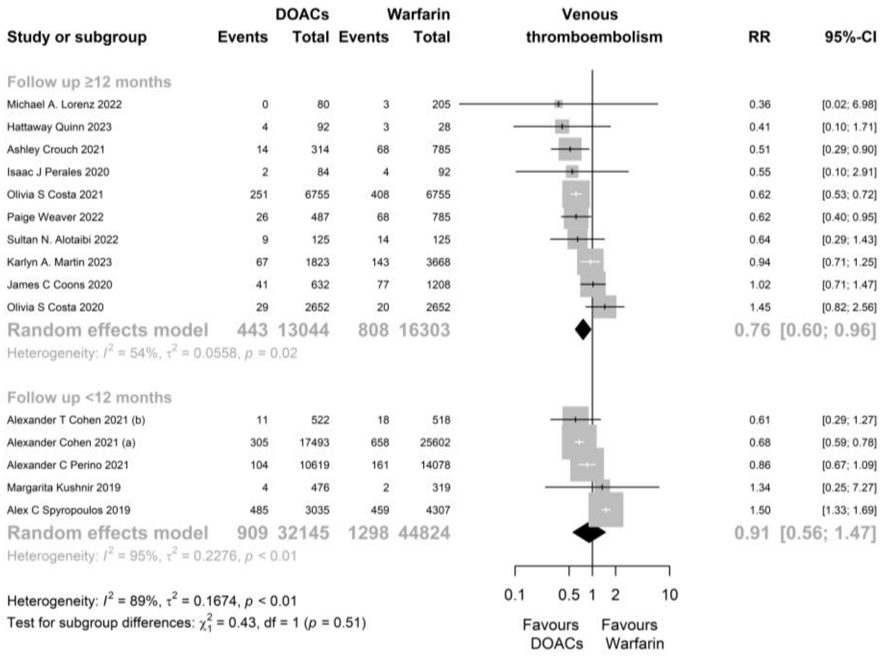

Figure S48. Subgroup analysis based on follow up duration for the outcome of venous thromboembolism representing the comparison between direct oral anticoagulants (DOACs) and warfarin. RR, risk ratio; CI, confidence interval.

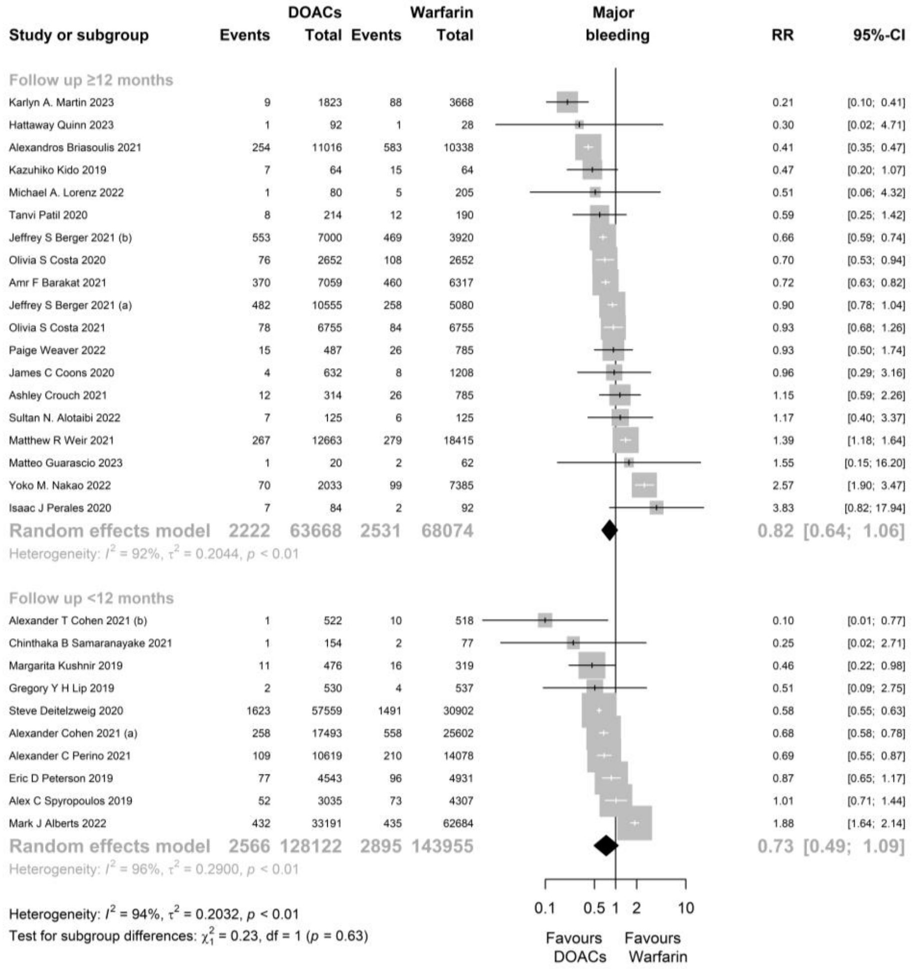

Figure S49. Subgroup analysis based on follow up duration for the outcome of major bleeding representing the comparison between direct oral anticoagulants (DOACs) and warfarin. RR, risk ratio; CI, confidence interval.

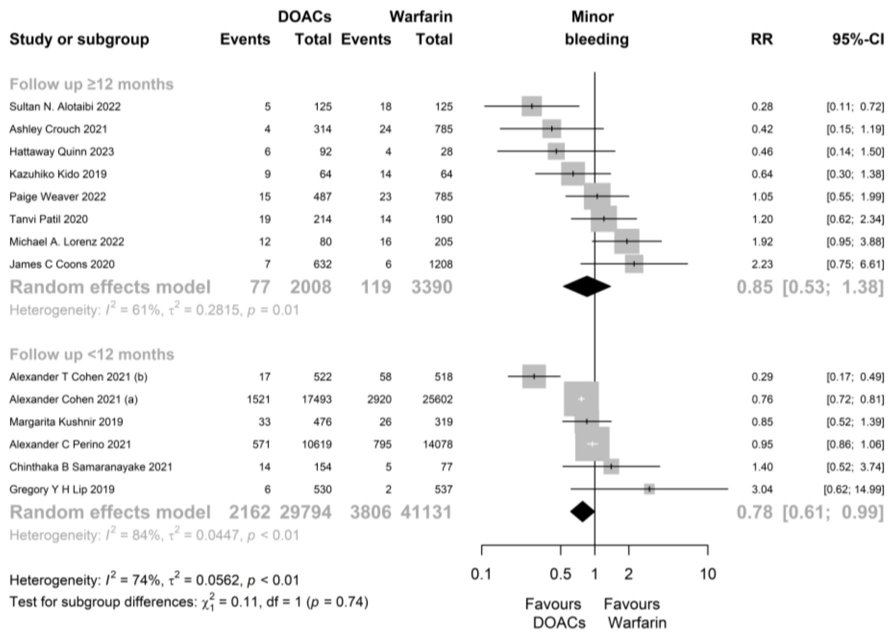

Figure S50. Subgroup analysis based on follow up duration for the outcome of minor bleeding representing the comparison between direct oral anticoagulants (DOACs) and warfarin. RR, risk ratio; CI, confidence interval.

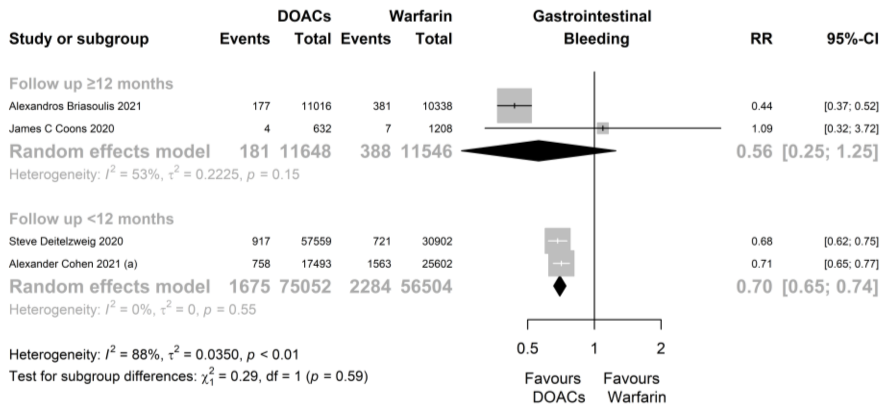

Figure S51. Subgroup analysis based on follow up duration for the outcome of gastrointestinal bleeding representing the comparison between direct oral anticoagulants (DOACs) and warfarin. RR, risk ratio; CI, confidence interval.

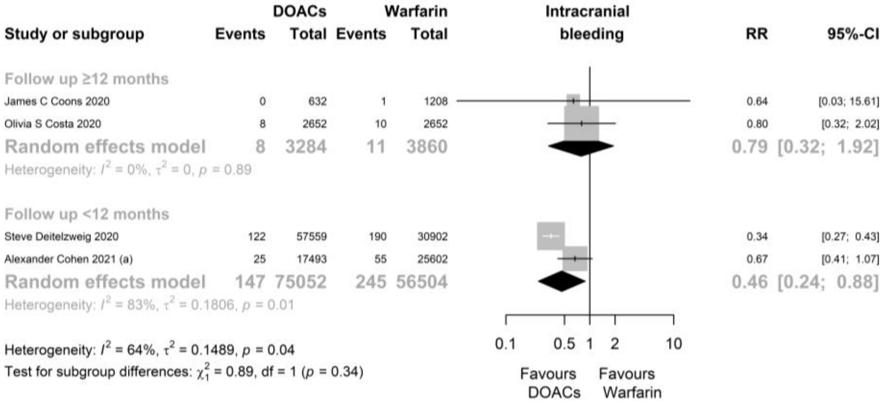

Figure S52. Subgroup analysis based on follow up duration for the outcome of intracranial bleeding representing the comparison between direct oral anticoagulants (DOACs) and warfarin. RR, risk ratio; CI, confidence interval.

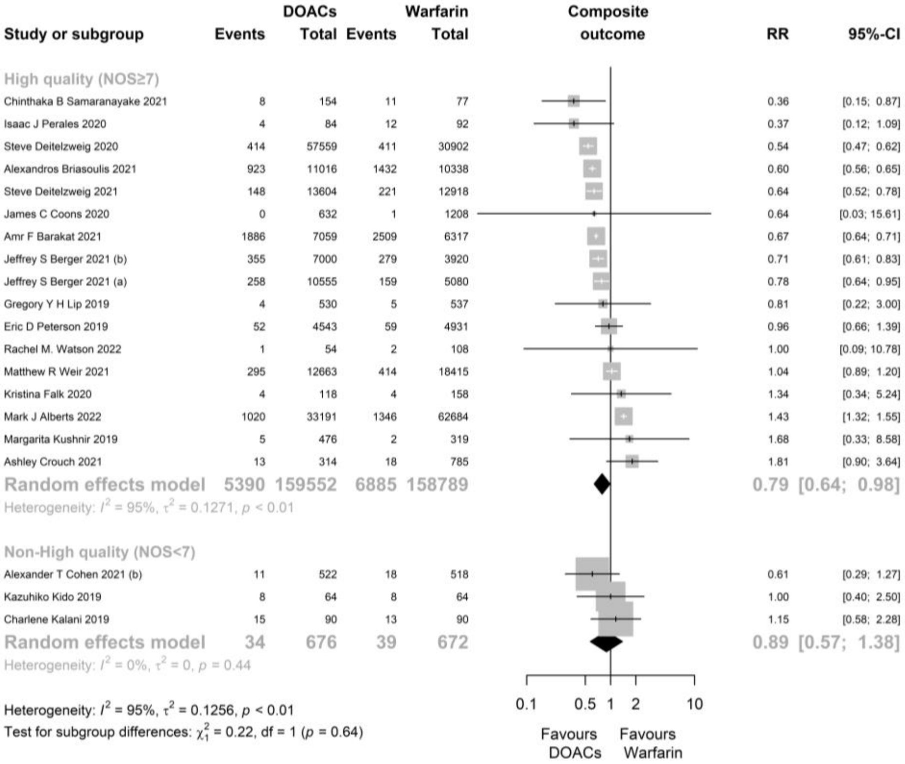

Figure S53. Subgroup analysis based on study quality for the composite primary efficacy outcome representing the comparison between direct oral anticoagulants (DOACs) and warfarin. RR, risk ratio; CI, confidence interval.

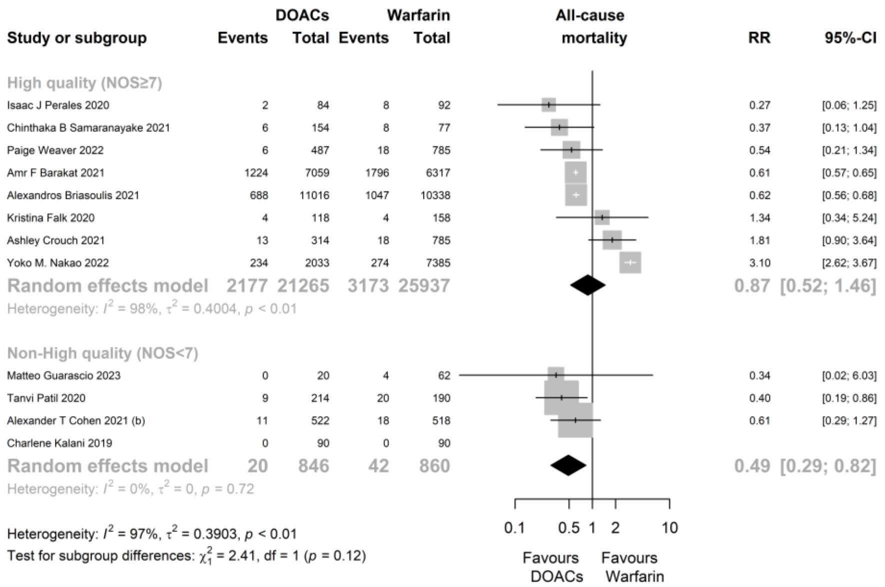

Figure S54. Subgroup analysis based on study quality for the outcome of all-cause mortality representing the comparison between direct oral anticoagulants (DOACs) and warfarin. RR, risk ratio; CI, confidence interval.

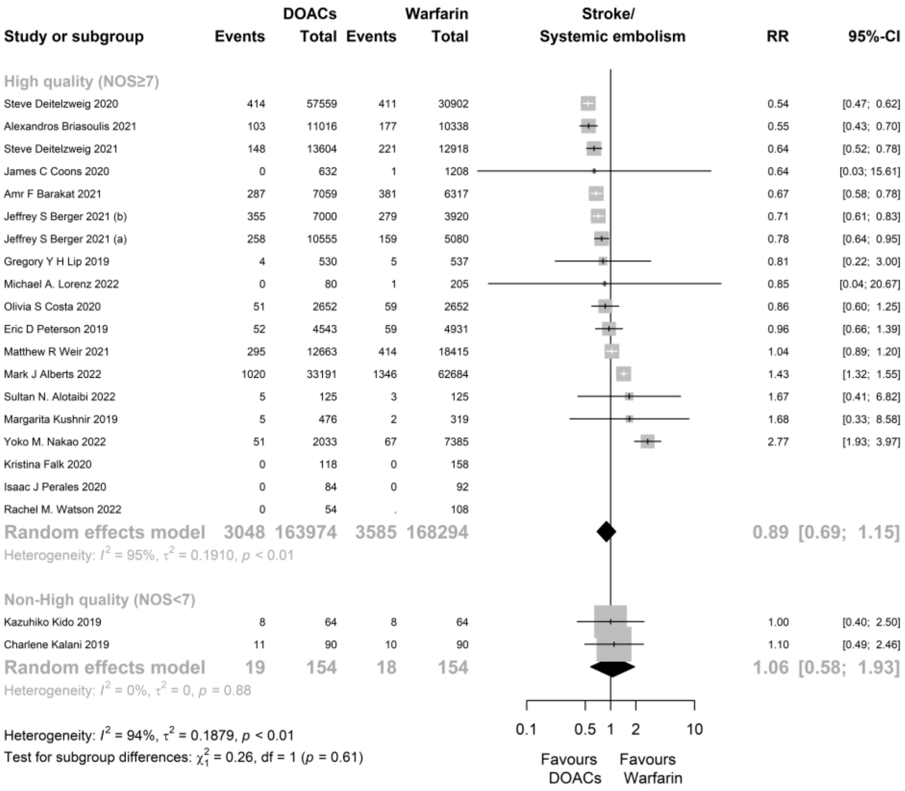

Figure S55. Subgroup analysis based on study quality for the outcome of any stroke or systemic embolism representing the comparison between direct oral anticoagulants (DOACs) and warfarin. RR, risk ratio; CI, confidence interval.

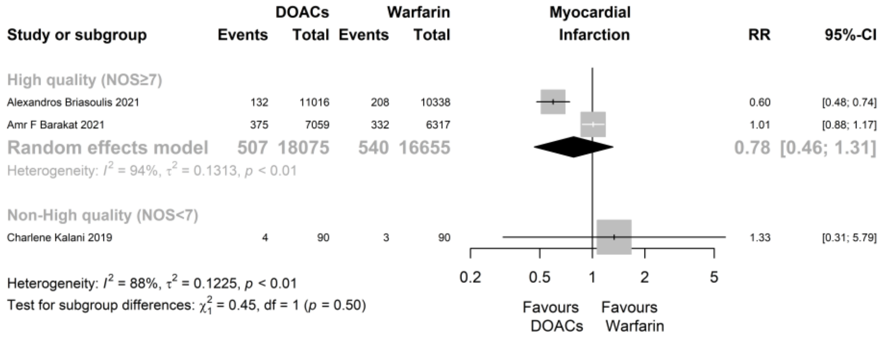

Figure S56. Subgroup analysis based on study quality for the outcome of myocardial infarction representing the comparison between direct oral anticoagulants (DOACs) and warfarin. RR, risk ratio; CI, confidence interval.

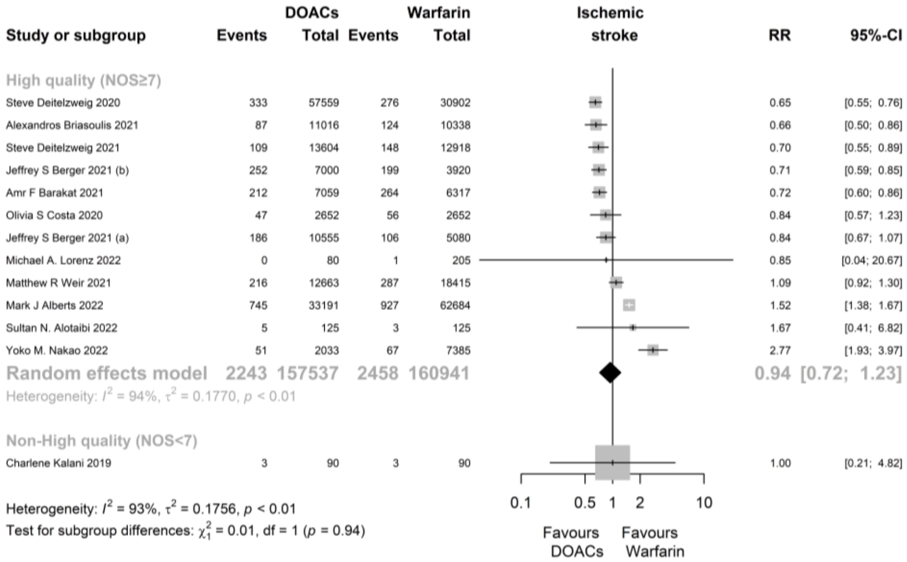

Figure S57. Subgroup analysis based on study quality for the outcome of ischemic stroke representing the comparison between direct oral anticoagulants (DOACs) and warfarin. RR, risk ratio; CI, confidence interval.

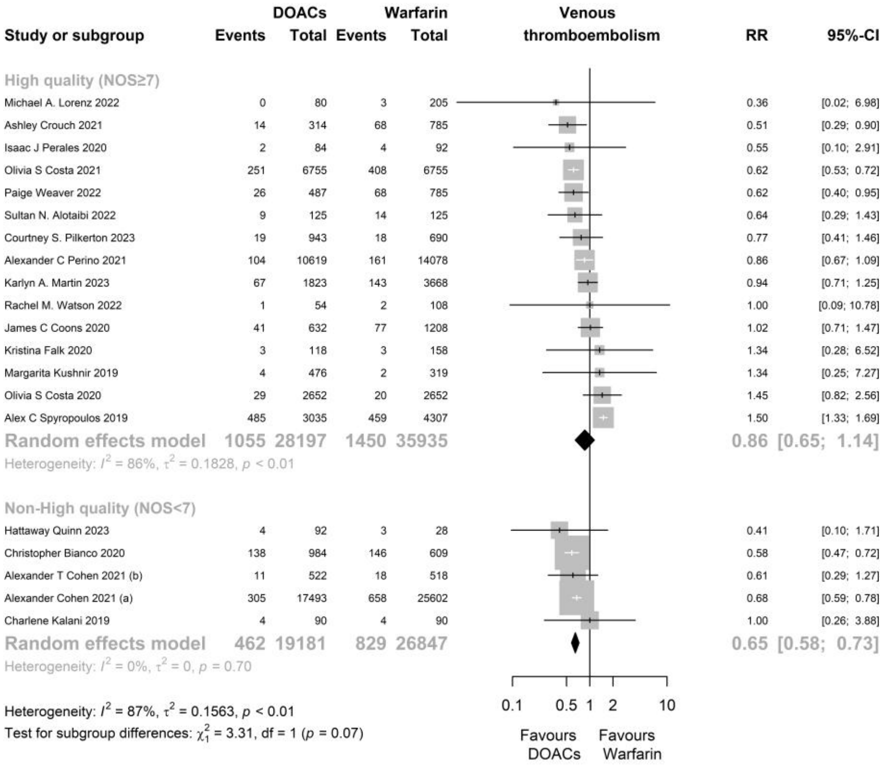

Figure S58. Subgroup analysis based on study quality for the outcome of venous thromboembolism representing the comparison between direct oral anticoagulants (DOACs) and warfarin. RR, risk ratio; CI, confidence interval.

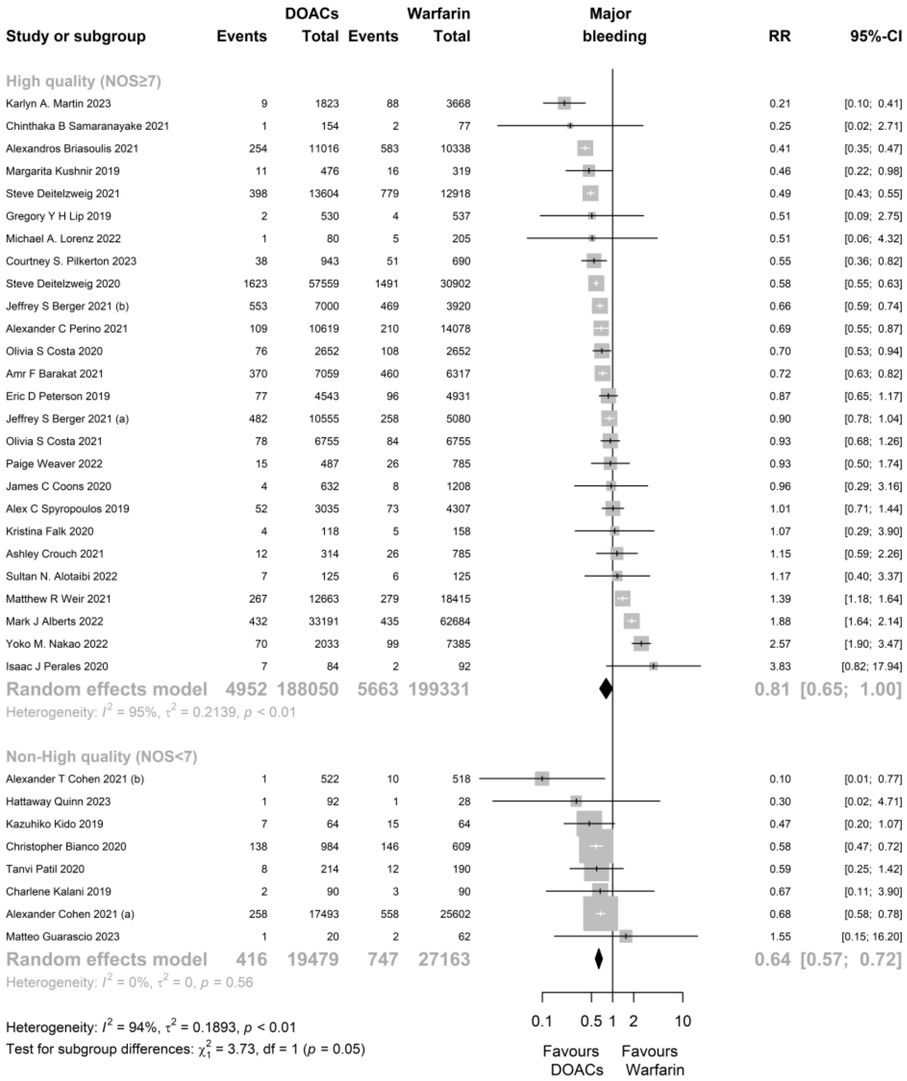

Figure S59. Subgroup analysis based on study quality for the outcome of major bleeding representing the comparison between direct oral anticoagulants (DOACs) and warfarin. RR, risk ratio; CI, confidence interval.

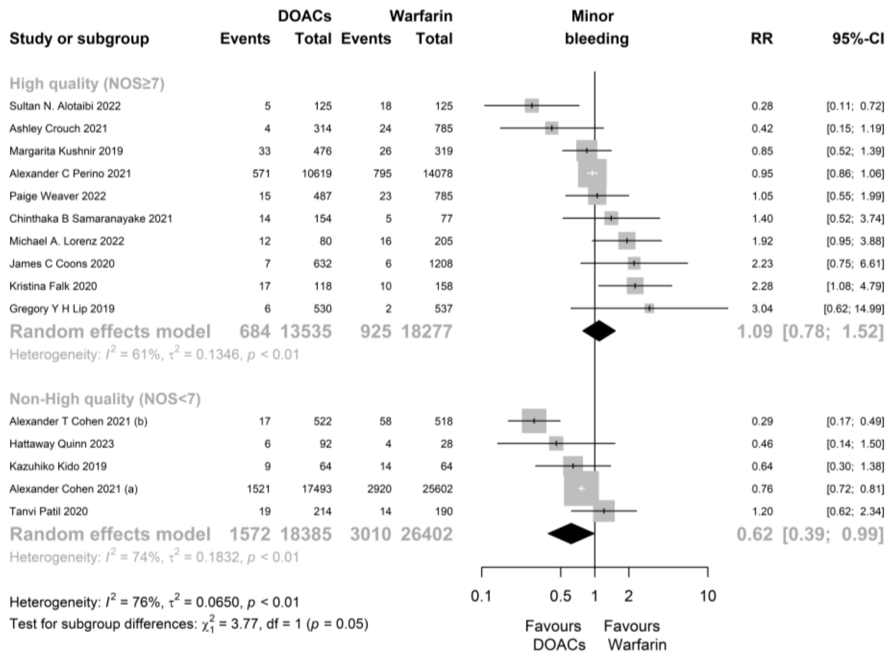

Figure S60. Subgroup analysis based on study quality for the outcome of minor bleeding representing the comparison between direct oral anticoagulants (DOACs) and warfarin. RR, risk ratio; CI, confidence interval.

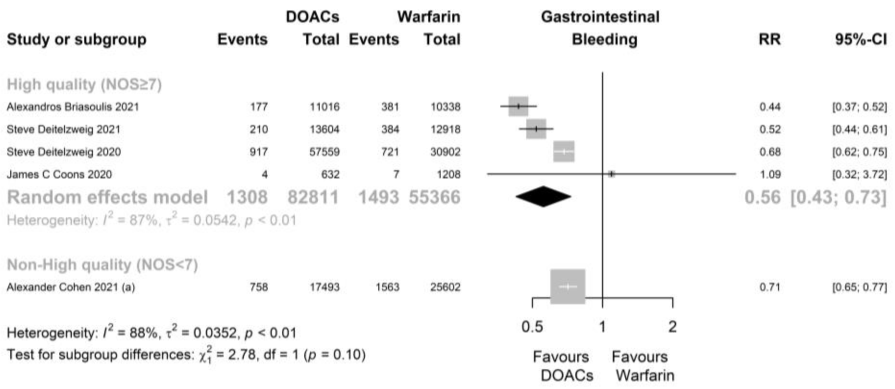

Figure S61. Subgroup analysis based on study quality for the outcome of gastrointestinal bleeding representing the comparison between direct oral anticoagulants (DOACs) and warfarin. RR, risk ratio; CI, confidence interval.

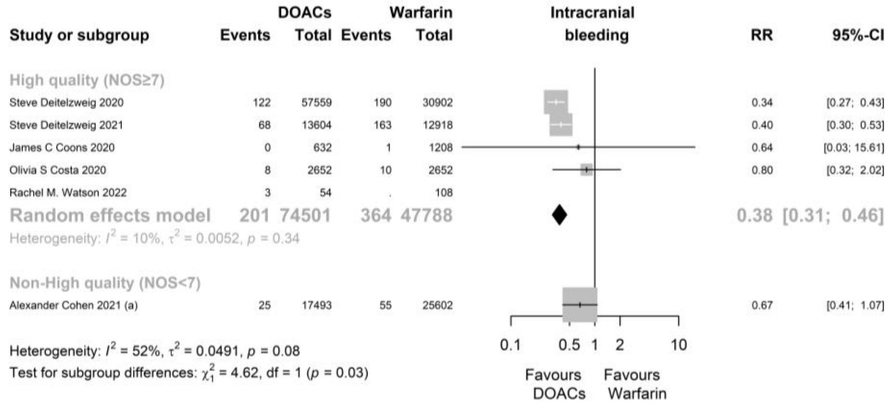

Figure S62. Subgroup analysis based on study quality for the outcome of intracranial bleeding representing the comparison between direct oral anticoagulants (DOACs) and warfarin. RR, risk ratio; CI, confidence interval.

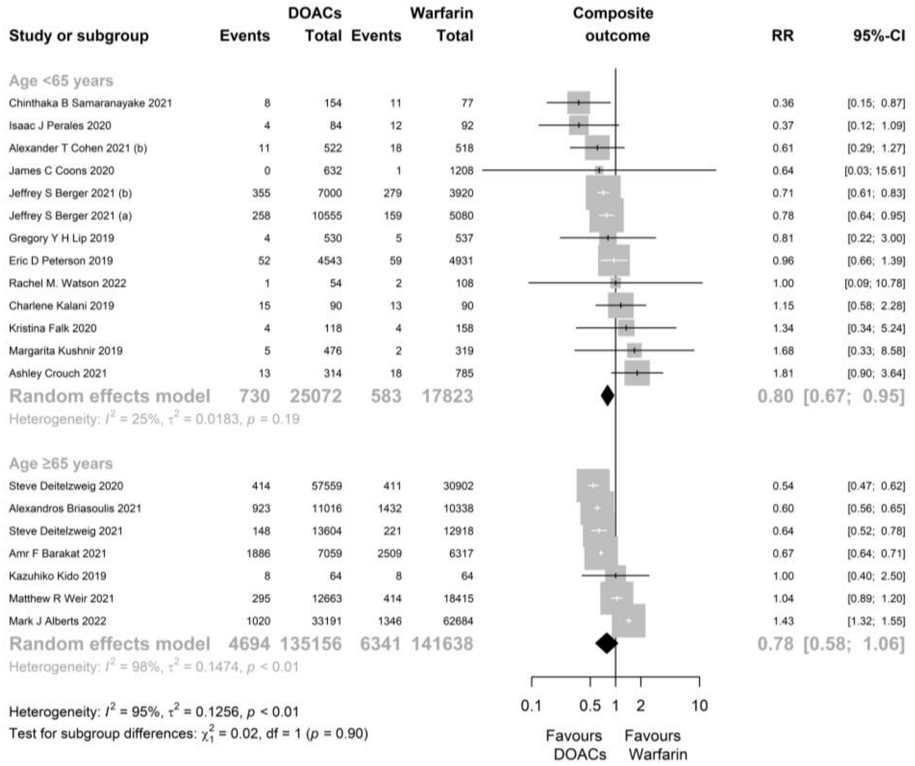

Figure S63. Subgroup analysis based on age for the composite primary efficacy outcome representing the comparison between direct oral anticoagulants (DOACs) and warfarin. RR, risk ratio; CI, confidence interval.

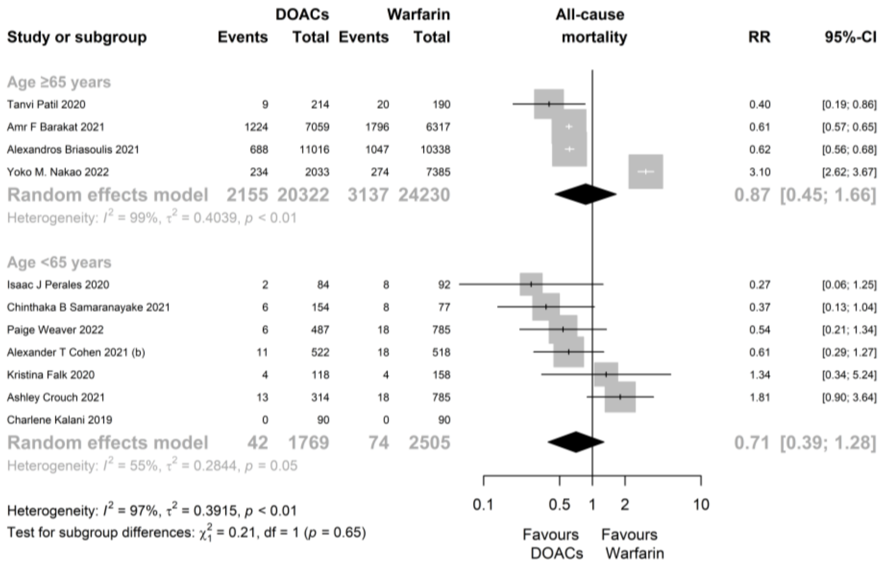

Figure S64. Subgroup analysis based on age for the outcome of all-cause mortality representing the comparison between direct oral anticoagulants (DOACs) and warfarin. RR, risk ratio; CI, confidence interval.

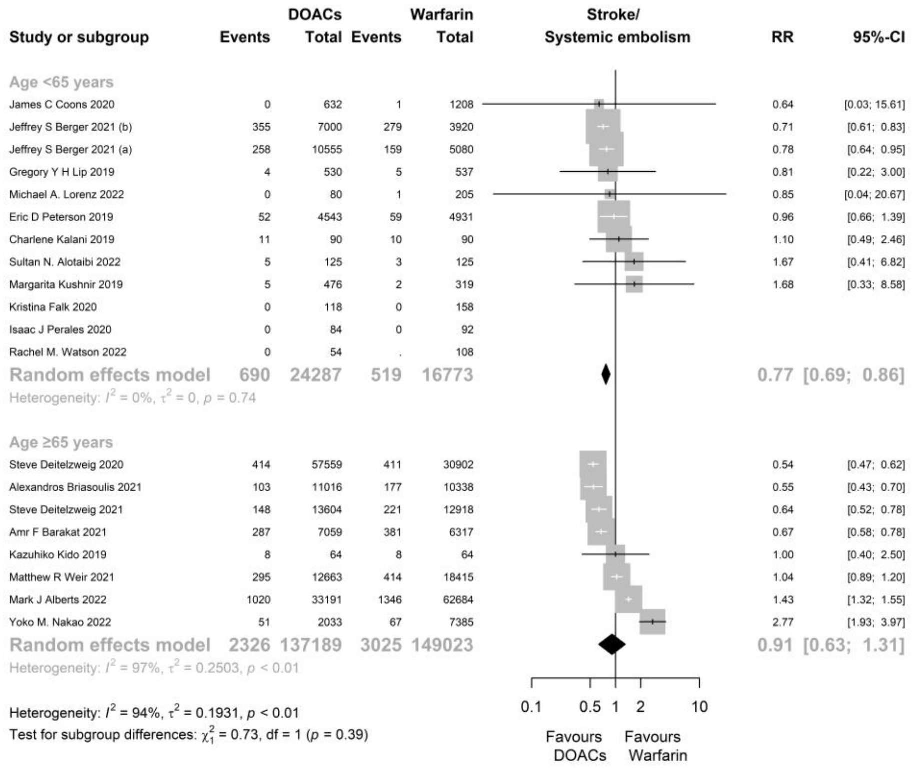

Figure S65. Subgroup analysis based on age for the outcome of any stroke or systemic embolism representing the comparison between direct oral anticoagulants (DOACs) and warfarin. RR, risk ratio; CI, confidence interval.

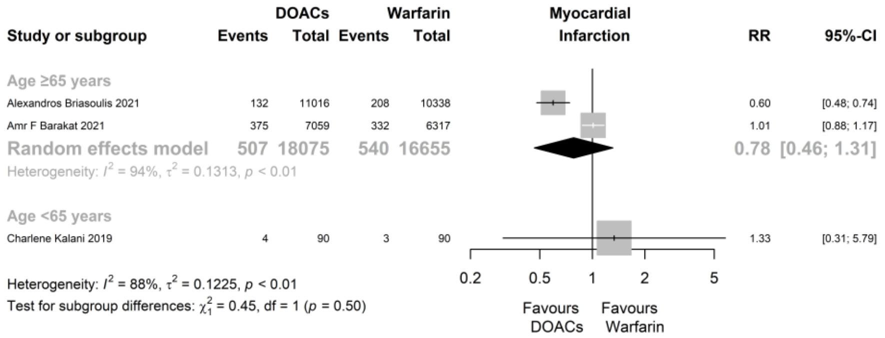

Figure S66. Subgroup analysis based on age for the outcome of myocardial infarction representing the comparison between direct oral anticoagulants (DOACs) and warfarin. RR, risk ratio; CI, confidence interval.

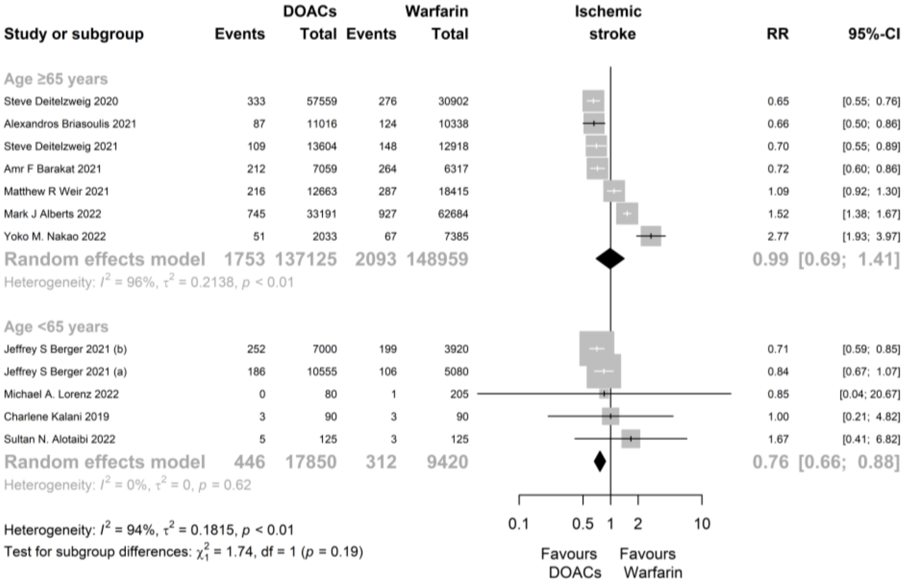

Figure S67. Subgroup analysis based on age for the outcome of ischemic stroke representing the comparison between direct oral anticoagulants (DOACs) and warfarin. RR, risk ratio; CI, confidence interval.

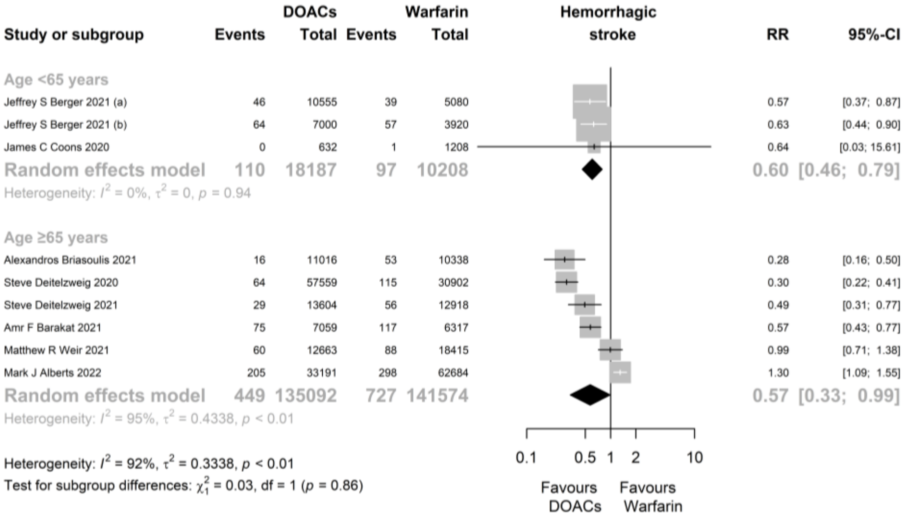

Figure S68. Subgroup analysis based on age for the outcome of hemorrhagic stroke representing the comparison between direct oral anticoagulants (DOACs) and warfarin. RR, risk ratio; CI, confidence interval.

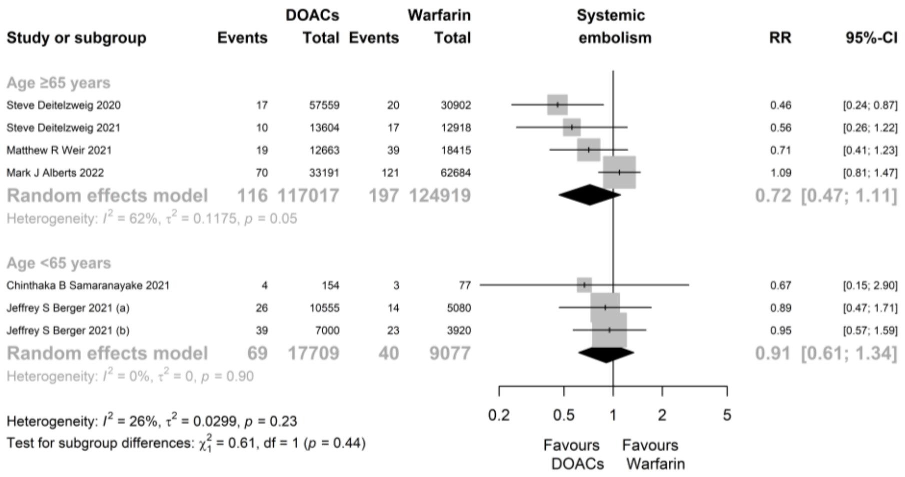

Figure S69. Subgroup analysis based on age for the outcome of systemic embolism representing the comparison between direct oral anticoagulants (DOACs) and warfarin. RR, risk ratio; CI, confidence interval.

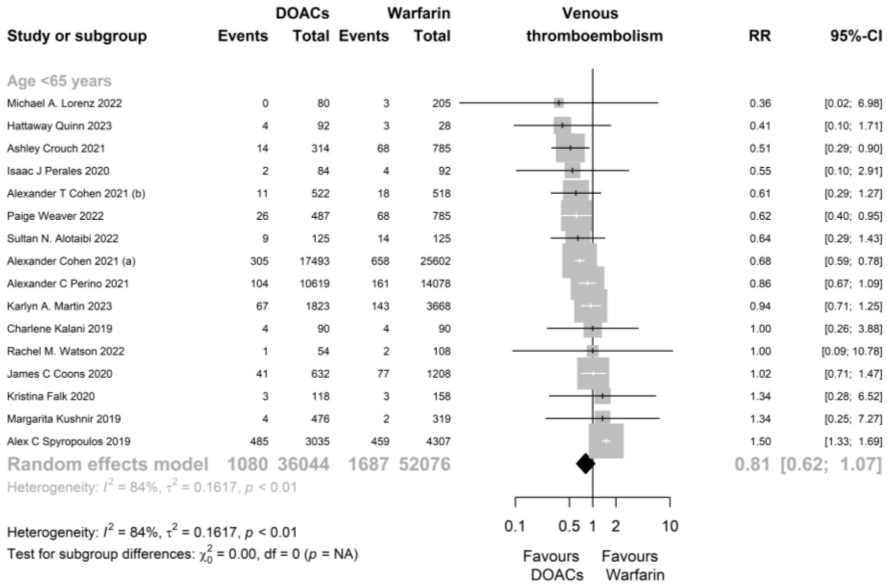

Figure S70. Subgroup analysis based on age for the outcome of venous thromboembolism representing the comparison between direct oral anticoagulants (DOACs) and warfarin. RR, risk ratio; CI, confidence interval.

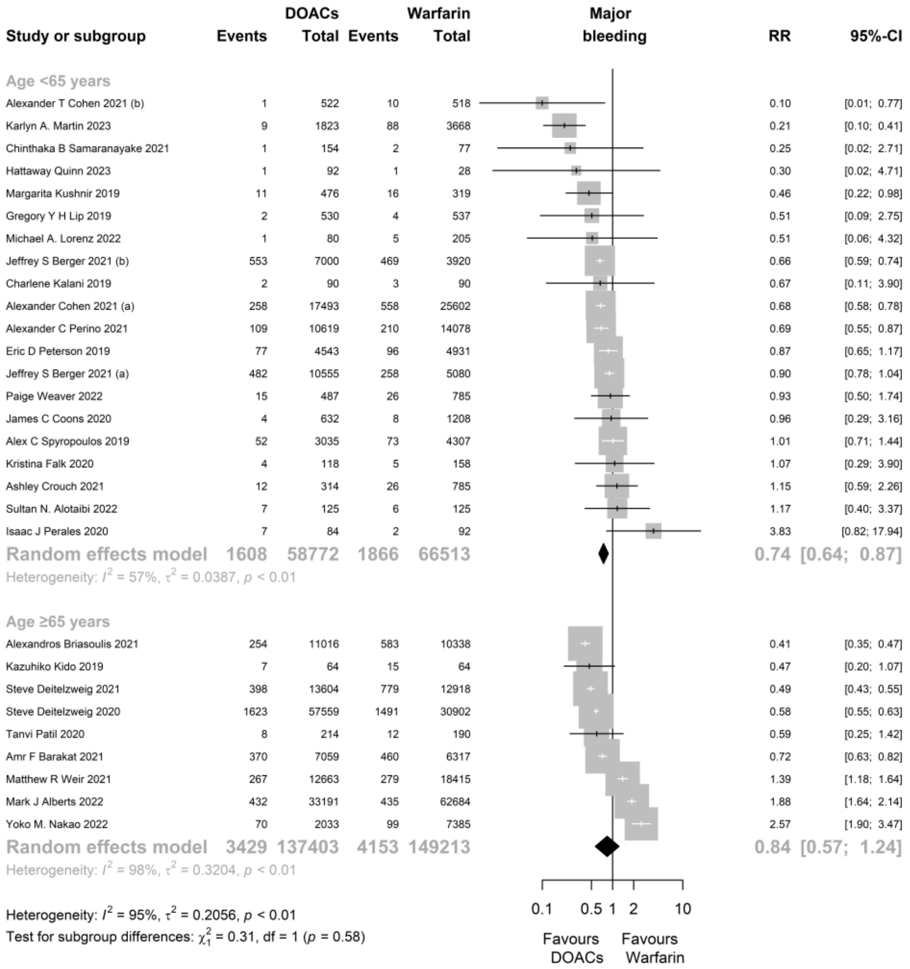

Figure S71. Subgroup analysis based on age for the outcome of major bleeding representing the comparison between direct oral anticoagulants (DOACs) and warfarin. RR, risk ratio; CI, confidence interval.

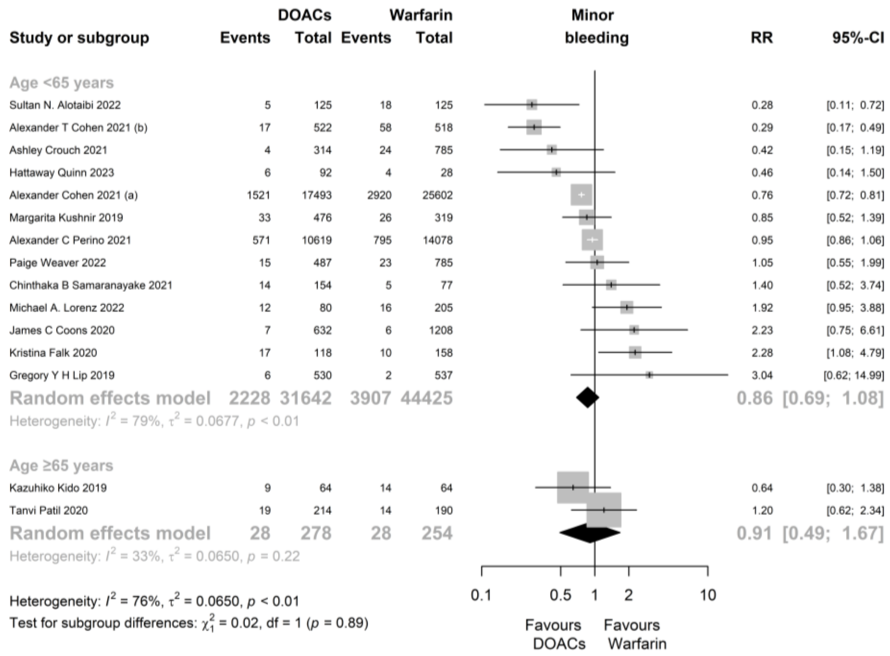

Figure S72. Subgroup analysis based on age for the outcome of minor bleeding representing the comparison between direct oral anticoagulants (DOACs) and warfarin. RR, risk ratio; CI, confidence interval.

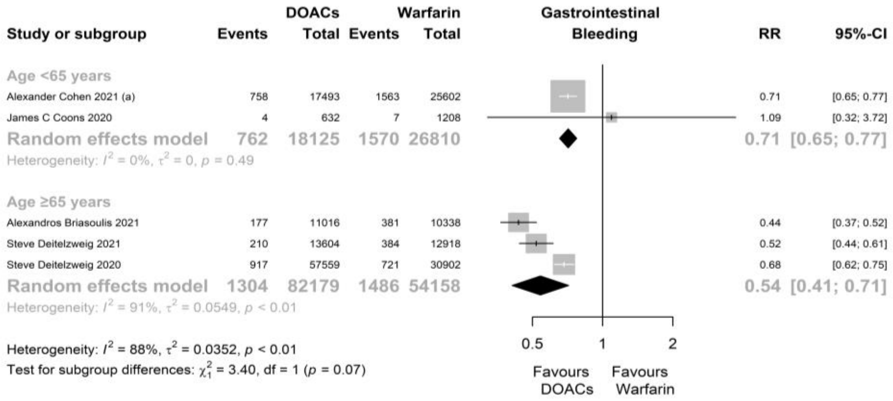

Figure S73. Subgroup analysis based on age for the outcome of gastrointestinal bleeding representing the comparison between direct oral anticoagulants (DOACs) and warfarin. RR, risk ratio; CI, confidence interval.

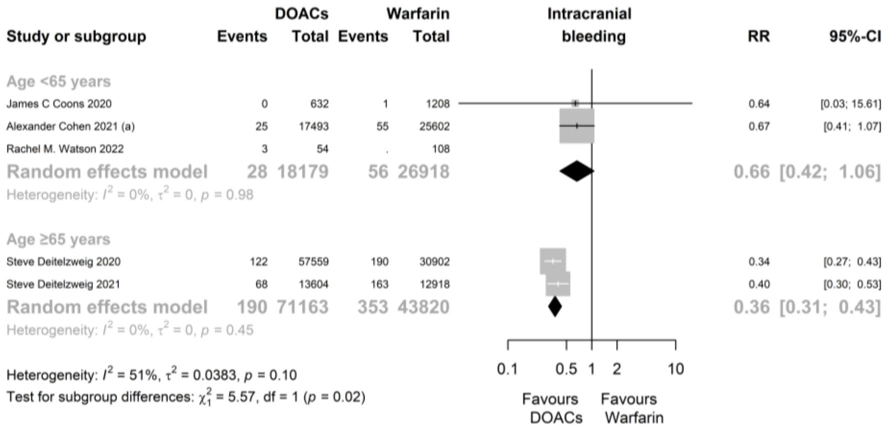

Figure S74. Subgroup analysis based on age for the outcome of intracranial bleeding representing the comparison between direct oral anticoagulants (DOACs) and warfarin. RR, risk ratio; CI, confidence interval.

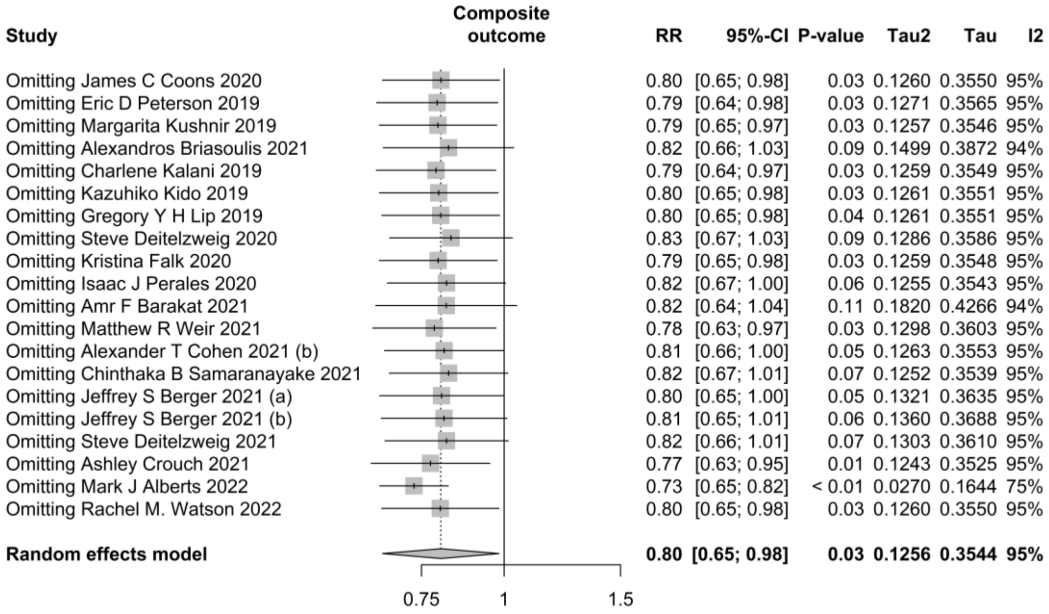

Figure S75. Leave-one-out sensitivity analysis for the composite primary efficacy outcome representing the comparison between direct oral anticoagulants (DOACs) and warfarin. RR, risk ratio; CI, confidence interval.

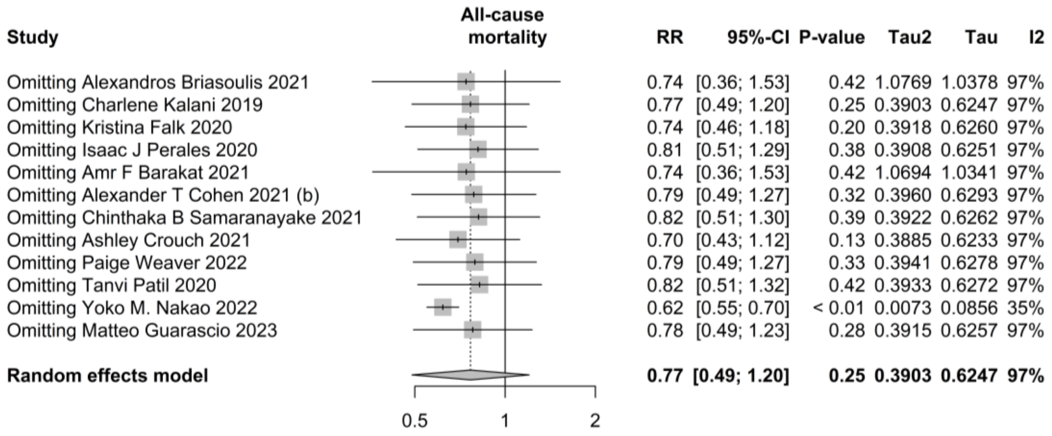

Figure S76. Leave-one-out sensitivity analysis for the outcome of all-cause mortality representing the comparison between direct oral anticoagulants (DOACs) and warfarin. RR, risk ratio; CI, confidence interval.

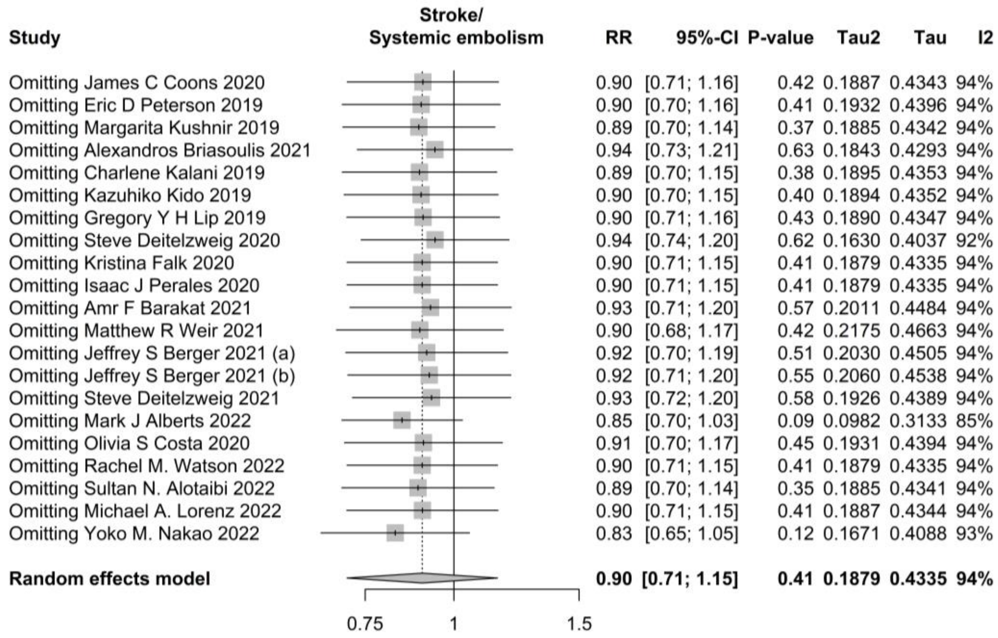

Figure S77. Leave-one-out sensitivity analysis for the outcome of any stroke or systemic embolism representing the comparison between direct oral anticoagulants (DOACs) and warfarin. RR, risk ratio; CI, confidence interval.

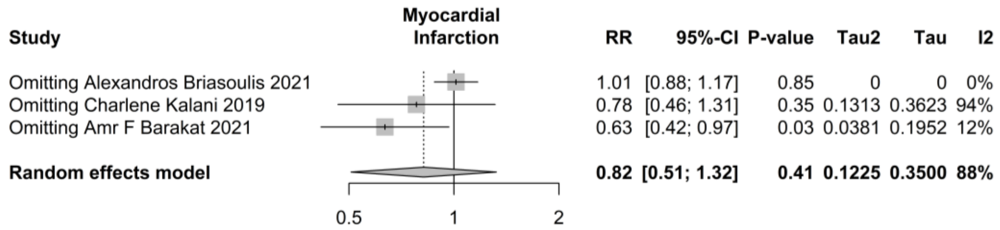

Figure S78. Leave-one-out sensitivity analysis for the outcome of myocardial infarction representing the comparison between direct oral anticoagulants (DOACs) and warfarin. RR, risk ratio; CI, confidence interval.

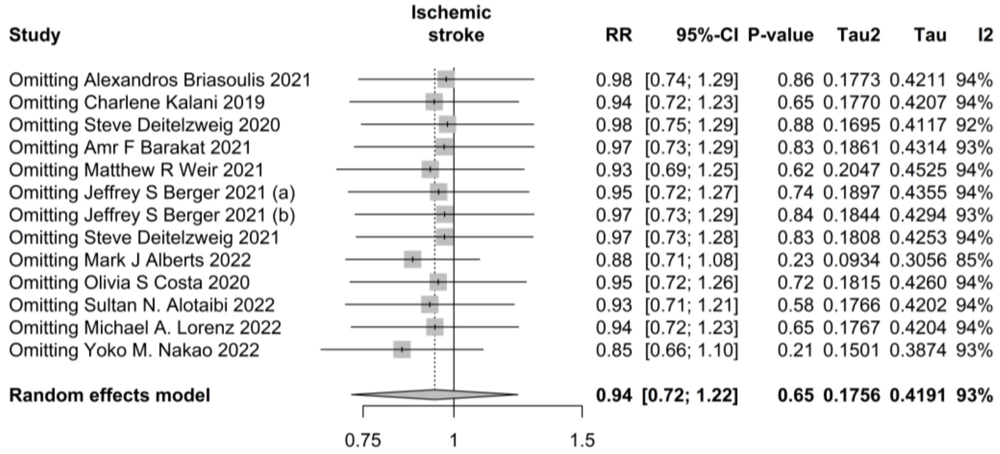

Figure S79. Leave-one-out sensitivity analysis for the outcome of ischemic stroke representing the comparison between direct oral anticoagulants (DOACs) and warfarin. RR, risk ratio; CI, confidence interval.

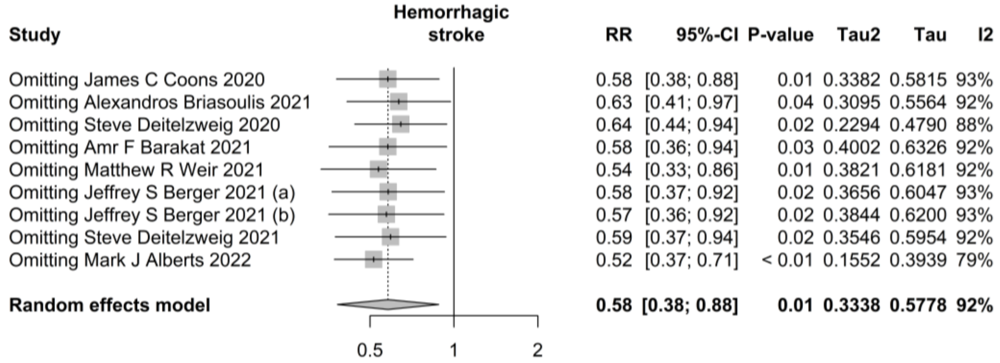

Figure S80. Leave-one-out sensitivity analysis for the outcome of hemorrhagic stroke representing the comparison between direct oral anticoagulants (DOACs) and warfarin. RR, risk ratio; CI, confidence interval.

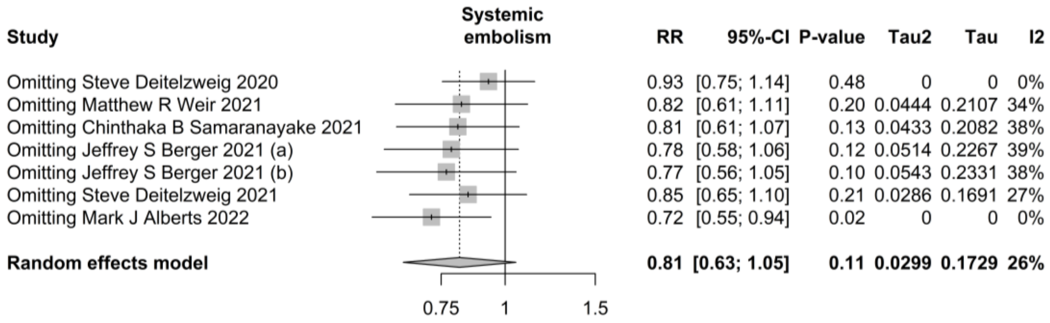

Figure S81. Leave-one-out sensitivity analysis for the outcome of systemic embolism representing the comparison between direct oral anticoagulants (DOACs) and warfarin. RR, risk ratio; CI, confidence interval.

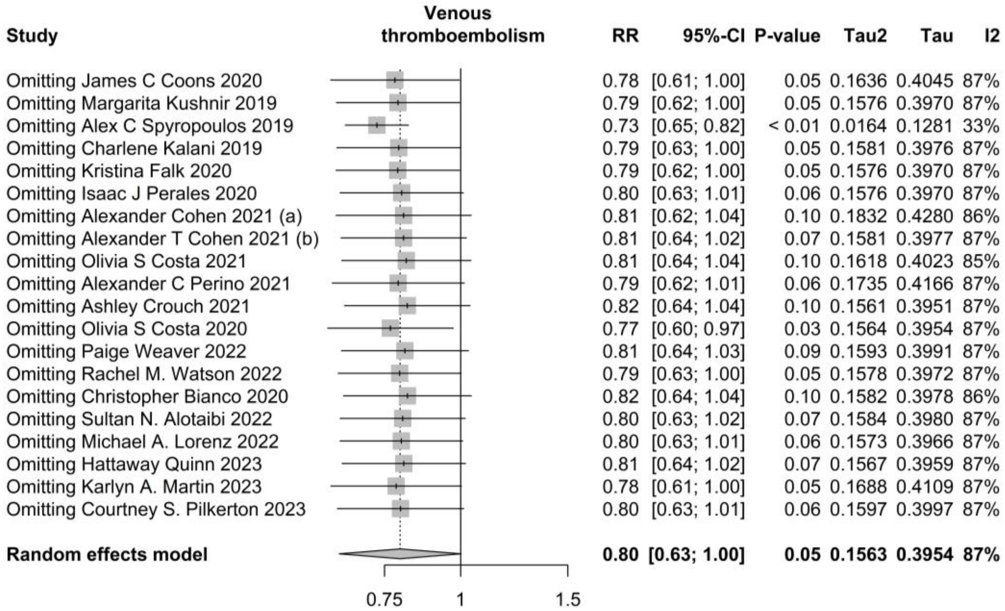

Figure S82. Leave-one-out sensitivity analysis for the outcome of venous thromboembolism representing the comparison between direct oral anticoagulants (DOACs) and warfarin. RR, risk ratio; CI, confidence interval.

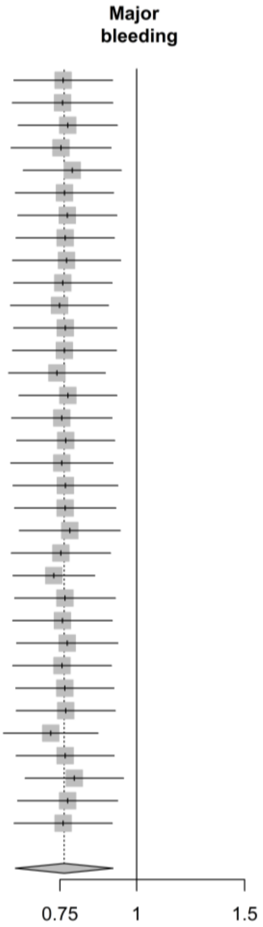

Study	RR	95%-CI	P-value	Tau2	Tau	I2
Omitting James C Coons 2020	0.76	[0.63; 0.91]	< 0.01	0.1898	0.4357	94%
Omitting Eric D Peterson 2019	0.76	[0.63; 0.91]	< 0.01	0.1926	0.4389	94%
Omitting Margarita Kushnir 2019	0.77	[0.64; 0.93]	< 0.01	0.1898	0.4356	94%
Omitting Alex C Spyropoulos 2019	0.75	[0.62; 0.91]	< 0.01	0.1908	0.4368	94%
Omitting Alexandros Briasoulis 2021	0.79	[0.65; 0.94]	0.01	0.1798	0.4241	93%
Omitting Charlene Kalani 2019	0.76	[0.63; 0.92]	< 0.01	0.1898	0.4357	94%
Omitting Kazuhiko Kido 2019	0.77	[0.64; 0.93]	< 0.01	0.1898	0.4356	94%
Omitting Gregory Y H Lip 2019	0.76	[0.64; 0.92]	< 0.01	0.1898	0.4356	94%
Omitting Steve Deitelzweig 2020	0.77	[0.63; 0.94]	0.01	0.2294	0.4789	93%
Omitting Kristina Falk 2020	0.76	[0.63; 0.91]	< 0.01	0.1897	0.4356	94%
Omitting Isaac J Perales 2020	0.75	[0.62; 0.90]	< 0.01	0.1881	0.4337	94%
Omitting Alexander Cohen 2021 (a)	0.76	[0.63; 0.93]	< 0.01	0.2047	0.4524	94%
Omitting Amr F Barakat 2021	0.76	[0.63; 0.93]	< 0.01	0.2083	0.4564	94%
Omitting Matthew R Weir 2021	0.74	[0.62; 0.89]	< 0.01	0.1745	0.4177	93%
Omitting Alexander T Cohen 2021 (b)	0.77	[0.64; 0.93]	< 0.01	0.1884	0.4340	94%
Omitting Olivia S Costa 2021	0.76	[0.63; 0.91]	< 0.01	0.1920	0.4381	94%
Omitting Chinthaka B Samaranayake 2021	0.77	[0.64; 0.92]	< 0.01	0.1895	0.4353	94%
Omitting Jeffrey S Berger 2021 (a)	0.76	[0.62; 0.92]	< 0.01	0.2004	0.4477	94%
Omitting Jeffrey S Berger 2021 (b)	0.77	[0.63; 0.93]	< 0.01	0.2130	0.4616	94%
Omitting Alexander C Perino 2021	0.76	[0.63; 0.92]	< 0.01	0.1957	0.4424	94%
Omitting Steve Deitelzweig 2021	0.78	[0.64; 0.94]	< 0.01	0.1935	0.4399	93%
Omitting Ashley Crouch 2021	0.75	[0.62; 0.91]	< 0.01	0.1896	0.4355	94%
Omitting Mark J Alberts 2022	0.73	[0.63; 0.85]	< 0.01	0.1138	0.3373	89%
Omitting Olivia S Costa 2020	0.76	[0.63; 0.92]	< 0.01	0.1935	0.4399	94%
Omitting Paige Weaver 2022	0.76	[0.63; 0.91]	< 0.01	0.1902	0.4362	94%
Omitting Christopher Bianco 2020	0.77	[0.64; 0.93]	< 0.01	0.1956	0.4423	94%
Omitting Sultan N. Alotaibi 2022	0.76	[0.63; 0.91]	< 0.01	0.1897	0.4355	94%
Omitting Michael A. Lorenz 2022	0.76	[0.64; 0.92]	< 0.01	0.1897	0.4356	94%
Omitting Tanvi Patil 2020	0.77	[0.64; 0.92]	< 0.01	0.1901	0.4359	94%
Omitting Yoko M. Nakao 2022	0.72	[0.61; 0.86]	< 0.01	0.1654	0.4067	93%
Omitting Hattaway Quinn 2023	0.76	[0.64; 0.92]	< 0.01	0.1896	0.4354	94%
Omitting Karlyn A. Martin 2023	0.79	[0.66; 0.95]	0.01	0.1854	0.4306	94%
Omitting Courtney S. Pilkerton 2023	0.77	[0.64; 0.93]	< 0.01	0.1910	0.4370	94%
Omitting Matteo Guarascio 2023	0.76	[0.63; 0.91]	< 0.01	0.1896	0.4354	94%
**Random effects model**	**0.76**	**[0.63; 0.92]**	**< 0.01**	**0.1893**	**0.4351**	**94%**

Figure S83. Leave-one-out sensitivity analysis for the outcome of major bleeding representing the comparison between direct oral anticoagulants (DOACs) and warfarin. RR, risk ratio; CI, confidence interval.

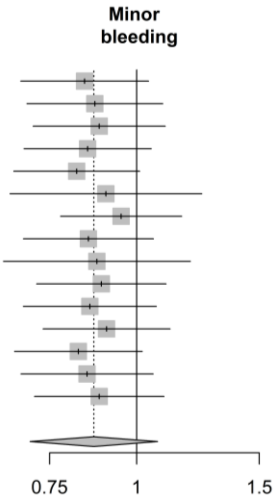

Study	RR	95%-CI	P-value	Tau2	Tau	I2
Omitting James C Coons 2020	0.84	[0.68; 1.04]	0.11	0.0619	0.2488	76%
Omitting Margarita Kushnir 2019	0.87	[0.70; 1.09]	0.23	0.0689	0.2625	77%
Omitting Kazuhiko Kido 2019	0.88	[0.71; 1.10]	0.27	0.0670	0.2588	77%
Omitting Gregory Y H Lip 2019	0.85	[0.69; 1.05]	0.13	0.0627	0.2505	76%
Omitting Kristina Falk 2020	0.82	[0.67; 1.01]	0.06	0.0562	0.2371	74%
Omitting Alexander Cohen 2021 (a)	0.90	[0.66; 1.24]	0.53	0.2066	0.4545	71%
Omitting Alexander T Cohen 2021 (b)	0.95	[0.78; 1.16]	0.61	0.0463	0.2153	70%
Omitting Chinthaka B Samaranayake 2021	0.85	[0.69; 1.06]	0.15	0.0653	0.2555	77%
Omitting Alexander C Perino 2021	0.88	[0.64; 1.19]	0.40	0.1909	0.4370	71%
Omitting Ashley Crouch 2021	0.89	[0.72; 1.10]	0.29	0.0647	0.2544	77%
Omitting Paige Weaver 2022	0.86	[0.69; 1.07]	0.17	0.0669	0.2587	77%
Omitting Sultan N. Alotaibi 2022	0.91	[0.73; 1.12]	0.35	0.0600	0.2449	75%
Omitting Michael A. Lorenz 2022	0.82	[0.67; 1.02]	0.07	0.0588	0.2424	75%
Omitting Tanvi Patil 2020	0.85	[0.68; 1.06]	0.14	0.0657	0.2563	77%
Omitting Hattaway Quinn 2023	0.88	[0.71; 1.09]	0.26	0.0656	0.2560	77%
**Random effects model**	**0.87**	**[0.70; 1.07]**	**0.19**	**0.0650**	**0.2549**	**76%**

Figure S84. Leave-one-out sensitivity analysis for the outcome of minor bleeding representing the comparison between direct oral anticoagulants (DOACs) and warfarin. RR, risk ratio; CI, confidence interval.

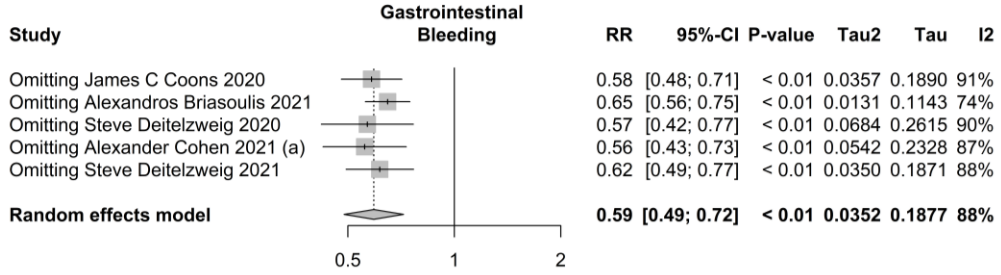

Figure S85. Leave-one-out sensitivity analysis for the outcome of gastrointestinal bleeding representing the comparison between direct oral anticoagulants (DOACs) and warfarin. RR, risk ratio; CI, confidence interval.

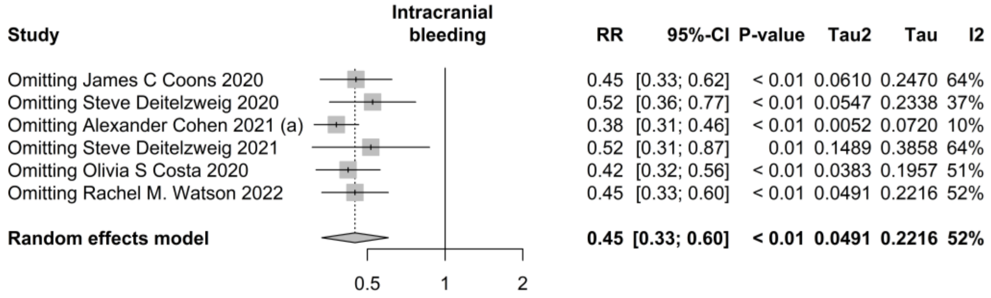

Figure S86. Leave-one-out sensitivity analysis for the outcome of intracranial bleeding representing the comparison between direct oral anticoagulants (DOACs) and warfarin. RR, risk ratio; CI, confidence interval.

Table S5. Results of univariate meta-regression for major bleeding and the composite outcome

	Composite outcome					All-cause mortality					Major bleeding				
Covariate	Studies (N)	Coefficient with 95% CI	p-value	R^2	I^2	Studies (N)	Coefficient with 95% CI	p-value	R^2	I^2	Studies (N)	Coefficient with 95% CI	p-value	R^2	I^2
**Mean age (years)**	20	0.99 (0.96, 1.03)	0,628	0	94,27	10	1.03 (0.97, 1.1)	0,357	0	96,9	29	1.01 (0.98, 1.05)	0,529	0	94,46
**Men (%)**	19	0.45 (0.14, 1.47)	0,185	10,1	93,52	10	0.4 (0.03, 5.1)	0,480	0	97	29	0.46 (0.18, 1.18)	0,106	11,1	93,61
**Hypertension (%)**	11	1.06 (0.08, 13.55)	0,962	0	96,66	5	2.77 (0.01, 1246.1)	0,744	0	99	18	1.48 (0.24, 9.27)	0,675	0	96,66
**Diabetes (%)**	12	1.72 (0.25, 11.82)	0,582	0	96,39	8	0.27 (0, 143.2)	0,680	0	98,1	19	2.15 (0.32, 14.31)	0,43	0	95,78
**Dyslipidaemia (%)**	8	1.17 (0.12, 11.1)	0,89	0	97,97	N/A	N/A	N/A	N/A	N/A	10	2.03 (0.21, 19.33)	0,537	0	97,04
**Cancer (%)**	9	24.97 (3.32, 187.68)	**0,002**	86,9	56,82	9	9493 (3.07, 29400606.8)	**0,026**	71.5	82,3	14	2.79 (0.07, 104.99)	0,58	36,8	88,59
**Renal disease (%)**	11	1.38 (0.55, 3.43)	0,491	0	84,94	5	903.9 (51.2, 15955.2)	**<0.001**	80.5	93,3	17	2.58 (0.62, 10.81)	0,193	0	94,26
**Stroke (%)**	12	9.36 (0.06, 1561.33)	0,392	0	96,73	4	0.08 (0, 91607.5)	0,719	0	79,9	15	9.27 (0.13, 654.91)	0,305	16,8	95,9

Coefficients with 95% confidence intervals (CI) are presented in exponentiated form. P-values of statistically significant associations are marked in bold. N/A, not applicable;
